# Developing Qualitative Plasmid DNA Reference Materials to Detect Mechanisms of Quinolone and Fluoroquinolone Resistance in Foodborne Pathogens

**DOI:** 10.3390/foods11020154

**Published:** 2022-01-07

**Authors:** Qinya Niu, Xiumin Su, Luxin Lian, Jinling Huang, Shutong Xue, Wei Zhou, Hongyang Zhao, Xing’an Lu, Shenghui Cui, Jia Chen, Baowei Yang

**Affiliations:** 1 College of Food Science and Engineering, Northwest A&F University, Xianyang 712100, China; nqy736461094@163.com (Q.N.); sxm10182021@163.com (X.S.); llxin1116@163.com (L.L.); xncyxyxgb@163.com (J.H.); pcyxst20000714@163.com (S.X.); 2 Hebei Food Inspection and Research Institute, Hebei Food Safety Key Laboratory, Shijiazhuang 050091, China; zhouwei0311@163.com; 3 Chinese Academy of Inspection and Quarantine, Beijing 100176, China; zhy8707@163.com (H.Z.); luxa@sina.com (X.L.); 4 National Institutes for Food and Drug Control, Beijing 100050, China; cuishenghui@aliyun.com; 5 College of Chemical Technology, Shijiazhuang University, Shijiazhuang 050035, China; chenjia_0311@163.com

**Keywords:** antibiotic resistance, plasmid DNA reference material, limit of detection, homogeneity, stability

## Abstract

The aim of this study was to develop homogeneous and stable plasmid DNA reference materials for detecting the mechanisms of resistance to quinolones and fluoroquinolones in foodborne pathogens. The DNA fragments of 11 target genes associated with quinolone and fluoroquinolone resistance were artificially synthesized, inserted into plasmid vectors, and transferred into recipient cells. PCR and sequencing of DNA were performed to assess the genetic stability of the target DNA in recombinant *Escherichia coli* DH5α cells during subculturing for 15 generations. The limit of detection (LOD) of the target DNA was determined using PCR and real-time qualitative PCR (qPCR). The homogeneity and storage stability of plasmid DNA reference materials were evaluated in terms of plasmid DNA quantity, PCR-measured gene expression, and qPCR threshold cycle. All 11 target DNAs were successfully synthesized and inserted into vectors to obtain recombinant plasmids. No nucleotide mutations were identified in the target DNA being stably inherited and detectable in the corresponding plasmids during subculturing of recombinant strains. When the target DNA was assessed using PCR and qPCR, the LOD was ≤1.77 × 10^5^ and 3.26 × 10^4^ copies/μL, respectively. Further, when the reference materials were stored at 37 °C for 13 days, 4 °C for 90 days, and −20 °C for 300 days, each target DNA was detectable by PCR, and no mutations were found. Although the threshold cycle values of qPCR varied with storage time, they were above the LOD, and no significant differences were found in the quantity of each plasmid DNA at different timepoints. Further, the homogeneity and stability of the materials were highly consistent with the requirements of standard reference materials. To summarize, considering that our plasmid DNA reference materials conformed to standard requirements, they can be used to detect the mechanisms of quinolone and fluoroquinolone resistance in foodborne pathogens.

## 1. Introduction

According to the World Health Organization, nearly one-tenth of the global population get sick from foodborne diseases each year [1]. This consequently leads to 420,000 deaths annually, with the main reason being consumption of food contaminated with foodborne pathogens [2,3]. Antibiotics are the standard and the most direct, effective method to treat foodborne diseases, but antibiotic resistance is becoming an increasingly serious issue.

Quinolones (i.e., nalidixic acid) and fluoroquinolones (i.e., ciprofloxacin) are important synthetic antibiotics that are commonly used to treat diseases caused by foodborne pathogens. However, mutations in the quinolone resistance-determining region of DNA gyrase subunits (GyrA and GyrB) and topoisomerase IV subunits (ParC and ParE) have been associated with quinolone and fluoroquinolone resistance. Moreover, the presence of *qnrA*, *qnrB*, *aac(6′)-Ib-cr*, *oqxAB*, and *qnrS* genes, which encode hydrolases and antibiotic-inactivating enzymes, in the genome of pathogens has been associated with fluoroquinolone resistance [4,5,6,7]. As a part of food safety inspection and for diagnosing clinical diseases caused by foodborne pathogens, homogeneous and stable plasmid DNA reference materials are required to ensure data accuracy when detecting the mechanisms of quinolone and fluoroquinolone resistance.

At present, >1000 biological reference materials exist in the list of the National Institute for Biological Standards and Control (https://www.nibsc.org/, accessed on 4 January 2021), the leading international standard and reference material producer and distributor in the world. However, serums, vaccines, and antibodies are the most prevalent reference materials [8]; the prevalence of viruses and genetically modified products as reference materials remains scarce. In China, despite there being some nucleic acid reference materials, they are mainly related to viruses and genetically modified products (https://www.ncrm.org.cn/Web/Home/Index, accessed on 4 January 2021). In recent years, although many studies in China have reported the development of reference materials to detect the DNA of bacterial pathogens, the adoption of these materials for technical purposes has been limited due to traceability and practicality. Consequently, the application of these materials for technical purposes has been limited [9,10,11]. To the best of our knowledge, to date, there exists no DNA reference material and/or certified reference material that can be used to identify genes responsible for antibiotic resistance and/or to assess pertinent mechanisms.

In this study, we developed 11 qualitative plasmid DNA reference materials to study mechanisms associated with quinolone and fluoroquinolone resistance in foodborne pathogens for food safety inspection and molecular diagnosis of clinical diseases caused by foodborne pathogens.

## 2. Materials and Methods

### 2.1. Target DNA Synthesis and Recombinant Plasmid and Strain Construction

**Figure 1:**
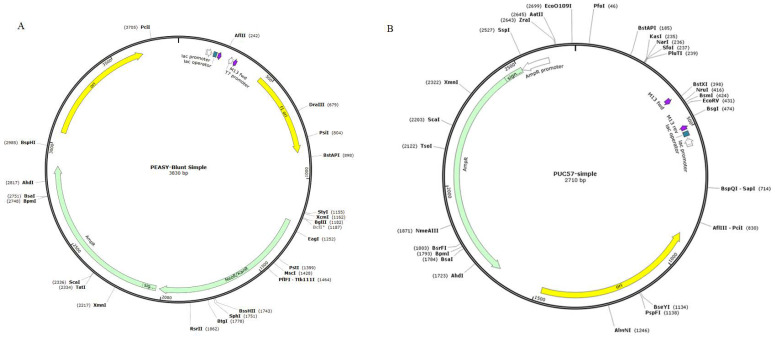
Schematic diagram of plasmid vectors. (**A**) pEASY. (**B**) pUC57.

We screened the following: five genes (*qnrA*, *qnrB*, *qnrS*, *aac(6′)-Ib*, and *oqxA*) that are commonly found in plasmids and six genes (*gyrA* with the single mutations Asp87Asn, Ser83Tyr, and Ser83Phe in GyrA; *gyrA* with the double mutations Ser83Phe/Asp87Gly and Ser83Phe/Asp87Ala in GyrA; and *parC* with the single mutation Ser80Arg in ParC) that are associated with quinolone and fluoroquinolone resistance from the GenBank nucleic acid sequence database of the National Center for Biotechnology Information (NCBI; http://www.ncbi.nlm.nih.gov, accessed on 4 December 2018). The sequences of each gene were downloaded from NCBI website and compared using BioEdit (BioEdit Inc., Manchester, NH, USA). The gene fragment with the same DNA sequence was defined as the target DNA. The DNA fragment of the target DNA was synthesized by Beijing AuGCT Co., Ltd. (Beijing, China), and subsequently inserted into pEASY and pUC57 to construct recombinant plasmids (Figure 1). The recombinant plasmids were then transferred into *Escherichia coli* DH5α cells to obtain recombinant strains.

### 2.2. Genetic Stability Test of the Target DNA in Recombinant Strains

All recombinant strains were plated on Luria-Bertani agar (CM1552, Beijing Land Bridge Technology Co., Ltd., Beijing, China). After incubation at 37 °C ± 0.5 °C for 12–18 h, a single colony was chosen and continuously subcultured for 15 generations on Luria-Bertani agar plates. Target DNA stability in the recombinant strains was determined by performing PCR every three generations. DNA sequencing and online BLAST alignment were performed to determine whether the target DNA was mutated.

Template DNA was prepared as previously described [12]. PCR was performed on a MyCircle PCR system (Bio-Rad, Hercules, CA, USA) in a 25-μL reaction mixture containing 13.15 μL of double-distilled (dd) H_2_O, 0.3 μL of 50 ng/mL primer each, 2.5 μL of 10×PCR buffer (25 mM without Mg^2+^; R001AM, TaKaRa, Dalian, China), 2 μL of 2.5 mM dNTP mixture (TaKaRa, Dalian, China, 0.25 μL of 5 U/μL Taq DNA polymerase (TaKaRa, Dalian, China), 1.5 μL of 25 mM MgCl_2_ (TaKaRa, Dalian, China), and 5 μL of template DNA. The cycling conditions were as follows: initial denaturation at 94 °C for 5 min; followed by 35 cycles of 94 °C for 30 s, pertinent annealing temperature for 30 s, and 72 °C for 1 min; and final extension at 72 °C for 10 min [13]. Table 1 lists all primers and annealing temperatures for each target DNA.

The amplicons obtained were electrophoresed and visualized under UV light, and they were then transferred at low temperatures for sequencing to AuGCT Biotech (Beijing, China). The DNA sequence was aligned with the original sequence using the online BLAST program to determine whether the gene(s) had mutated in the subcultures. To detect mutations of *gyrA* and *parC*, the DNA sequence was translated into the corresponding amino acid sequence using Primer Premier 5 (Premier Biosoft, San Francisco, CA, USA) and aligned to ascertain that the preset mutation sites were stably inherited during the subculture.

### 2.3. Extraction of Plasmids Carrying Antibiotic Resistance-Encoding Genes

Plasmid extraction was performed using a Plasmid Mini Kit I (D6943-01*, OMEGA, Norcross, GA, USA), according to manufacturer’s instructions. Plasmid DNA concentration was measured with Qubit^TM 4^ (Thermo Fisher Scientific, Waltham, MA, USA), and plasmid DNA was stored at −20 °C until needed.

### 2.4. Assessment of Limit of Detection (LOD) for the Target DNA

We serially diluted 10 μL of the plasmid suspension plus target DNA using 90 μL of sterile water by 10-fold each time (10^−1^, 10^−2^, and so on) until the suspension was diluted to 10^−*n*^ concentration. All these different dilutions served as template DNA for PCR and real-time quantitative PCR (qPCR). The LOD of PCR was calculated via the initial concentration of the target DNA solution divided by the maximum dilution times when the amplicon on the electrophoresis gel appeared markedly dark, unclear, or invisible. The PCR amplification system and conditions were the same as those described earlier (under *Genetic Stability of the Target DNA in Recombinant Strains*). Table 1 lists pertinent primers and annealing temperatures used for PCR.

qPCR was performed on a Bio-Rad iQ5 thermal cycler in a 25-μL reaction mixture comprising 8.5 μL of ddH_2_O, 12.5 μL of 2 × SYBR Green *Pro Taq* HS Premix (AG11701, AG, Changsha, China), 1 μL of 50 ng/mL primer each, and 2 μL of template with different concentrations of the recombinant plasmid. The cycling conditions were as follows: 95 °C for 30 s; 40 cycles at 95 °C for 5 s and 60 °C for 30 s; 1 cycle at 95 °C for 15 s and 60 °C for 30 s; and 71 cycles with the temperature increasing from 60 °C to 95 °C. Primers used for qPCR are listed in Table 2. The LOD of qPCR was determined as the concentration of the recombinant plasmid solution (i.e., the template) that did not cause any further increase in the threshold cycle (Ct) value [19].

The copy number of plasmid DNA that determined LOD was calculated using the following formula:

Number of copy (copy/μL) = (Concentration × 10^−9^ × 6.02 × 10^23^)/(Length × 660)

where Concentration represents plasmid DNA concentration measured using a NanoDrop One spectrophotometer (Thermo Fisher Scientific, Waltham, MA, USA; ng/μL) and Length represents template DNA length (bp).

### 2.5. Preparation of Plasmid DNA Reference Materials

The recombinant plasmid with the target DNA was extracted from the recombinant strain using a Plasmid Mini Kit I (OMEGA, Norcross, GA, USA). The concentration and OD260/280 and OD260/230 values of the plasmid DNA suspension were determined using the NanoDrop One spectrophotometer. The suspension was subsequently subpackaged in a 1.5-mL Eppendorf tube; the quantity of the recombinant plasmid in each tube was approximately 300 ng. The DNA was then vacuum dried to prepare reference materials.

### 2.6. Homogeneity Test of Plasmid DNA Reference Materials

According to the guidelines of China National Standard GB/T 15000.3-2008 “Directives for the work of reference materials (3)—Reference materials—General and statistical principles for certification” and ISO Guide 35:2006 “Reference materials—General and statistical principles for certification, IDT”, when the total number of the units of reference materials is less than 500, the unit number selected for the homogeneity test should not be less than 10. We consequently selected 12 Eppendorf tubes of reference material samples at random for the homogeneity test, with each tube serving as a sample unit. After re-dissolution, the concentration of the plasmid DNA sample was determined using the NanoDrop One spectrophotometer. The quantity of DNA sample was calculated as plasmid DNA concentration × aqueous solution volume.

### 2.7. Storage Stability Test of Plasmid DNA Reference Materials

After vacuum drying, plasmid DNA was stored at 37 °C, 4 °C, and −20 °C to evaluate its storage stability. The stability of plasmid DNA reference materials stored at 37 °C was tested over 13 days (short term), and random samples were taken every week. Similarly, the stability of plasmid DNA reference materials stored at 4 °C was tested over 90 days (short term); samples were taken every week for the first 2 weeks (1, 7, and 13 days) and every month for the remaining period (30, 60, and 90 days). Finally, the stability of plasmid DNA reference materials stored at −20 °C was tested over 12 months (long term); from the first to the sixth month of storage, plasmid DNA was randomly sampled every month, and from the seventh to the twelfth month of storage, it was sampled every 2 months. All samples stored at different temperatures were sampled and tested in triplicate.

The indicators for the storage stability test were DNA quantity, PCR-measured gene expression, qPCR Ct value, DNA sequence, and amino acid mutation. PCR/qPCR primers, amplification system, and conditions were the same as those described earlier (see Section 2.2 and Section 2.4, respectively).

### 2.8. Data Analysis

Microsoft Office Excel v2010 (Microsoft Corp., Redmond, WA, USA) was used for the basic processing of experimental data. IBM SPSS Statistics v22 (IBM, New York, NY, USA) was used for statistical analysis of variance (ANOVA; Duncan’s method, *p* ≤ 0.05 indicating the difference being statistically significant). RStudio v3.4.4 (RStudio Inc., Boston, MA, USA) was used for drawing graphs.

## 3. Results

### 3.1. Recombinant Plasmid and Strain Construction

The target DNA fragments of *qnrA*, *qnrB*, *qnrS*, *aac(6′)-Ib*, *oqxA*, *gyrA* with the single mutations Asp87Asn, Ser83Tyr, and Ser83Phe (i.e., *gyrA*1, *gyrA*2, and *gyrA*3, respectively) in GyrA, *gyr*A with the double mutations Ser83Phe/Asp87Gly and Ser83Phe/Asp87Ala (i.e., *gyrA*4 and *gyrA*5, respectively) in GyrA, and *parC* with the single mutation Ser80Arg in ParC were all successfully synthesized and ligated into pUC57 and pEASY-T. All recombinant plasmids were successfully transformed into *E. coli* DH5α cells and corresponding recombinant strains were obtained. DNA sequencing results indicated that the identities and coverage rates of the target DNA in the 11 recombinant plasmids were 100%, as anticipated (Table S1). The nucleotide sequences of all target DNA fragments were submitted to GenBank and issued an accession number to ensure traceability of the reference materials (Table S1).

### 3.2. Genetic Stability

The target DNA in all recombinant strains was stably inherited across all 15 generations, and no mutations were found (Figures S1–S7). Further, the single and double mutations in *gyrA* and *parC* were stably transferred from the first to the last (*n* = 15) generation (Figure 2).

### 3.3. LOD of PCR and qPCR

**Figure 3:**
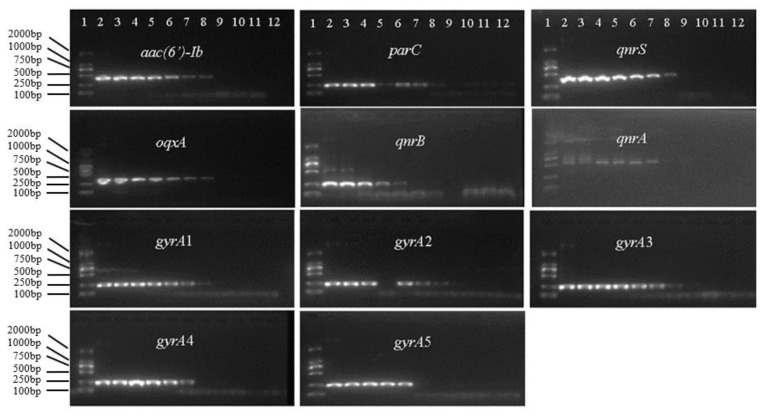
Limit of detection of PCR for the target DNA. In all gels: lane 1, DL 2000 DNA marker; lanes 2–11, amplicons obtained using template DNA (10^1^–10^10^-fold diluted); lane 12, double-distilled H_2_O, which served as the blank control.

Spectrophotometric data indicated that all OD260/280 values for the plasmid DNA ranged from 1.8 to 2.0 and all OD260/230 values were between 2.0 and 2.2, implying that the purity of plasmid DNA extracted from the recombinant strains adequately met the requirements of standard reference materials. With regard to the LOD of PCR, it was 1.85 × 10^3^ copies/μL for *aac(6′)-Ib*, 1.37 × 10^4^ copies/μL for *gyrA*2, 1.44 × 10^4^ copies/μL for *gyrA*3, 1.88 × 10^4^ copies/μL for *parC*, 1.97 × 10^4^ copies/μL for *gyrA*4, 2.02 × 10^4^ copies/μL for *gyrA*1, 2.13 × 10^4^ copies/μL for *qnrA*, 2.19 × 10^4^ copies/μL for *qnrS*, 3.26 × 10^4^ copies/μL for *oqxA*, 1.74 × 10^5^ copies/μL for *qnrB*, and 1.77 × 10^5^ copies/μL for *gyrA*5 (Figure 3). The LOD and copy number of all plasmid DNAs decreased with an increase in dilution times. These results indicated that when the target DNA was assessed using PCR, the LOD was ≤10^5^ copies/μL.

**Figure 4:**
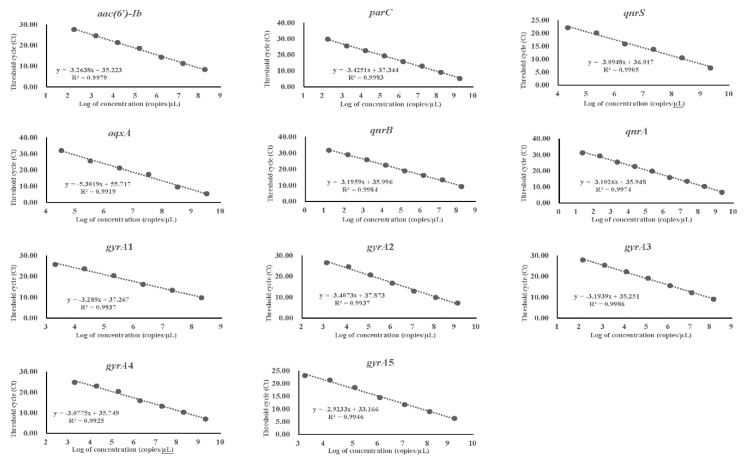
Standard curve and Ct values of qPCR for each target DNA. X axis represents logarithm of concentration, and Y axis represents Ct values for different concentrations of template DNA.

With regard to the LOD of qPCR, it was 17.4 copies/μL for *qnrB*, 21.3 copies/μL for *qnrA*, 1.44 × 10^2^ copies/μL for *gyrA*3, 1.85 × 10^2^ copies/μL for *aac(6′)-Ib*, 1.88 × 10^2^ copies/μL for *parC*, 1.37 × 10^3^ copies/μL for *gyrA*2, 1.77 × 10^3^ copies/μL for *gyrA*5, 1.97 × 10^3^ copies/μL for *gyrA*4, 2.02 × 10^3^ copies/μL for *gyrA*1, 2.19 × 10^4^ copies/μL for *qnrS*, and 3.26 × 10^4^ copies/μL for *oqxA*. These results indicated that based on qPCR, the LOD of the target DNA was ≤10^4^ copies/μL. For qPCR, the standard curve was constructed using Ct values (Figure 4). We found that there was an excellent linear relationship between the template DNA concentration and Ct value, with all regression coefficients (R^2^) being >0.99.

### 3.4. Homogeneity of Plasmid DNA Reference Materials

Gel electrophoresis results indicated that the target genes in the plasmid DNA could be successfully amplified, and the amplicons were of expected size (Figure S8). Sequencing data revealed that no mutations were present in the target DNA (Figures S9–S15). According to statistical analyses, the quantity of plasmid DNA harboring the target gene in each tube had an F value that was less than the F-critical value under 95% confidence interval; this result indicated that there were no significant differences in plasmid DNA quantity in each tube (Table 3). Collectively, these data showed that the homogeneity of plasmid DNA reference materials adequately met the requirements of standard reference materials.

### 3.5. Storage Stability of Plasmid DNA Reference Materials

**Figure 5:**
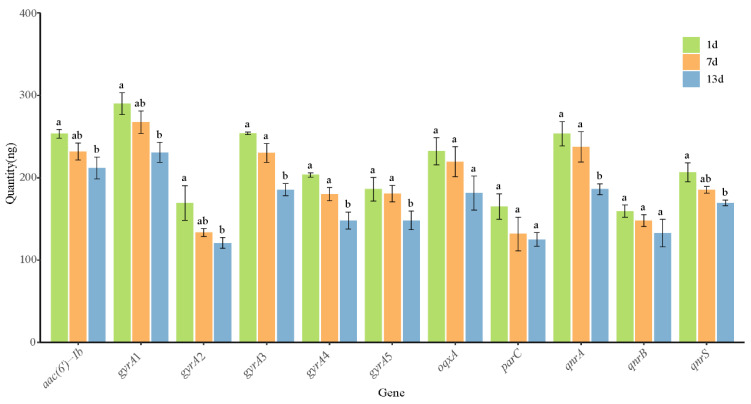
Short-term stability of the quantity of plasmid DNA reference materials stored at 37 °C. For each gene, columns labeled with the same letter indicates that no significant difference was found in plasmid DNA quantity upon storage for different durations.

With regard to the short-term storage stability of plasmid DNA reference materials, no significant differences in plasmid DNA quantity were found for *aac(6′)-Ib*, *qnrA*, *qnrS*, *gyrA*1, *gyrA*2, *gyrA*3, *gyrA*4, and *gyrA*5 upon storage at 37 °C for 7 days. Moreover, no significant differences in plasmid DNA quantity were observed for *oqxA*, *parC*, and *qnrB* upon storage for 13 days at 37 °C (Figure 5). All the 11 target DNAs were detectable by PCR within 13 days, and no mutations were found (Figures S16–S23). Although qPCR indicated that the Ct values for each target DNA varied in these 13 days, no significant differences were found in the Ct values for any target DNA (Table 4). To summarize, the storage stability of plasmid DNA reference materials was excellent when stored at 37 °C for at least 1 week, they can serve as positive standard samples to study genes and mechanisms associated with quinolone and fluoroquinolone resistance.

**Figure 6:**
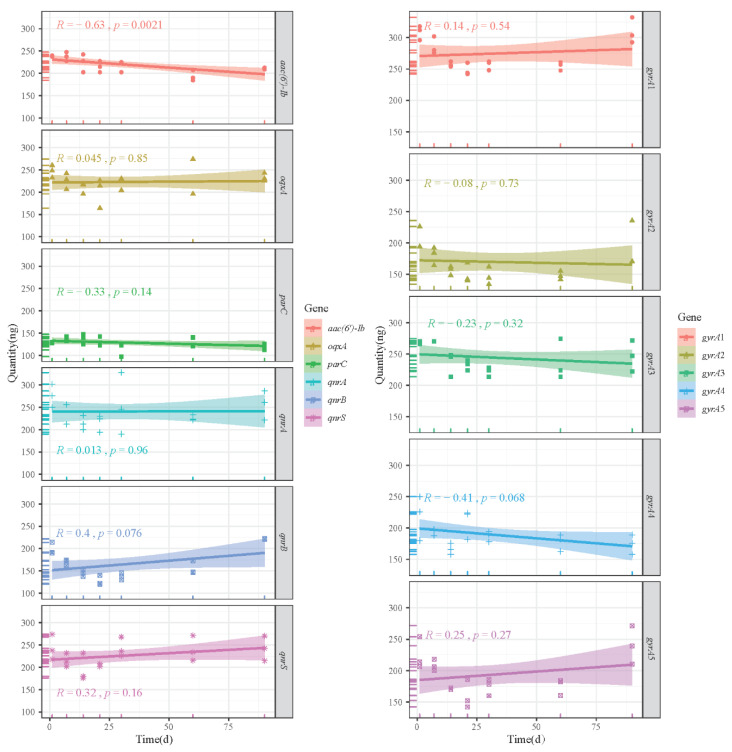
Short-term stability of the quantity of plasmid DNA reference materials stored at 4 °C. Note: “R” represents the correlation coefficient, “*p*” represents the significant difference, and the shadow represents the 95% confidence interval.

**Figure 7:**
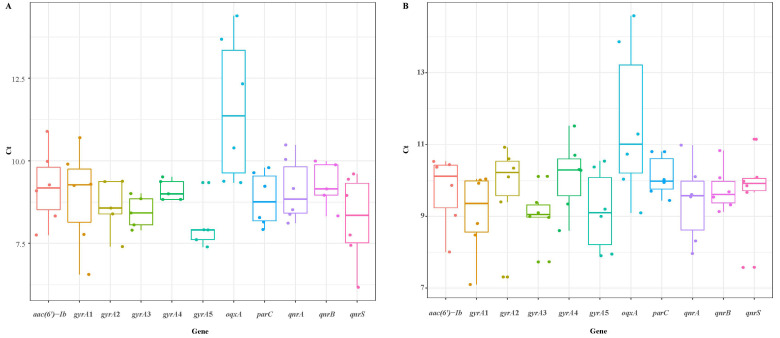
Ct values of qPCR with plasmid DNA reference materials stored at 4 °C and −20 °C. (**A**) Short-term storage stability at 4 °C. (**B**) Long-term storage stability at −20 °C.

When plasmid DNA reference materials were stored at 4 °C for 90 days, no significant differences were detected in plasmid DNA quantity for all target genes, with the exception of *aac(6′)-Ib* (Figure 6). Furthermore, all target genes were detectable by PCR within 90 days, and no mutations were identified (Figures S24–S31). There was no abnormal variation in the Ct values of qPCR after the samples were 10-fold serially diluted, and they remained within the LOD for each gene (Figure 7A). In summary, considering that most plasmid DNA reference materials exhibited excellent stability when stored for 90 days at 4 °C, they can serve as positive standard samples for studying genes and mechanisms associated with antibiotic resistance.

**Figure 8:**
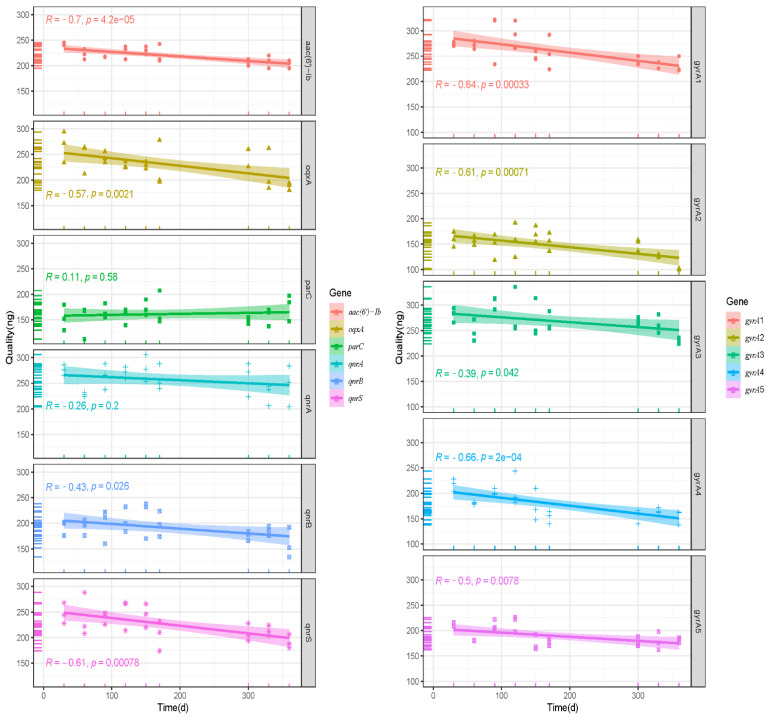
Long-term stability of the quantity of plasmid DNA reference materials stored at −20 °C. Note: “R” represents the correlation coefficient, “*p*” represents the significant difference, and the shadow represents the 95% confidence interval.

When plasmid DNA reference materials were stored at −20 °C for 1 year, the plasmid DNA quantity significantly (*p* ≤ 0.05) declined for all target genes, except *par*C (Figure 8). However, all target genes were detectable by PCR within 360 days, and no mutations were found (Figures S32–S39). The Ct values of qPCR considerably varied after plasmid DNA reference materials were 10-fold serially diluted, but they did not exceed the LOD for each gene (Figure 7B). Therefore, despite the decline in quantity upon storage for 360 days at −20 °C, all plasmid DNA reference materials can still serve as qualitative standard reference samples.

## 4. Discussion

Many molecular detection techniques, such as conventional PCR, qPCR, and loop-mediated isothermal amplification, are widely used to identify foodborne pathogens, determine antibiotic resistance dissemination, and study the potential mechanisms of action based on food safety perspective [20,21,22,23,24]. To obtain accurate results, using reference materials is advisable during pathogen resistance gene detection and testing. At present, available reference materials used for microbial detection are mainly for viruses, whereas those for DNA detection are mainly in the field of transgenic food and crops (https://www.nibsc.org/). Although the demand for standard and certified reference materials has been constantly increasing to achieve food safety and for consumer health protection, no studies have as yet reported the development of plasmid DNA reference materials to identify antibiotic resistance-encoding genes and to elucidate pertinent mechanisms in foodborne pathogens. Herein we developed 11 qualitative plasmid DNA reference materials to study genes and mechanisms associated with quinolone and fluoroquinolone resistance. We believe that our findings should facilitate the detection and prevention of antibiotic resistance in foodborne pathogens.

Previously, the development of few reference strains belonging to *Salmonella*, *Shigella*, and *Cronobacter* has been reported to detect antibiotic resistance [9,25,26,27]. However, such pathogenic reference materials are a potential threat to food safety in some food production environments. Therefore, developing DNA reference materials for detecting some antibiotic resistance-encoding genes and associated mechanisms becomes pivotal. In the present study, 11 recombinant plasmids and strains associated with quinolone and fluoroquinolone resistance were successfully constructed, and antibiotic resistance-encoding genes in the recombinant plasmids and strains were found to be stably inherited even after subculturing for 15 generations. Moreover, no nucleotide mutations were detected in the target DNA, indicating that these plasmids and strains could potentially serve as reference materials.

PCR is commonly used for the rapid detection of antibiotic resistance-encoding genes in pathogenic bacteria. During reference material development, it is thus essential to determine the LOD of PCR for detecting target DNA in recombinant plasmids and it is also the premise for ensuring successful detection. Xia et al. developed a plasmid DNA reference material to detect pathogenic *E. coli*, and they found the LOD of PCR for *escV*, *stx2*, and *hlyA* to be 3.93 × 10^6^, 2.41 × 10^5^, and 2.14 × 10^5^ copies/μL, respectively [11]. Moreover, Ma et al. developed plasmid DNA reference materials to detect *Listeria monocytogenes*, and they found the LOD of PCR for *hlyA*, *prfA*1, and *prfA*2 to be 8.2 × 10^7^, 1.1 × 10^6^, and 1.24 × 10^5^ copies/μL, respectively [28]. In the present study, when plasmid DNA reference materials were detected using PCR, the LOD was ≤10^5^ copies/μL, being lower than the LOD of PCR reported in previous studies. To explain, if target DNA fragments are obtained with different methods or DNA purification kits, the template DNA quantity is expected to vary, resulting in variances in results and LOD despite the detection method being the same [29]. This accordingly prompted us to herein use a kit with assured quality to ensure DNA purity, so that the reference materials could be reliably used for further research.

The qPCR technique is also commonly used to study antibiotic resistance-encoding genes and pertinent mechanisms as this technique has several advantages, such as high specificity and sensitivity, no involvement of a dye, and much shorter turnaround time as compared to PCR [30,31]. To evaluate the suitability of our plasmid DNA reference materials for qPCR, we determined the LOD of qPCR for each target DNA. We found that the LOD of qPCR for the 11 target DNAs ranged from 1.74 × 10^1^ to 3.26 × 10^4^ copies/μL. For all cases, the LODs were less than or equal to the DNA concentration in the target DNA solution diluted 10^6^ times (or 10^9^ times for some cases). Similarly, Dorlass et al. reported that when SARS-CoV-2 RNA was diluted 10^7^ times, the positive detection rate of SYBR Green-based qPCR was 98.42% [30]. Furthermore, Fábio et al. developed a plasmid DNA reference material to quantify genetically modified common bean embrapa 5.1 and found that the lowest amount that could be reliably detected by qPCR was 10^3^ copies per reaction [32]. Wu et al. also developed a general plasmid reference material for screening genetically modified organisms by qPCR, and they found that the sensitivity of screening and taxon-specific assays ranged from 5 to 10 copies of pBI121-Screening plasmid [33]. In addition, when we assessed the reference materials using qPCR, a good linear relationship was found between the template DNA concentration and Ct value, indicating that plasmid DNA reference materials developed in this study may even be used as universal calibrators.

Our data further indicated that the homogeneity and stability of all plasmid DNA reference materials met the standard requirements formulated by China National Standard and ISO Guide. In a previous study, Zhang et al. prepared some recombinant pseudovirus particles carrying specific St. Louis encephalitis virus genes; the pseudovirus particles showed excellent thermal stability upon storage at 37 °C for 20 days, room temperature for 30 days, 4 °C for 60 days, and –20 °C for 90 days [34]. Furthermore, Junichi et al. prepared a DNA reference material for quality control of PCR testing and found that the reference DNA molecule did not show rapid degradation when the material was stored at 37 °C for 1 week [35]. Herein even we found that our reference materials were relatively stable; they were in fact more stable than those developed in previous studies upon storage at 37 °C for 13 days, 4 °C for 90 days, and −20 °C for 360 days. However, long-term stability test results showed that the quantity of our plasmid DNA reference materials deteriorated after storage for 300–360 days at −20 °C, although no significant differences were found within the first 300 days. Although the DNA quantity decreased after 300 days of storage, the long-term stability of our plasmid DNA reference materials was still much better than that of the materials developed in previous studies. For example, Vallejo et al. developed a genomic DNA reference material for *Salmonella enteritidis* detection; on storage at 4 °C and −20 °C for 9 months, high concentration dispersion and DNA quantity deterioration were detected over time [36]. In addition, although our qPCR data showed that the Ct values for each plasmid DNA reference material varied between different timepoints of storage, the Ct values were still within the LOD for a particular gene in the material before storage. Similar to the results of our study, Zhou et al. prepared and characterized a pseudoviral positive control for the nucleic acid detection of MERS-CoV, and they found that when the samples were stored at 4 °C, −20 °C, and −70 °C for 1 week, the Ct values acquired via qPCR showed variation [37]. Based on these observations, the homogeneity and stability of our plasmid DNA reference materials were found to be highly consistent with the requirements of standard reference materials.

Considering the reliability of our plasmid DNA reference materials, we approached seven laboratories to jointly certify and validate our results. As anticipated, all of the laboratories reported the same or similar results. To establish our plasmid DNA reference materials as certified reference materials, we plan to obtain a Chinese standard reference material number (GSB series) for them; once the GSB number is issued, we will try to obtain equivalent international mutual recognition, which should standardize the use of these materials to explore the mechanisms underlying resistance to quinolones and fluoroquinolones.

As reference materials, they can meet all requirements for non-standard methods validation, new methods evaluation, laboratory testing personnel assessment, laboratory testing capacity evaluation, and inter-laboratory comparison of reference materials [26]. As reference materials of antibiotic resistance genes, they are suitable for the validation of PCR and qPCR detection methods for antibiotic resistance genes identification in foodborne pathogens. In addition, these reference materials can serve as positive standard samples for genes and mechanisms associated with quinolone and fluoroquinolone resistance.

To conclude, herein we developed 11 plasmid DNA reference materials that showed excellent genetic stability, homogeneity, and storage stability. The materials can thus be used to detect and explore the mechanisms underlying quinolone and fluoroquinolone resistance in foodborne pathogens. Moreover, they can serve as qualitative and positive controls in future studies.

## Figures and Tables

**Figure 2:**
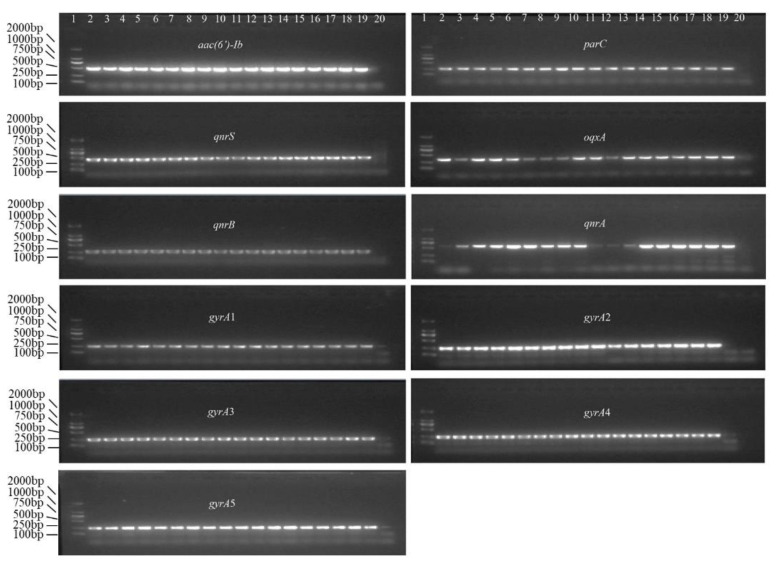
Genetic stability of the target DNA in recombinant strains of in *E. coli* DH5α. In all gels, lane 1, DL 2000 DNA marker; lanes 2–4, amplicons of the target DNA in the original recombinant strains; lanes 5–7, 8–10, 11–13, 14–16, and 17–19, amplicons of the target DNA in the third, sixth, ninth, twelfth, and fifteenth generations of recombinant strains, respectively; and lane 20, double-distilled H_2_O, which served as the blank control.

**Table 1:** PCR primers and annealing temperatures for target DNA detection.

Gene	Primer	Sequence (5′–3′)	Annealing Temperature (°C)	Product Size (bp)	Reference
*qnrA*	*qnrA*-F *qnrA*-R	AGAGGATTTCTCACGCCAGG TGCCAGGCACAGATCTTGAC	56	579	[14]
*qnrB*	*qnrB*-F *qnrB*-R	GGCATTGAAATTCGCCACTG TTTGCTGCTCGCCAGTCGAA	56	263	[14]
*qnrS*	*qnrS*-F *qnrS*-R	GCAAGTTCATTGAACAGGGT TCTAAACCGTCGAGTTCGGCG	56	427	[14]
*oqxA*	*oqxA*-F *oqxA*-R	GACAGCGTCGCACAGAATG GGAGACGAGGTTGGTATGGA	56	339	[15]
*aac(6′)-Ib*	*aac(6′)-Ib*-F *aac(6′)-Ib*-R	TTGCGATGCTCTATGAGTGGCTA CTCGAATGCCTGGCGTGTTT	55	482	[16]
*gyrA*	*gyrA*-F *gyrA*-R	CCGTACCGTCATAGTTATCC CGTTGGTGACGTAATCGGTA	56	251	[17]
*parC*	*parC*-F *parC*-R	TAACAGCAGCTCGGCGTATT CTATGCGATGTCAGAGCTGG	54	262	[18]

**Table 2:** qPCR primers for the target DNA.

Gene	Primer	Sequence (5′–3′)	Product Size (bp)
*qnrA*	*qnrA*-F *qnrA*-R	TGCTTTGGCATAGAGTTCAGG GGCATTGCTCCAGTTGTTTT	192
*qnrB*	*qnrB*-F *qnrB*-R	GGCATTGAAATTCGCCACTG TTTGCTGCTCGCCAGTCGAA	263
*qnrS*	*qnrS*-F *qnrS*-R	TCGTCGCTGCCACTTTGAT ATGCACCCGCTAGGTTCGTT	296
*oqxA*	*oqxA*-F *oqxA*-R	GACAGCGTCGCACAGAATG GGAGACGAGGTTGGTATGGA	339
*aac(6′)-Ib*	*aac(6′)-Ib*-F *aac(6′)-Ib*-R	CCGACACTTGCTGACGTACA GTTTCTTCTTCCCACCATCC	155
*gyrA*	*gyrA*-F *gyrA*-R	CCGTACCGTCATAGTTATCC CGTTGGTGACGTAATCGGTA	251
*parC*	*parC*-F *parC*-R	TAACAGCAGCTCGGCGTATT CTATGCGATGTCAGAGCTGG	262

**Table 3:** Homogeneity parameters of plasmid DNA reference materials.

Gene	Difference	SS	Df	MS	F-Value	*p*-Value	F-Critical Value
*aac(6′)-Ib*	interblock	8.60	11.00	0.78	2.52	0.06	2.72
	intraclass	3.73	12.00	0.31			
*qnrA*	interblock	4.63	11.00	0.42	2.05	0.12	2.72
	intraclass	2.47	12.00	0.21			
*qnrB*	interblock	11.60	11.00	1.05	2.65	0.05	2.72
	intraclass	4.77	12.00	0.40			
*qnrS*	interblock	11.87	11.00	1.08	2.64	0.05	2.72
	intraclass	4.91	12.00	0.41			
*oqxA*	interblock	8.48	11.00	0.77	1.68	0.19	2.72
	intraclass	5.50	12.00	0.46			
*parC*	interblock	1.66	11.00	0.15	0.86	0.60	2.72
	intraclass	2.12	12.00	0.18			
*gyrA*1	interblock	4.31	11.00	0.39	1.18	0.39	2.72
	intraclass	3.98	12.00	0.33			
*gyrA*2	interblock	4.08	11.00	0.37	2.64	0.05	2.72
	intraclass	1.69	12.00	0.14			
*gyrA*3	interblock	6.05	11.00	0.55	1.41	0.28	2.72
	intraclass	4.69	12.00	0.39			
*gyrA*4	interblock	5.59	11.00	0.51	2.23	0.09	2.72
	intraclass	2.74	12.00	0.23			
*gyrA*5	interblock	3.29	11.00	0.30	2.47	0.07	2.72
	intraclass	1.45	12.00	0.12			

Note: “SS” represents stdev square, “Df” represents degree of freedom, and “MS” represents mean square.

**Table 4:** Ct values of qPCR for plasmid DNA reference materials stored at 37 °C (Mean ± SD).

Gene	1 Day	7 Days	13 Days
	Ct Value	Ct Value	Ct Value
*aac(6’)-Ib*	7.33 ± 0.42	8.31 ± 0.57	7.79 ± 0.54
*parC*	7.66 ± 0.58	7.80 ± 0.82	9.24 ± 0.90
*qnrS*	7.26 ± 0.69	6.76 ± 0.25	7.36 ± 0.38
*oqxA*	7.89 ± 0.87	8.39 ± 1.22	7.98 ± 1.55
*qnrB*	8.62 ± 0.15	7.53 ± 0.28	7.75 ±0.61
*qnrA*	8.00 ± 0.63	7.94 ± 0.40	7.85 ± 0.86
*gyrA*1	9.12 ± 1.14	7.13 ± 0.32	8.33 ± 0.23
*gyrA*2	7.55 ± 0.44	8.78 ± 0.80	8.91 ± 1.33
*gyrA*3	8.44 ± 0.51	7.33 ± 0.38	6.39 ± 0.47
*gyrA*4	8.55 ± 0.78	8.16 ± 0.41	8.22 ± 0.85
*gyrA*5	8.42 ± 0.75	7.87 ± 0.77	7.77 ± 0.63

## Supplementary Materials

The following are available online at https://www.mdpi.com/article/10.3390/foods11020154/s1, Supplementary Table S1: Gene information for development of plasmid DNA reference materials; Supplementary Figures S1–S7: Genetic stability of *aac(6′)-Ib*, *parC*, *qnrS*, *oqxA*, *qnrB*, *qnrA*, and *gyrA*, respectively; Supplementary Figure S8: PCR results for homogeneity of plasmid DNA reference materials; Supplementary Figures S9–S15: Homogeneity of *aac(6′)-Ib*, *parC*, *qnrS*, *oqxA*, *qnrA*, *qnrB*, and *gyrA* in plasmid DNA reference materials, respectively; Supplementary Figure S16: PCR results for storage stability of plasmid DNA references materials stored at 37 °C; Supplementary Figures S17–S23: Sequencing results of *aac(6′)-Ib*, *parC*, *qnrS*, *oqxA*, *qnrB*, *qnrA,* and *gyrA* for stability of plasmid DNA references materials stored at 37 °C, respectively; Supplementary Figure S24: PCR results for storage stability of plasmid DNA references materials stored at 4 °C; Supplementary Figures S25–S31: Sequencing results of *aac(6′)-Ib*, *parC*, *qnrS*, *oqxA*, *qnrB*, *qnrA*, and *gyrA* for stability of plasmid DNA references materials stored at 4 °C, respectively; Supplementary Figure S32: PCR results for storage stability of plasmid DNA references materials stored at −20 °C; Supplementary Figures S33–S39: Sequencing results of *aac(6′)-Ib*, *parC*, *qnrS*, *oqxA*, *qnrB*, *qnrA*, and *gyrA* for stability of plasmid DNA references materials stored at −20 °C, respectively (DOCX).
