# Supplementary material for: Developing Qualitative Plasmid DNA Reference Materials to Detect Mechanisms of Quinolone and Fluoroquinolone Resistance in Foodborne Pathogens
Source: Foods. 2022 Jan 7;11(2):154. doi: 10.3390/foods11020154 (PMC8775000; doi:10.3390/foods11020154)
Supplement: Supplementary file 1 [file foods-11-00154-s001.zip › foods-1516153-supplementary.pdf]

Supplementary Information

Table S1. Gene Information for Development of Plasmid DNA Reference Materials.

gene	name	accession number	sequence (5'–3')	product size (bp)	amino acid mutation
aac(6')-Ib	*aac(6')-Ib*	MN045292	TTGCGATGCTCTATGAGTGGCTAAATCGATCTCATATCGTCGAGTGGTGGGGCGGAGA<br>AGAAGCACGCCCGACACTTGCTGACGTACAGGAACAGTACTTGCCAAGCGTTTTAGC<br>GCAAGAGTCCGTCACTCCATACATTGCAATGCTGAATGGAGAGCCGATTGGGTATGCC<br>CAGTCGTACGTTGCTCTTGGAAGCGGGGACGGATGGTGGGAAGAAGAAACCGATCCA<br>GGAGTACGCGGAATAGACCAGTTACTGGCGAATGCATCACAACTGGGCAAAGGCTTG<br>GGAACCAAGCTGGTTCGAGCTCTGGTTGAGTTGCTGTTCAATGATCCCGAGGTCACCA<br>AGATCCAAACGGACCCGTCGCCGAGCAACTTGCGAGCGATCCGATGCTACGAGAAAG<br>CGGGGTTTGAGAGGCAAGGTACCGTAACCACCCCAGATGGTCCAGCCGTGTACATGG<br>TTCAAACACGCCAGGCATTCGAG	482	–
qnrA	*qnrA*	MN045282	AGAGGATTTCTCACGCCAGGATTTGAGTGACAGCCGTTTTCGCCGCTGCCGCTTTTATC<br>AGTGTGACTTCAGCCACTGTCAGCTGCAGGATGCCAGTTTCGAGGATTGCAGTTTCAT<br>TGAAAGCGGCGCCGTTGAAGGGTGTCACTTCAGCTATGCCGATCTGCGCGATGCCAGT<br>TTCAAGGCCTGCCGTCTGTCTTTGGCCAACTTCAGCGGTGCCAACTGCTTTGGCATAG<br>AGTTCAGGGAGTGCGATCTCAAGGGCGCCAACTTTTCCCGGGCCCGCTTCTACAATCA<br>AGTCAGCCATAAGATGTACTTCTGCTCGGCTTATATCTCAGGTTGCAACCTGGCCTATA<br>CCAACTTGAGTGGCCAATGCCTGGAAAAATGCGAGCTGTTTGAAAACAACTGGAGCA<br>ATGCCAATCTCAGCGGCGCTTCCTTGATGGGCTCAGATCTCAGCCGCGGCACCTTCTC<br>CCGCGACTGTTGGCAACAGGTCAATCTGCGGGGCTGTGGCCTAACCTTTGCCGATCTG<br>GATGGGCTCGACCCCAGACGGGTCAACCTCGAAGGAGTCAAGATCTGTGCCTGGCA	579	–
qnrB	*qnrB*	MN045281	GGCATTGAAATTCGCCACTGCCGCGCACAAGGCGCAGATTTCCGCGGTGCAAGCTTTA<br>TGAATATGATCACCACGCGCACCTGGTTTTGCAGCGCATATATCACTAATACTAATCTAA	263	–

			GCTACGCCAATTTTTCGAAAGTCGTGTTGGAAAAGTGTGAGCTGTGGGAAAACCGCT<br>GGATGGGGACTCAGGTACTGGGTGCGACGTTCAGTGGTTCAGATCTCTCCGGCGGCG<br>AGTTTTCGACTTTCGACTGGCGAGCAGCAAA		
qnrS	*qnrS*	MN045280	GCAAGTTCATTGAACAGGGTGATATTCGTCGCTGCCACTTTGATGTCGCAGATCTTCGT<br>GATGCAAGTTTCCAACAATGCCAACTTGCGATGGCAAACTTCAGTAATGCCAATTGCT<br>ACGGTATAGAGTTCCGTGCGTGTGATTTAAAAGGTGCCAACTTTTCCCGAACAAACTT<br>TGCCCATCAAGTGAGTAATCGTATGTACTTTTGCTCAGCATTTATTTCTGGATGTAATCT<br>TTCCTATGCCAATATGGAGAGGGTTTGTTTAGAAAAATGTGAGTTGTTTGAAAATCGCT<br>GGATAGGAACGAACCTAGCGGGTGCATCACTGAAAGAGTCAGACTTAAGTCGAGGTG<br>TTTTTTCCGAAGATGTCTGGGGGCAATTTAGCCTACAGGGGGCCAATTTATGCCACGCC<br>GAACTCGACGGTTTAGA	427	–
oqxA	*oqxA*	MN045291	GACAGCGTCGCACAGAATGCTGCGCCTCCCGCCCCGACGGTCAGCGCCGCTAAGGTG<br>CTGGTGAAGTCGATCAGTCAGTGGGATAGTTTTAACGGTCGCATTGAAGCGGTGGAGA<br>GCGTTCAGCTTCGCCCTCGCGTCTCGGGATACATTGATAAAGTGAATTACACTGACGG<br>CCAGGAGGTGAAAAAGGGCCAGGTGCTGTTCACGATAGATGACAGAACCTATCGCGC<br>CGCGCTGGAGCAGGCGCAGGCGGCGTTGGCAAGAGCCAAAACGCAGGCCAGCCTCG<br>CGCAAAGCGAGGCGAACCGCACCGATAAATTAGTCCATACCAACCTCGTCTCC	339	–
parC	*parC*	MN045289	CTATGCGATGTCAGAGCTGGGGCTGAACGCCACCGCTAAATTTAAAAAATCCGCCCGT<br>ACCGTTGGTGACGTACTGGGTAAGTATCACCCGCATGGCGACCGCGCCTGCTATGAAG<br>CCATGGTGCTGATGGCGCAGCCGTTCTCTTACCGTTACCCGCTGGTCGATGGCCAGGG<br>GAACTGGGGCGCGCCGGATGATCCGAAGTCATTCGCGGCGATGCGTTATACCGAATCC<br>CGCCTGTCCAAATACGCCGAGCTGCTGTTA	262	Ser80Arg
gyrA	*gyrA*1	MN045284	CGTTGGTGACGTAATCGGTAAATACCATCCCCACGGCGATTCCGCAGTGTATAACACCA<br>TCGTTCGTATGGCGCAGCCATTCTCGCTGCGTTACATGCTGGTGGATGGTCAGGGTAAC<br>TTCGGTTCTATTGACGGCGACTCCGCGGCGGCAATGCGTTATACGGAGATCCGTCTGG	251	Asp87Asn

		CGAAAATCGCCCACGAACTGATGGCCGATCTCGAAAAAGAGACGGTGGATTTCGTGG ATAACTATGACGGTACGG		
*gyrA*2	MN045285	CGTTGGTGACGTAATCGGTAAATACCATCCCCACGGCGATTACGCAGTGTATGACACCA TCGTTCGTATGGCGCAGCCATTCTCGCTGCGTTACATGCTGGTGGATGGTCAGGGTAAC TTCGGTTCTATTGACGGCGACTCCGCGGCGGCAATGCGTTATACGGAGATCCGTCTGG CGAAAATCGCCCACGAACTGATGGCCGATCTCGAAAAAGAGACGGTGGATTTCGTGG ATAACTATGACGGTACGG	251	Ser83Tyr
*gyrA*3	MN045286	CGTTGGTGACGTAATCGGTAAATACCATCCCCACGGCGATTTCGCAGTGTATGACACC ATCGTTCGTATGGCGCAGCCATTCTCGCTGCGTTACATGCTGGTGGATGGTCAGGGTAA CTTCGGTTCTATTGACGGCGACTCCGCGGCGGCAATGCGTTATACGGAGATCCGTCTG GCGAAAATCGCCCACGAACTGATGGCCGATCTCGAAAAAGAGACGGTGGATTTCGTG GATAACTATGACGGTACGG	251	Ser83Phe
*gyrA*4	MN045287	CGTTGGTGACGTAATCGGTAAATACCATCCCCACGGCGATTTCGCAGTGTATGGCACCA TCGTTCGTATGGCGCAGCCATTCTCGCTGCGTTACATGCTGGTGGATGGTCAGGGTAAC TTCGGTTCTATTGACGGCGACTCCGCGGCGGCAATGCGTTATACGGAGATCCGTCTGG CGAAAATCGCCCACGAACTGATGGCCGATCTCGAAAAAGAGACGGTGGATTTCGTGG ATAACTATGACGGTACGG	251	Ser83Phe Asp87Gly
*gyrA*5	MN045288	CGTTGGTGACGTAATCGGTAAATACCATCCCCACGGCGATTTCGCAGTGTATGCCACCA TCGTTCGTATGGCGCAGCCATTCTCGCTGCGTTACATGCTGGTGGATGGTCAGGGTAAC TTCGGTTCTATTGACGGCGACTCCGCGGCGGCAATGCGTTATACGGAGATCCGTCTGG CGAAAATCGCCCACGAACTGATGGCCGATCTCGAAAAAGAGACGGTGGATTTCGTGG ATAACTATGACGGTACGG	251	Ser83Phe Asp87Ala

Note: “–” means no artificial mutation.

Figure S1. Genetic stability of *aac(6')-Ib*.

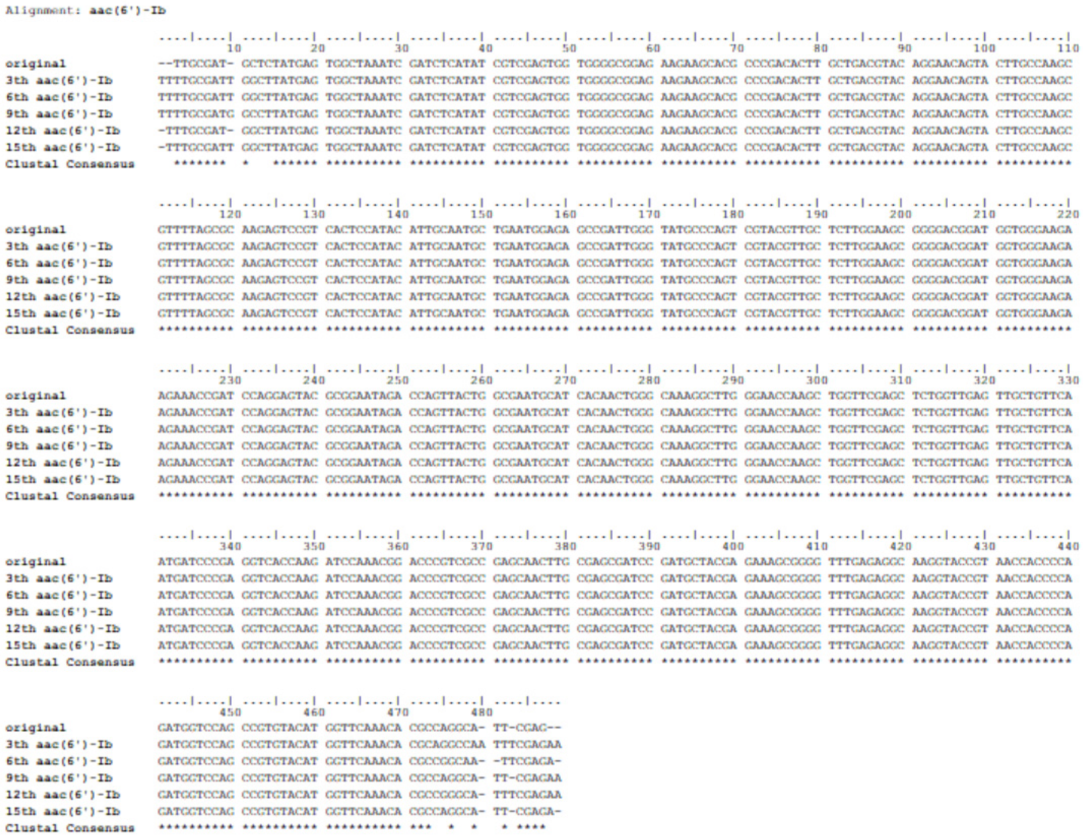

Note: "*" means no difference in nucleotide. Due to the instability of Sanger sequencing, it is common that there are differences in the first and last 20 nucleotides.

Figure S2. Genetic stability of *parC*.

```
Alignment: parC

                   ....|....| ....|....| ....|....| ....|....| ....|....| ....|....| ....|....| ....|....| ....|....| ..
                           10         20         30         40         50         60         70         80         90
original           -YAMSELGLN ATAKFKKSAR TVGDVLGKYH PHGDRACYEA MVLMAQPFSY RYPLVDGQGN WGAPDDPKSF AAMRYTESRL SKYAELLL-- --
3th parC           -YAMSELGLN ATAKFKKSAR TVGDVLGKYH PHGDRACYEA MVLMAQPFSY RYPLVDGQGN WGAPDDPKSF AAMRYTESRL SKYAELLL-- --
6th parC           -LCDVKLGLN ATAKFKKSAR TVGDVLGKYH PHGDRACYEA MVLMAQPFSY RYPLVDGQGN WGAPDDPKSF AAMRYTESRL SKYAGLLL-- --
9th parC           -LCDVKLGLN ATAKFKKSAR TVGDVLGKYH PHGDRACYEA MVLMAQPFSY RYPLVDGQGN WGAPDDPKSF AAMRYTESRL SKYAGLLL-- --
12th parC          FYAMSELGLN ATAKFKKSAR TVGDVLGKYH PHGDRACYEA MVLMAQPFSY RYPLVDGQGN WGAPDDPKSF AAMRYTESRL SKYAELLLNS KI
15th parC          -YAMSELGLN ATAKFKKSAR TVGDVLGKYH PHGDRACYEA MVLMAQPFSY RYPLVDGQGN WGAPDDPKSF AAMRYTESRL SKYAEPAVKQ --
Clustal Consensus   .  :**** ********** ********** ********** ********** ********** ********** ********** ****    :
```

Note: “*” means no difference in amino acid. Due to the instability of Sanger sequencing, it is common that there are differences in the first and last 10 amino acids.

Figure S3. Genetic stability of *qnrS*.

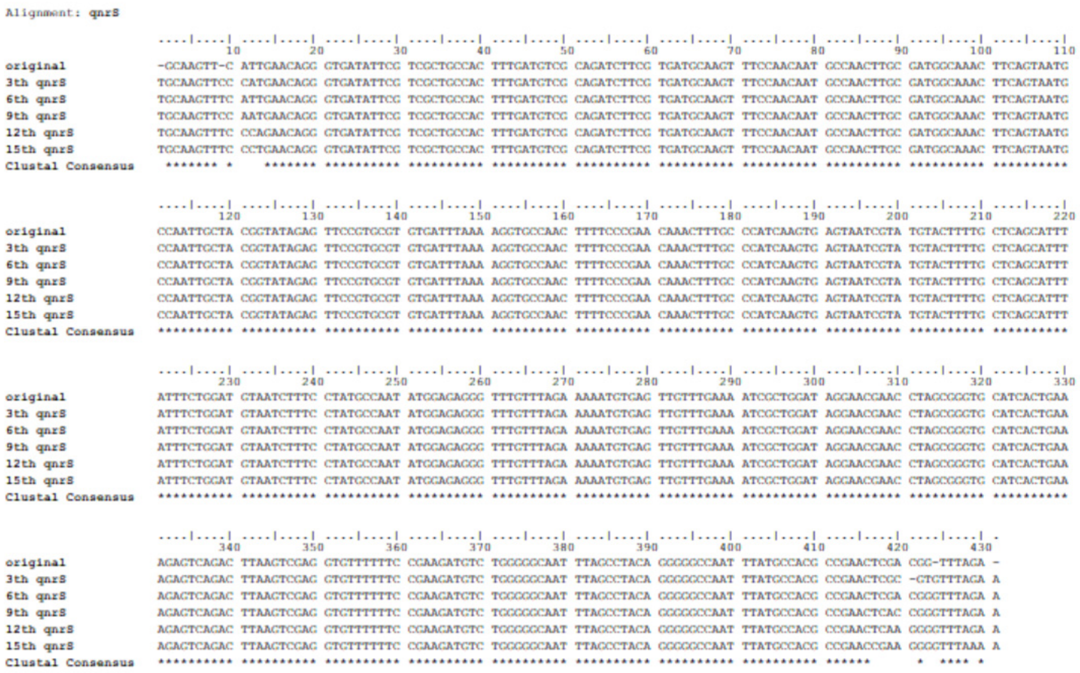

Note: “*” means no difference in nucleotide. Due to the instability of Sanger sequencing, it is common that there are differences in the first and last 20 nucleotides.

Figure S4. Genetic stability of *oqxA*.

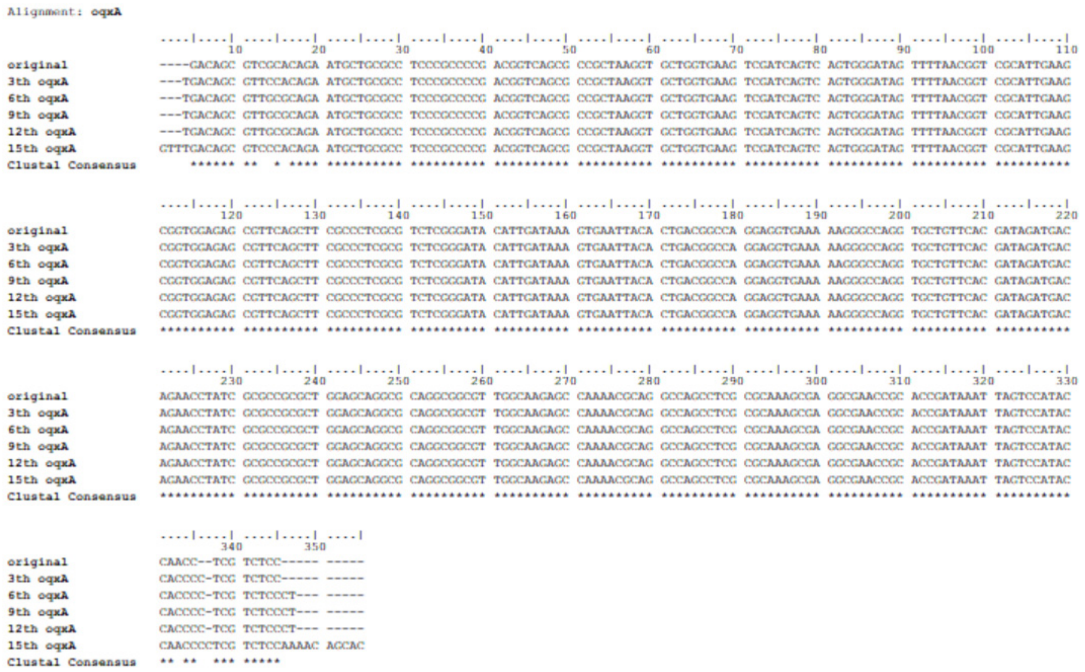

Note: "*" means no difference in nucleotide. Due to the instability of Sanger sequencing, it is common that there are differences in the first and last 20 nucleotides.

Figure S5. Genetic stability of *qnrB*.

```
Alignment: qnrB

                    ....|....| ....|....| ....|....| ....|....| ....|....| ....|....| ....|....| ....|....| ....|....| ....|....| ....|....|
                           10         20         30         40         50         60         70         80         90        100        110
original            -GGCATTGAA ATTCGCCACT GCCGCGCACA AGGCGCAGAT TTCCGCGGTG CAAGCTTTAT GAATATGATC ACCACGCGCA CCTGGTTTTG CAGCGCATAT ATCACTAATA
3th qnrB            TGGCATTGAA ATTCGCCACT GCCGCGCACA AGGCGCAGAT TTCCGCGGTG CAAGCTTTAT GAATATGATC ACCACGCGCA CCTGGTTTTG CAGCGCATAT ATCACTAATA
6th qnrB            TGGCATTGAA ATTCGCCACT GCCGCGCACA AGGCGCAGAT TTCCGCGGTG CAAGCTTTAT GAATATGATC ACCACGCGCA CCTGGTTTTG CAGCGCATAT ATCACTAATA
9th qnrB            TGGCATTGAA ATTCGCCACT GCCGCGCACA AGGCGCAGAT TTCCGCGGTG CAAGCTTTAT GAATATGATC ACCACGCGCA CCTGGTTTTG CAGCGCATAT ATCACTAATA
12th qnrB           TGGCATTGAA ATTCGCCACT GCCGCGCACA AGGCGCAGAT TTCCGCGGTG CAAGCTTTAT GAATATGATC ACCACGCGCA CCTGGTTTTG CAGCGCATAT ATCACTAATA
15th qnrB           TGGCATTGAA ATTCGCCACT GCCGCGCACA AGGCGCAGAT TTCCGCGGTG CAAGCTTTAT GAATATGATC ACCACGCGCA CCTGGTTTTG CAGCGCATAT ATCACTAATA
Clustal Consensus    ********* ********** ********** ********** ********** ********** ********** ********** ********** ********** **********

                    ....|....| ....|....| ....|....| ....|....| ....|....| ....|....| ....|....| ....|....| ....|....| ....|....| ....|....|
                          120        130        140        150        160        170        180        190        200        210        220
original            CTAATCTAAG CTACGCCAAT TTTTCGAAAG TCGTGTTGGA AAAGTGTGAG CTGTGGGAAA ACCGCTGGAT GGGGACTCAG GTACTGGGTG CGACGTTCAG TGGTTCAGAT
3th qnrB            CTAATCTAAG CTACGCCAAT TTTTCGAAAG TCGTGTTGGA AAAGTGTGAG CTGTGGGAAA ACCGCTGGAT GGGGACTCAG GTACTGGGTG CGACGTTCAG TGGTTCAGAT
6th qnrB            CTAATCTAAG CTACGCCAAT TTTTCGAAAG TCGTGTTGGA AAAGTGTGAG CTGTGGGAAA ACCGCTGGAT GGGGACTCAG GTACTGGGTG CGACGTTCAG TGGTTCAGAT
9th qnrB            CTAATCTAAG CTACGCCAAT TTTTCGAAAG TCGTGTTGGA AAAGTGTGAG CTGTGGGAAA ACCGCTGGAT GGGGACTCAG GTACTGGGTG CGACGTTCAG TGGTTCAGAT
12th qnrB           CTAATCTAAG CTACGCCAAT TTTTCGAAAG TCGTGTTGGA AAAGTGTGAG CTGTGGGAAA ACCGCTGGAT GGGGACTCAG GTACTGGGTG CGACGTTCAG TGGTTCAGAT
15th qnrB           CTAATCTAAG CTACGCCAAT TTTTCGAAAG TCGTGTTGGA AAAGTGTGAG CTGTGGGAAA ACCGCTGGAT GGGGACTCAG GTACTGGGTG CGACGTTCAG TGGTTCAGAT
Clustal Consensus   ********** ********** ********** ********** ********** ********** ********** ********** ********** ********** **********

                    ....|....| ....|....| ....|....| ....|....| ....|
                          230        240        250        260
original            CTCTCCGGCG GCGAGTTTTC GACTTTCGAC TGGCGAGCAG CAAA-
3th qnrB            CTCTCCGGCG GCGAGTTTTC GACTTTCGAC TGGCGAACAG CAAAA
6th qnrB            CTCTCCGGCG GCGAGTTTTC GACTTTCGAC TGGCGAACAG CAAAA
9th qnrB            CTCTCCGGCG GCGAGTTTTC GACTTTCGAC TGGCGAACAG CAAAA
12th qnrB           CTCTCCGGCG GCGAGTTTTC GACTTTCGAC TGGCGAACGG CAAAA
15th qnrB           CTCTCCGGCG GCGAGTTTTC GACTTTCGAC TGGCGAACGG CAAAA
Clustal Consensus   ********** ********** ********** ****** * * ****
```

Note: “*” means no difference in nucleotide. Due to the instability of Sanger sequencing, it is common that there are differences in the first and last 20 nucleotides.

Figure S6. Genetic stability of *qnrA*.

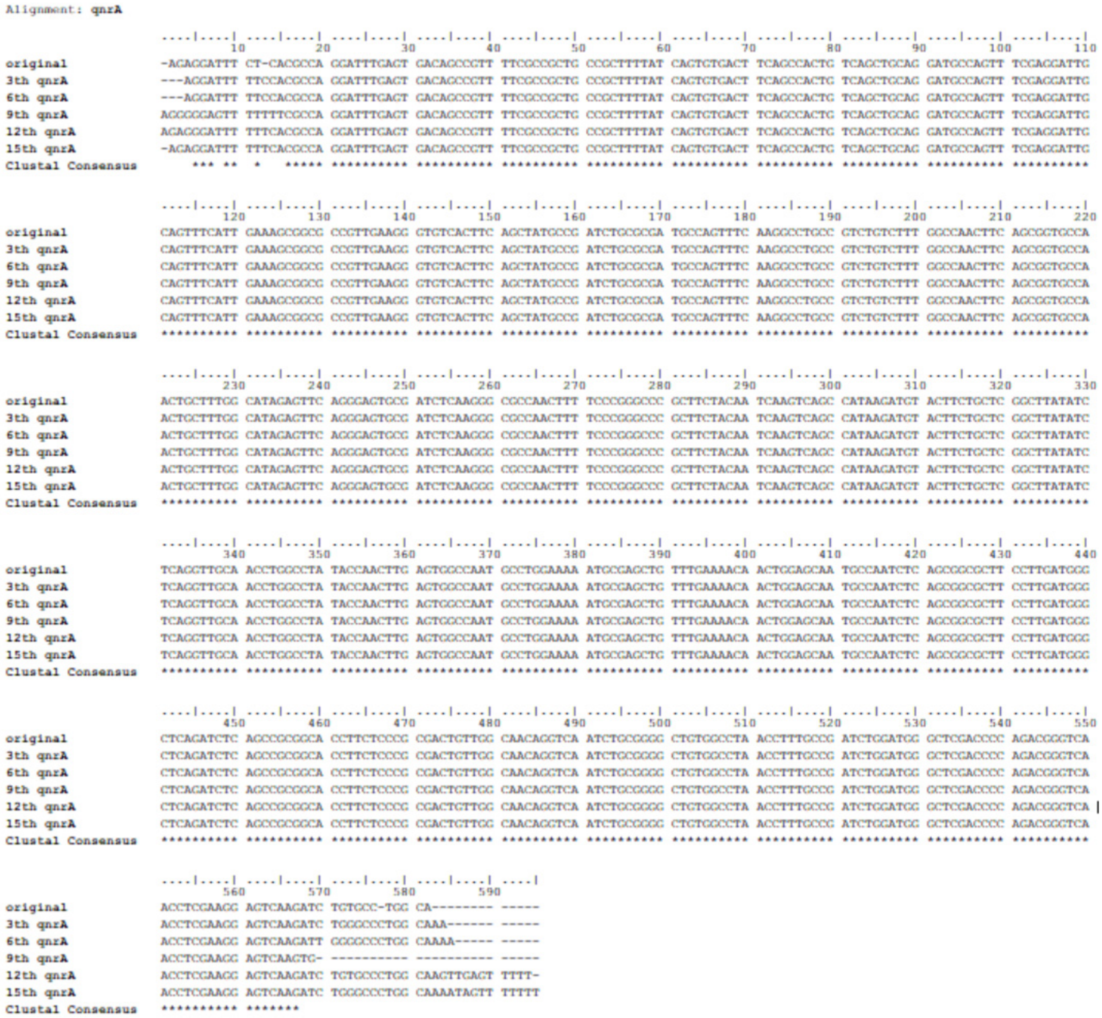

Note: "*" means no difference in nucleotide. Due to the instability of Sanger sequencing, it is common that there are differences in the first and last 20 nucleotides.

Figure S7. Genetic stability of *gyrA*.

```
Alignment:gyrA1

                    ....|....| ....|....| ....|....| ....|....| ....|....| ....|....| ....|....| ....|....| ....|
                            10         20         30         40         50         60         70         80
original            -VGDVIGKYH PHGDSAVYNT IVRMAQPFSL RYMLVDGQGN FGSIDGDSAA AMRYTEIRLA KIAHELMADL EKETVDFVDN YDGT-
3th gyrA1           FVGDVIGKYH PHGDSAVYNT IVRMAQPFSL RYMLVDGQGN FGSIDGDSAA AMRYTEIRLA KIAHELMADL EKETVDFVDN YDGTE
6th gyrA1           -VGDVIGKYH PHGDSAVYNT IVRMAQPFSL RYMLVDGQGN FGSIDGDSAA AMRYTEIRLA KIAHELMADL EKETVDFVDN YDGTE
9th gyrA1           FVGDVIGKYH PHGDSAVYNT IVRMAQPFSL RYMLVDGQGN FGSIDGDSAA AMRYTEIRLA KIAHELMADL EKETVDFVDN YDGTE
12th gyrA1          FVGDVIGKYH PHGDSAVYNT IVRMAQPFSL RYMLVDGQGN FGSIDGDSAA AMRYTEIRLA KIAHELMADL EKETVDFVDN YDGT-
15th gyrA1          -VGDVIGKYH PHGDSAVYNT IVRMAQPFSL RYMLVDGQGN FGSIDGDSAA AMRYTEIRLA KIAHELMADL EKETVDFVDN YDGT-
Clustal Consensus    ********* ********** ********** ********** ********** ********** ********** ********** ****

Alignment: gyrA2

                    ....|....| ....|....| ....|....| ....|....| ....|....| ....|....| ....|....| ....|....| ....|
                            10         20         30         40         50         60         70         80
original            -VGDVIGKYH PHGDYAVYDT IVRMAQPFSL RYMLVDGQGN FGSIDGDSAA AMRYTEIRLA KIAHELMADL EKETVDFVDN YDGT-
3th gyrA2           -VGDVIGKYH PHGDYAVYDT IVRMAQPFSL RYMLVDGQGN FGSIDGDSAA AMRYTEIRLA KIAHELMADL EKETVDFVDN YDGT-
6th gyrA2           -VGDVIGKYH PHGDYAVYDT IVRMAQPFSL RYMLVDGQGN FGSIDGDSAA AMRYTEIRLA KIAHELMADL EKETVDFVDN YDGT-
9th gyrA2           -VGDVIGKYH PHGDYAVYDT IVRMAQPFSL RYMLVDGQGN FGSIDGDSAA AMRYTEIRLA KIAHELMADL EKETVDFVDN YDGT-
12th gyrA2          -VGDVIGKYH PHGDYAVYDT IVRMAQPFSL RYMLVDGQGN FGSIDGDSAA AMRYTEIRLA KIAHELMADL EKETVDFVDN YDGTG
15th gyrA2          IVGDVIGKYH PHGDYAVYDT IVRMAQPFSL RYMLVDGQGN FGSIDGDSAA AMRYTEIRLA KIAHELMADL EKETVDFVDN YDGTG
Clustal Consensus    ********* ********** ********** ********** ********** ********** ********** ********** ****

Alignment: gyrA3

                    ....|....| ....|....| ....|....| ....|....| ....|....| ....|....| ....|....| ....|....| ....|
                            10         20         30         40         50         60         70         80
original            -VGDVIGKYH PHGDFAVYDT IVRMAQPFSL RYMLVDGQGN FGSIDGDSAA AMRYTEIRLA KIAHELMADL EKETVDFVDN YDGT-
3th gyrA3           SLVTVIGKYH PHGDFAVYDT IVRMAQPFSL RYMLVDGQGN FGSIDGDSAA AMRYTEIRLA KIAHELMADL EKETVDFVDN YDGT-
6th gyrA3           SLVIVIGKYH PHGDFAVYDT IVRMAQPFSL RYMLVDGQGN FGSIDGDSAA AMRYTEIRLA KIAHELMADL EKETVDFVDN YERYG
9th gyrA3           -VGDVIGKYH PHGDFAVYDT IVRMAQPFSL RYMLVDGQGN FGSIDGDSAA AMRYTEIRLA KIAHELMADL EKETVDFVDN YDGT-
12th gyrA3          -VGDVIGKYH PHGDFAVYDT IVRMAQPFSL RYMLVDGQGN FGSIDGDSAA AMRYTEIRLA KIAHELMADL EKETVDFVDN YDGTE
15th gyrA3          SLVSVIGKYH PHGDFAVYDT IVRMAQPFSL RYMLVDGQGN FGSIDGDSAA AMRYTEIRLA KIAHELMADL EKETVDFVDN YDGTE
Clustal Consensus    :   ****** ********** ********** ********** ********** ********** ********** ********** *:

Alignment: gyrA4

                    ....|....| ....|....| ....|....| ....|....| ....|....| ....|....| ....|....| ....|....| ....
                            10         20         30         40         50         60         70         80
original            -VGDVIGKYH PHGDFAVYGT IVRMAQPFSL RYMLVDGQGN FGSIDGDSAA AMRYTEIRLA KIAHELMADL EKETVDFVDN YDGT
3th gyrA4           LVGDVIGKYH PHGDFAVYGT IVRMAQPFSL RYMLVDGQGN FGSIDGDSAA AMRYTEIRLA KIAHELMADL EKETVDFVDN YDGT
6th gyrA4           -VGDVIGKYH PHGDFAVYGT IVRMAQPFSL RYMLVDGQGN FGSIDGDSAA AMRYTEIRLA KIAHELMADL EKETVDFVDN YDGT
9th gyrA4           -VGDVIGKYH PHGDFAVYGT IVRMAQPFSL RYMLVDGQGN FGSIDGDSAA AMRYTEIRLA KIAHELMADL EKETVDFVDN YDGT
12th gyrA4          IVGDVIGKYH PHGDFAVYGT IVRMAQPFSL RYMLVDGQGN FGSIDGDSAA AMRYTEIRLA KIAHELMADL EKETVDFVDN YDGT
15th gyrA4          LVGDVIGKYH PHGDFAVYGT IVRMAQPFSL RYMLVDGQGN FGSIDGDSAA AMRYTEIRLA KIAHELMADL EKETVDFVDN YDGT
Clustal Consensus    ********* ********** ********** ********** ********** ********** ********** ********** ****

Alignment: gyrA5

                    ....|....| ....|....| ....|....| ....|....| ....|....| ....|....| ....|....| ....|....| ....|
                            10         20         30         40         50         60         70         80
original            -VGDVIGKYH PHGDFAVYAT IVRMAQPFSL RYMLVDGQGN FGSIDGDSAA AMRYTEIRLA KIAHELMADL EKETVDFVDN YDGT-
3th gyrA5           SLVTVIGKYH PHGDFAVYAT IVRMAQPFSL RYMLVDGQGN FGSIDGDSAA AMRYTEIRLA KIAHELMADL EKETVDFVDN YDGT-
6th gyrA5           SLVTVIGKYH PHGDFAVYAT IVRMAQPFSL RYMLVDGQGN FGSIDGDSAA AMRYTEIRLA KIAHELMADL EKETVDFVDN YDGT-
9th gyrA5           -VGDRIGKYH PHGDFAVYAT IVRMAQPFSL RYMLVDGQGN FGSIDGDSAA AMRYTEIRLA KIAHELMADL EKETVDFVDN YARYG
12th gyrA5          -VGDLIGKYH PHGDFAVYAT IVRMAQPFSL RYMLVDGQGN FGSIDGDSAA AMRYTEIRLA KIAHELMADL EKETVDFVDN YGRYG
15th gyrA5          -VGDVIGKYH PHGDFAVYAT IVRMAQPFSL RYMLVDGQGN FGSIDGDSAA AMRYTEIRLA KIAHELMADL EKETVDFVDN YGRYG
Clustal Consensus    :    ***** ********** ********** ********** ********** ********** ********** ********** *
```

Note: “*” means no difference in amino acid. Due to the instability of Sanger sequencing, it is common that there are differences in the first and last 10 amino acids.

Figure S8. PCR results for homogeneity of plasmid DNA reference materials.

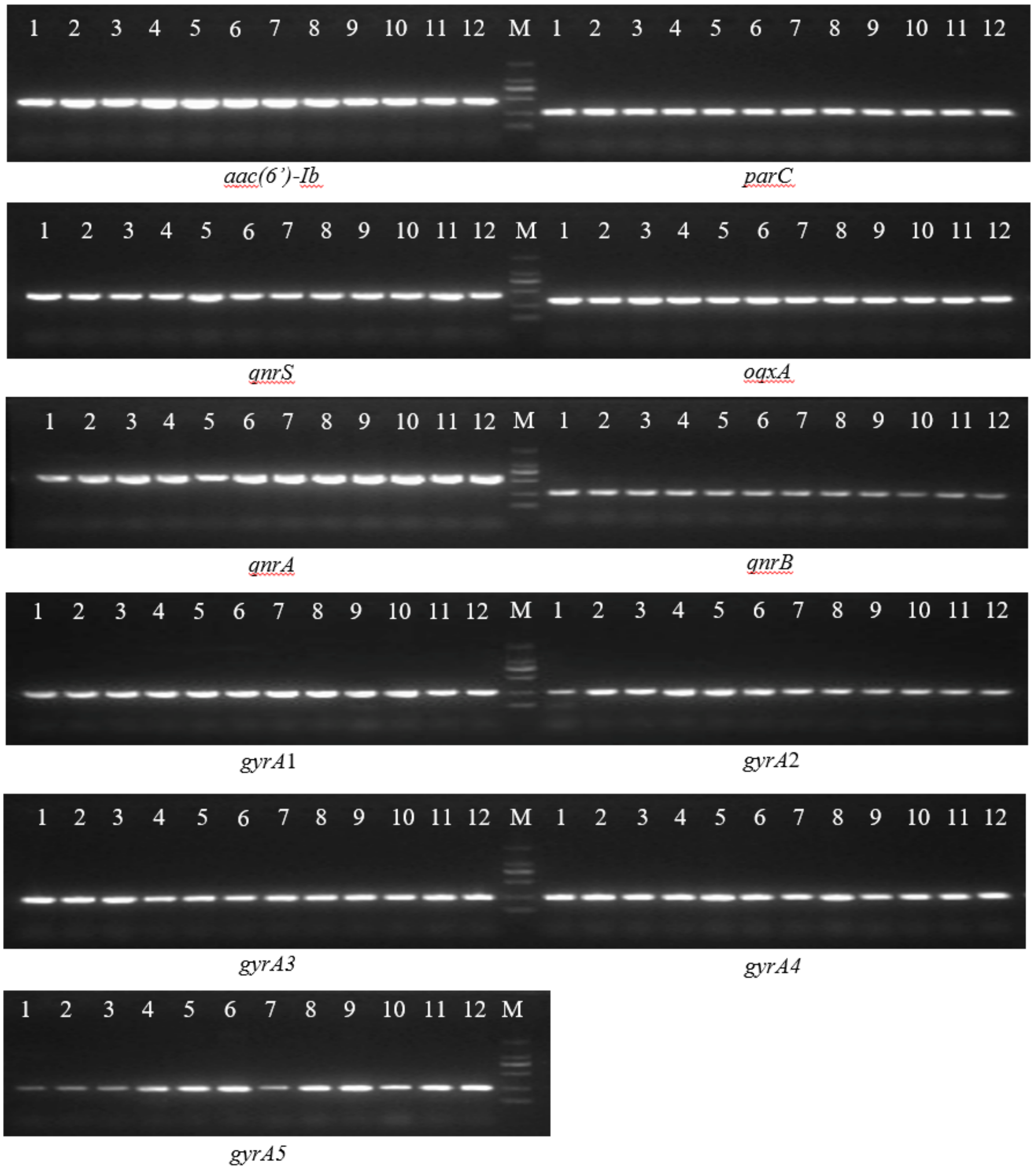

Note: Lane M is DL 2000 DNA marker, lanes 1–12 are PCR bands of each reference material in 12 tubes.

Figure S9. Homogeneity of *aac(6’)-Ib* in plasmid DNA reference materials.

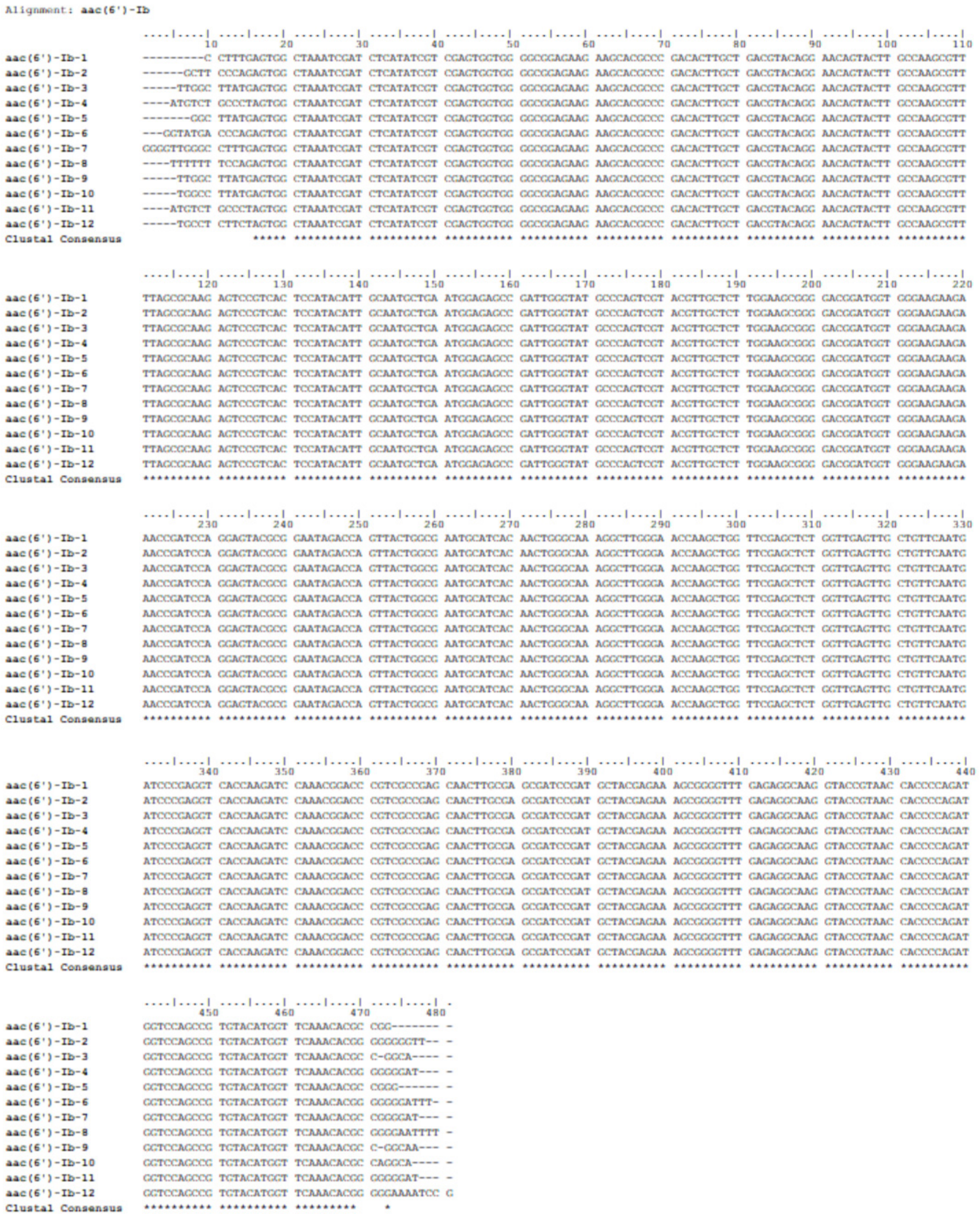

Note: “*” means no difference in nucleotide. Due to the instability of Sanger sequencing, it is common that there are differences in the first and last 20 nucleotides.

Figure S10. Homogeneity of *parC* in plasmid DNA reference materials.

```
Alignment: parC

                    ....|....| ....|....| ....|....| ....|....| ....|....| ....|....| ....|....| ....|....| ....|....
                            10         20         30         40         50         60         70         80
parC-1              -YAMSELGLN ATAKFKKSAR TVGDVLGKYH PHGDRACYEA MVLMAQPFSY RYPLVDGQGN WGAPDDPKSF AAMRYTESRL SKYAELLL-
parC-2              SMR-FELGLN ATAKFKKSAR TVGDVLGKYH PHGDRACYEA MVLMAQPFSY RYPLVDGQGN WGAPDDPKSF AAMRYTESRL SKYAKSAV-
parC-3              SMRFVKLGLN ATAKFKKSAR TVGDVLGKYH PHGDRACYEA MVLMAQPFSY RYPLVDGQGN WGAPDDPKSF AAMRYTESRL SKYAGLLL-
parC-4              -YAMSELGLN ATAKFKKSAR TVGDVLGKYH PHGDRACYEA MVLMAQPFSY RYPLVDGQGN WGAPDDPKSF AAMRYTESRL SKYAEAAV-
parC-5              SMRFVKLGLN ATAKFKKSAR TVGDVLGKYH PHGDRACYEA MVLMAQPFSY RYPLVDGQGN WGAPDDPKSF AAMRYTESRL SKYAGLLL-
parC-6              -YAMSELGLN ATAKFKKSAR TVGDVLGKYH PHGDRACYEA MVLMAQPFSY RYPLVDGQGN WGAPDDPKSF AAMRYTESRL SKYAEPAV-
parC-7              SMRLLKLGLN ATAKFKKSAR TVGDVLGKYH PHGDRACYEA MVLMAQPFSY RYPLVDGQGN WGAPDDPKSF AAMRYTESRL SKYGGLLF-
parC-8              FYAMVELGLN ATAKFKKSAR TVGDVLGKYH PHGDRACYEA MVLMAQPFSY RYPLVDGQGN WGAPDDPKSF AAMRYTESRL SKYAGVCCL
parC-9              SMRFVKLGLN ATAKFKKSAR TVGDVLGKYH PHGDRACYEA MVLMAQPFSY RYPLVDGQGN WGAPDDPKSF AAMRYTESRL SKYAGLLL-
parC-10             SMRLVELGLN ATAKFKKSAR TVGDVLGKYH PHGDRACYEA MVLMAQPFSY RYPLVDGQGN WGAPDDPKSF AAMRYTESRL SKYAGPAV-
parC-11             -YAMSELGLN ATAKFKKSAR TVGDVLGKYH PHGDRACYEA MVLMAQPFSY RYPLVDGQGN WGAPDDPKSF AAMRYTESRL SKYAELLL-
parC-12             SMRFVKLGLN ATAKFKKSAR TVGDVLGKYH PHGDRACYEA MVLMAQPFSY RYPLVDGQGN WGAPDDPKSF AAMRYTESRL SKYAGLLL-
Clustal Consensus        :**** ********** ********** ********** ********** ********** ********** ********** ***.
```

Note: “*” means no difference in amino acid. Due to the instability of Sanger sequencing, it is common that there are differences in the first and last 10 amino acids.

Figure S11. Homogeneity of *qnrS* in plasmid DNA reference materials.

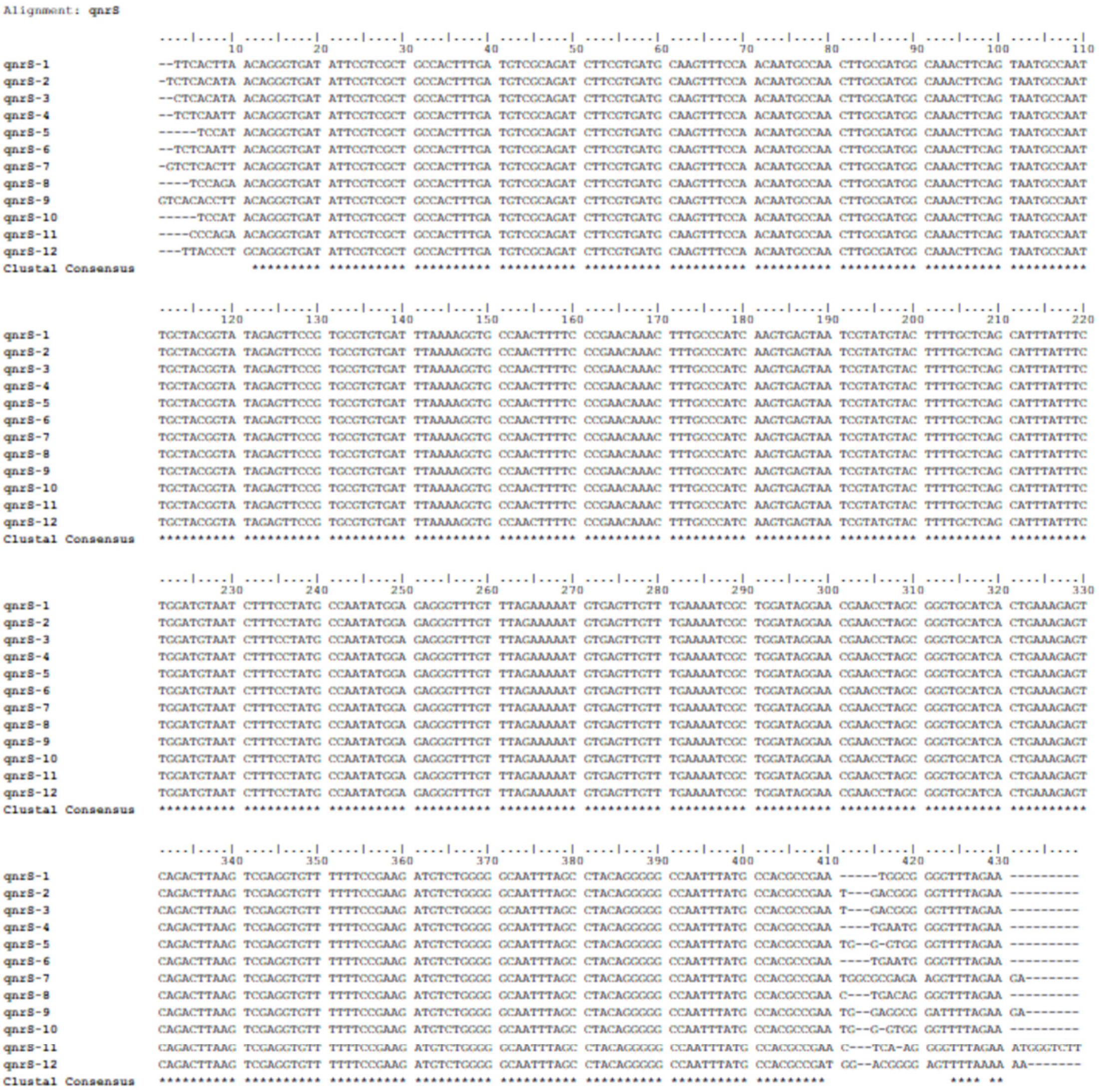

Note: "*" means no difference in nucleotide. Due to the instability of Sanger sequencing, it is common that there are differences in the first and last 20 nucleotides.

Figure S12. Homogeneity of *oqxA* in plasmid DNA reference materials.

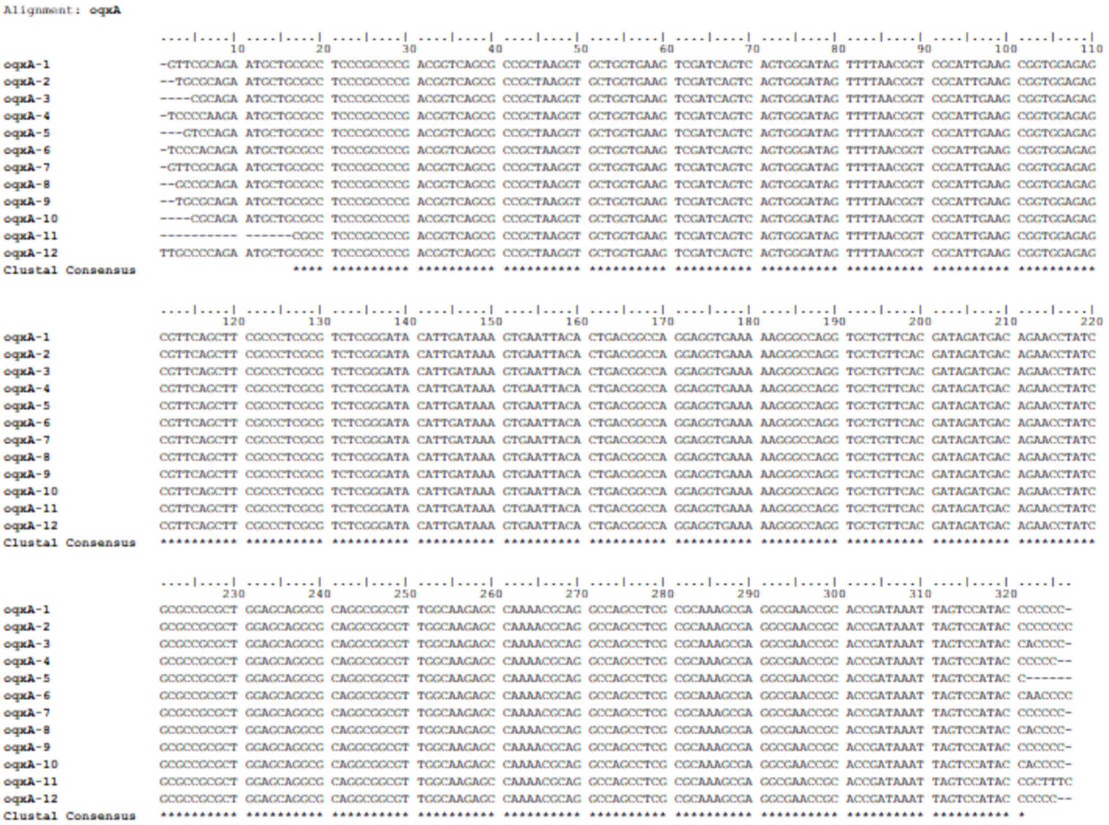

Note: “*” means no difference in nucleotide. Due to the instability of Sanger sequencing, it is common that there are differences in the first and last 20 nucleotides.

Figure S13. Homogeneity of *qnrA* in plasmid DNA reference materials.

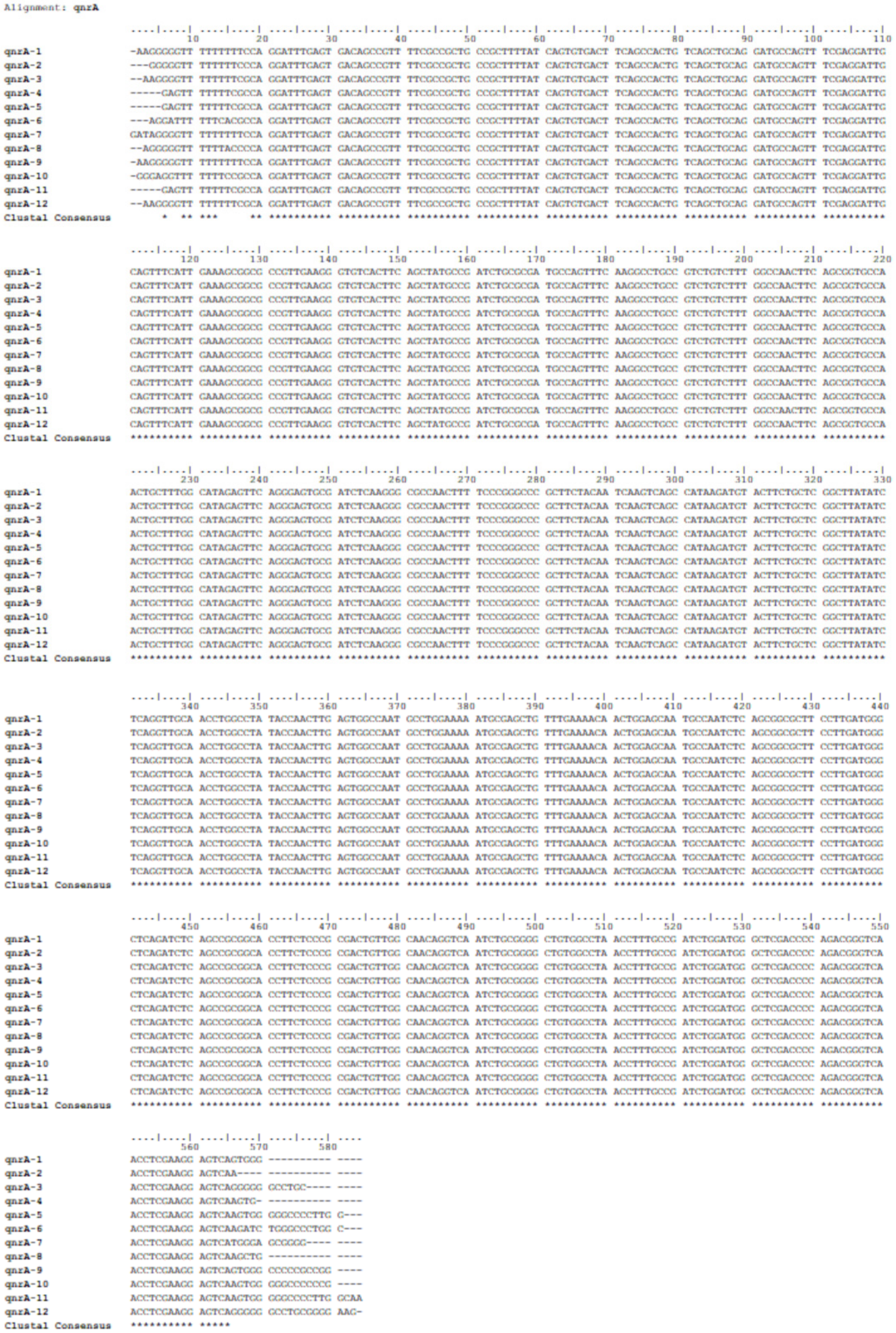

Note: “*” means no difference in nucleotide. Due to the instability of Sanger sequencing, it is common that there are differences in the first and last 20 nucleotides.

Figure S14. Homogeneity of *qnrB* in plasmid DNA reference materials.

```
Alignment: qnrB

                   ....|....| ....|....| ....|....| ....|....| ....|....| ....|....| ....|....| ....|....| ....|....| ....|....| ....|....|
                          10         20         30         40         50         60         70         80         90        100        110
qnrB-1             --TAGGCATT GAAAATTCGC CACTGCCGCG CACAAGGCGC AGATTTCCGC GGTGCAAGCT TTATGAATAT GATCACCACG CGCACCTGGT TTTGCAGCGC ATATATCACT
qnrB-2             -ATGGGCATT GAAAAAT-GC CACTGCCGCG CACAAGGCGC AGATTTCCGC GGTGCAAGCT TTATGAATAT GATCACCACG CGCACCTGGT TTTGCAGCGC ATATATCACT
qnrB-3             CAGGGGCATT GAAATT-GC CACTGCCGCG CACAAGGCGC AGATTTCCGC GGTGCAAGCT TTATGAATAT GATCACCACG CGCACCTGGT TTTGCAGCGC ATATATCACT
qnrB-4             --TGGGCATT GAAA-TTCGC CACTGCCGCG CACAAGGCGC AGATTTCCGC GGTGCAAGCT TTATGAATAT GATCACCACG CGCACCTGGT TTTGCAGCGC ATATATCACT
qnrB-5             -AGAGGCATT GAAAATC-GC CACTGCCGCG CACAAGGCGC AGATTTCCGC GGTGCAAGCT TTATGAATAT GATCACCACG CGCACCTGGT TTTGCAGCGC ATATATCACT
qnrB-6             ---GGGCATT GAAAAATCGC CACTGCCGCG CACAAGGCGC AGATTTCCGC GGTGCAAGCT TTATGAATAT GATCACCACG CGCACCTGGT TTTGCAGCGC ATATATCACT
qnrB-7             ---TGGCATT GAAAATC-GC CACTGCCGCG CACAAGGCGC AGATTTCCGC GGTGCAAGCT TTATGAATAT GATCACCACG CGCACCTGGT TTTGCAGCGC ATATATCACT
qnrB-8             ---AGGCATT GAAATATCGC CACTGCCGCG CACAAGGCGC AGATTTCCGC GGTGCAAGCT TTATGAATAT GATCACCACG CGCACCTGGT TTTGCAGCGC ATATATCACT
qnrB-9             --TGGGCATT GAAAATC-GC CACTGCCGCG CACAAGGCGC AGATTTCCGC GGTGCAAGCT TTATGAATAT GATCACCACG CGCACCTGGT TTTGCAGCGC ATATATCACT
qnrB-10            -TCAGGCATT GAAAGAATTC CACTGCCGCG CACAAGGCGC AGATTTCCGC GGTGCAAGCT TTATGAATAT GATCACCACG CGCACCTGGT TTTGCAGCGC ATATATCACT
qnrB-11            -AGAGGCATT GAAAATC-GC CACTGCCGCG CACAAGGCGC AGATTTCCGC GGTGCAAGCT TTATGAATAT GATCACCACG CGCACCTGGT TTTGCAGCGC ATATATCACT
qnrB-12            ---GGGCATT GAAAAATCGC CACTGCCGCG CACAAGGCGC AGATTTCCGC GGTGCAAGCT TTATGAATAT GATCACCACG CGCACCTGGT TTTGCAGCGC ATATATCACT
Clustal Consensus      ****** ****     * ********** ********** ********** ********** ********** ********** ********** ********** **********

                   ....|....| ....|....| ....|....| ....|....| ....|....| ....|....| ....|....| ....|....| ....|....| ....|....| ....|....|
                         120        130        140        150        160        170        180        190        200        210        220
qnrB-1             AATACTAATC TAAGCTACGC CAATTTTTCG AAAGTCGTGT TGGAAAAGTG TGAGCTGTGG GAAAACCGCT GGATGGGGAC TCAGGTACTG GGTGCGACGT TCAGTGGTTC
qnrB-2             AATACTAATC TAAGCTACGC CAATTTTTCG AAAGTCGTGT TGGAAAAGTG TGAGCTGTGG GAAAACCGCT GGATGGGGAC TCAGGTACTG GGTGCGACGT TCAGTGGTTC
qnrB-3             AATACTAATC TAAGCTACGC CAATTTTTCG AAAGTCGTGT TGGAAAAGTG TGAGCTGTGG GAAAACCGCT GGATGGGGAC TCAGGTACTG GGTGCGACGT TCAGTGGTTC
qnrB-4             AATACTAATC TAAGCTACGC CAATTTTTCG AAAGTCGTGT TGGAAAAGTG TGAGCTGTGG GAAAACCGCT GGATGGGGAC TCAGGTACTG GGTGCGACGT TCAGTGGTTC
qnrB-5             AATACTAATC TAAGCTACGC CAATTTTTCG AAAGTCGTGT TGGAAAAGTG TGAGCTGTGG GAAAACCGCT GGATGGGGAC TCAGGTACTG GGTGCGACGT TCAGTGGTTC
qnrB-6             AATACTAATC TAAGCTACGC CAATTTTTCG AAAGTCGTGT TGGAAAAGTG TGAGCTGTGG GAAAACCGCT GGATGGGGAC TCAGGTACTG GGTGCGACGT TCAGTGGTTC
qnrB-7             AATACTAATC TAAGCTACGC CAATTTTTCG AAAGTCGTGT TGGAAAAGTG TGAGCTGTGG GAAAACCGCT GGATGGGGAC TCAGGTACTG GGTGCGACGT TCAGTGGTTC
qnrB-8             AATACTAATC TAAGCTACGC CAATTTTTCG AAAGTCGTGT TGGAAAAGTG TGAGCTGTGG GAAAACCGCT GGATGGGGAC TCAGGTACTG GGTGCGACGT TCAGTGGTTC
qnrB-9             AATACTAATC TAAGCTACGC CAATTTTTCG AAAGTCGTGT TGGAAAAGTG TGAGCTGTGG GAAAACCGCT GGATGGGGAC TCAGGTACTG GGTGCGACGT TCAGTGGTTC
qnrB-10            AATACTAATC TAAGCTACGC CAATTTTTCG AAAGTCGTGT TGGAAAAGTG TGAGCTGTGG GAAAACCGCT GGATGGGGAC TCAGGTACTG GGTGCGACGT TCAGTGGTTC
qnrB-11            AATACTAATC TAAGCTACGC CAATTTTTCG AAAGTCGTGT TGGAAAAGTG TGAGCTGTGG GAAAACCGCT GGATGGGGAC TCAGGTACTG GGTGCGACGT TCAGTGGTTC
qnrB-12            AATACTAATC TAAGCTACGC CAATTTTTCG AAAGTCGTGT TGGAAAAGTG TGAGCTGTGG GAAAACCGCT GGATGGGGAC TCAGGTACTG GGTGCGACGT TCAGTGGTTC
Clustal Consensus  ********** ********** ********** ********** ********** ********** ********** ********** ********** ********** **********

                   ....|....| ....|....| ....|....| ....|....| ....|....| ..
                         230        240        250        260        270
qnrB-1             AGATCTCTCC GGCGGCGAGT TTTCGACTTT CGA-TGGCGG AACAGCAAAA --
qnrB-2             AGATCTCTCC GGCGGCGAGT TTTCGACTTT CGACTGGA-A AACAGCAAAA A-
qnrB-3             AGATCTCTCC GGCGGCGAGT TTTCGACTTT CGACTGGGGA AACAGCAAAA --
qnrB-4             AGATCTCTCC GGCGGCGAGT TTTCGACTTT CGACTGGCGG AACAGCAAAA --
qnrB-5             AGATCTCTCC GGCGGCGAGT TTTCGACTTT CGACTGGC-G AGCAGCAAAA --
qnrB-6             AGATCTCTCC GGCGGCGAGT TTTCGACTTT CGACTGCGGA AACAGCAAAA TA
qnrB-7             AGATCTCTCC GGCGGCGAGT TTTCGACTTT CGACTGGC-G AACGGCAAAA --
qnrB-8             AGATCTCTCC GGCGGCGAGT TTTCGACTTT CGA-TGCGGG AACAGCAAAA --
qnrB-9             AGATCTCTCC GGCGGCGAGT TTTCGACTTT CGACTGGG-- AACAGCAAAA A-
qnrB-10            AGATCTCTCC GGCGGCGAGT TTTCGACTTT CGA-TGCGGT AACAGCAAAA --
qnrB-11            AGATCTCTCC GGCGGCGAGT TTTCGACTTT CGACTGGC-G AGCAGCAAAA --
qnrB-12            AGATCTCTCC GGCGGCGAGT TTTCGACTTT CGACTGCGGA AACAGCAAAA TA
Clustal Consensus  ********** ********** ********** *** **     * * ******
```

Note: "*" means no difference in nucleotide. Due to the instability of Sanger sequencing, it is common that there are differences in the first and last 20 nucleotides.

Figure S15. Homogeneity of *gyrA* in plasmid DNA reference materials.

```
Alignment: gyrA1

                     ....|....| ....|....| ....|....| ....|....| ....|....| ....|....| ....|....| ....|....| ....|
                             10         20         30         40         50         60         70         80
gyrA1-1              SLVTPFGKYH PHGDSAVYNT IVRMAQPFSL RYMLVDGQGN FGSIDGDSAA AMRYTEIRLA KIAHELMADL EKETVDFVDT LTVR-
gyrA1-2              -RWSRIGKYH PHGDSAVYNT IVRMAQPFSL RYMLVDGQGN FGSIDGDSAA AMRYTEIRLA KIAHELMADL EKETVDFVDN WNGT-
gyrA1-3              -RWSRIGKYH PHGDSAVYNT IVRMAQPFSL RYMLVDGQGN FGSIDGDSAA AMRYTEIRLA KIAHELMADL EKETVDFVDN WNGT-
gyrA1-4              -VGDVIGKYH PHGDSAVYNT IVRMAQPFSL RYMLVDGQGN FGSIDGDSAA AMRYTEIRLA KIAHELMADL EKETVDFVDN YDGTE
gyrA1-5              SLATRIGKYH PHGDSAVYNT IVRMAQPFSL RYMLVDGQGN FGSIDGDSAA AMRYTEIRLA KIAHELMADL EKETVDFVDK TERY-
gyrA1-6              LLVNLFGKYH PHGDSAVYNT IVRMAQPFSL RYMLVDGQGN FGSIDGDSAA AMRYTEIRLA KIAHELMADL EKETVDFVDK RERYG
gyrA1-7              LLVNLFGKYH PHGDSAVYNT IVRMAQPFSL RYMLVDGQGN FGSIDGDSAA AMRYTEIRLA KIAHELMADL EKETVDFVDK RERYG
gyrA1-8              -VGERFGKYH PHGDSAVYNT IVRMAQPFSL RYMLVDGQGN FGSIDGDSAA AMRYTEIRLA KIAHELMADL EKETVDFVDK CERYG
gyrA1-9              SLVSVIGKYH PHGDSAVYNT IVRMAQPFSL RYMLVDGQGN FGSIDGDSAA AMRYTEIRLA KIAHELMADL EKETVDFVDN YDGT-
gyrA1-10             -RWSRIGKYH PHGDSAVYNT IVRMAQPFSL RYMLVDGQGN FGSIDGDSAA AMRYTEIRLA KIAHELMADL EKETVDFVDN WNGT-
gyrA1-11             LLVNLFGKYH PHGDSAVYNT IVRMAQPFSL RYMLVDGQGN FGSIDGDSAA AMRYTEIRLA KIAHELMADL EKETVDFVDK RERYG
gyrA1-12             -VGDPIGKYH PHGDSAVYNT IVRMAQPFSL RYMLVDGQGN FGSIDGDSAA AMRYTEIRLA KIAHELMADL EKETVDFVDN WQRY-
Clustal Consensus         :**** ********** ********** ********** ********** ********** ********** *********.
```

```
Alignment: gyrA2

                     ....|....| ....|....| ....|....| ....|....| ....|....| ....|....| ....|....| ....|....| ....|
                             10         20         30         40         50         60         70         80
gyrA2-1              -VGDGFGKYH PHGDYAVYDT IVRMAQPFSL RYMLVDGQGN FGSIDGDSAA AMRYTEIRLA KIAHELMADL EKETVDFVDK WERYG
gyrA2-2              -VADRFGKYH PHGDYAVYDT IVRMAQPFSL RYMLVDGQGN FGSIDGDSAA AMRYTEIRLA KIAHELMADL EKETVDFVDN NDGT-
gyrA2-3              SLVNGIGKYH PHGDYAVYDT IVRMAQPFSL RYMLVDGQGN FGSIDGDSAA AMRYTEIRLA KIAHELMADL EKETVDFVDN WDGT-
gyrA2-4              -VGDGIGKYH PHGDYAVYDT IVRMAQPFSL RYMLVDGQGN FGSIDGDSAA AMRYTEIRLA KIAHELMADL EKETVDFVDN WERY-
gyrA2-5              -VGDVIGKYH PHGDYAVYDT IVRMAQPFSL RYMLVDGQGN FGSIDGDSAA AMRYTEIRLA KIAHELMADL EKETVDFVDN YDGTG
gyrA2-6              -VGHGIGKYH PHGDYAVYDT IVRMAQPFSL RYMLVDGQGN FGSIDGDSAA AMRYTEIRLA KIAHELMADL EKETVDFVDN LARY-
gyrA2-7              -VADRFGKYH PHGDYAVYDT IVRMAQPFSL RYMLVDGQGN FGSIDGDSAA AMRYTEIRLA KIAHELMADL EKETVDFVDN NDGT-
gyrA2-8              RWSSAFGKYH PHGDYAVYDT IVRMAQPFSL RYMLVDGQGN FGSIDGDSAA AMRYTEIRLA KIAHELMADL EKETVDFVDK SNGT-
gyrA2-9              SLVTVIGKYH PHGDYAVYDT IVRMAQPFSL RYMLVDGQGN FGSIDGDSAA AMRYTEIRLA KIAHELMADL EKETVDFVDN YDGT-
gyrA2-10             SLVNGIGKYH PHGDYAVYDT IVRMAQPFSL RYMLVDGQGN FGSIDGDSAA AMRYTEIRLA KIAHELMADL EKETVDFVDN WDGT-
gyrA2-11             -RW-SVGKYH PHGDYAVYDT IVRMAQPFSL RYMLVDGQGN FGSIDGDSAA AMRYTEIRLA KIAHELMADL EKETVDFVDK LQTVR
gyrA2-12             -VGHGIGKYH PHGDYAVYDT IVRMAQPFSL RYMLVDGQGN FGSIDGDSAA AMRYTEIRLA KIAHELMADL EKETVDFVDN LARY-
Clustal Consensus         .**** ********** ********** ********** ********** ********** ********** *********:
```

```
Alignment: gyrA3

                     ....|....| ....|....| ....|....| ....|....| ....|....| ....|....| ....|....| ....|....| ....|
                             10         20         30         40         50         60         70         80
gyrA3-1              SVGARVGKYH PHGDFAVYDT IVRMAQPFSL RYMLVDGQGN FGSIDGDSAA AMRYTEIRLA KIAHELMADL EKETVDFVDK M-TVR
gyrA3-2              HW-PRFGKYH PHGDFAVYDT IVRMAQPFSL RYMLVDGQGN FGSIDGDSAA AMRYTEIRLA KIAHELMADL EKETVDFVDT WERY-
gyrA3-3              -VGEPIGKYH PHGDFAVYDT IVRMAQPFSL RYMLVDGQGN FGSIDGDSAA AMRYTEIRLA KIAHELMADL EKETVDFVDN -QTVR
gyrA3-4              SLVVGFGKYH PHGDFAVYDT IVRMAQPFSL RYMLVDGQGN FGSIDGDSAA AMRYTEIRLA KIAHELMADL EKETVDFVDK SNGT-
gyrA3-5              -LVTRFGKYH PHGDFAVYDT IVRMAQPFSL RYMLVDGQGN FGSIDGDSAA AMRYTEIRLA KIAHELMADL EKETVDFVDK MGRYG
gyrA3-6              SLVSVIGKYH PHGDFAVYDT IVRMAQPFSL RYMLVDGQGN FGSIDGDSAA AMRYTEIRLA KIAHELMADL EKETVDFVDN YDGT-
gyrA3-7              -LVTRFGKYH PHGDFAVYDT IVRMAQPFSL RYMLVDGQGN FGSIDGDSAA AMRYTEIRLA KIAHELMADL EKETVDFVDK MGRYG
gyrA3-8              -VGEAFGKYH PHGDFAVYDT IVRMAQPFSL RYMLVDGQGN FGSIDGDSAA AMRYTEIRLA KIAHELMADL EKETVDFVDN CASVR
gyrA3-9              FVGDVIGKYH PHGDFAVYDT IVRMAQPFSL RYMLVDGQGN FGSIDGDSAA AMRYTEIRLA KIAHELMADL EKETVDFVDN YDGTE
gyrA3-10             -VGEPIGKYH PHGDFAVYDT IVRMAQPFSL RYMLVDGQGN FGSIDGDSAA AMRYTEIRLA KIAHELMADL EKETVDFVDN -QTVR
gyrA3-11             SLVIVIGKYH PHGDFAVYDT IVRMAQPFSL RYMLVDGQGN FGSIDGDSAA AMRYTEIRLA KIAHELMADL EKETVDFVDN YERYG
gyrA3-12             -VGDGFGKYH PHGDFAVYDT IVRMAQPFSL RYMLVDGQGN FGSIDGDSAA AMRYTEIRLA KIAHELMADL EKETVDFVDN MDGT-
Clustal Consensus         .**** ********** ********** ********** ********** ********** ********** *********.
```

```
Alignment: gyrA4

                     ....|....| ....|....| ....|....| ....|....| ....|....| ....|....| ....|....| ....|....| ....|
                             10         20         30         40         50         60         70         80
gyrA4-1              -VGERIGKYH PHGDFAVYGT IVRMAQPFSL RYMLVDGQGN FGSIDGDSAA AMRYTEIRLA KIAHELMADL EKETVDFVDN LARYG
gyrA4-2              SLVTRIGKYH PHGDFAVYGT IVRMAQPFSL RYMLVDGQGN FGSIDGDSAA AMRYTEIRLA KIAHELMADL EKETVDFVDN FKRY-
gyrA4-3              -W-PVIGKYH PHGDFAVYGT IVRMAQPFSL RYMLVDGQGN FGSIDGDSAA AMRYTEIRLA KIAHELMADL EKETVDFVDK WERYG
gyrA4-4              -VGHGIGKYH PHGDFAVYGT IVRMAQPFSL RYMLVDGQGN FGSIDGDSAA AMRYTEIRLA KIAHELMADL EKETVDFVDN WNGT-
gyrA4-5              LVGDRFGKYH PHGDFAVYGT IVRMAQPFSL RYMLVDGQGN FGSIDGDSAA AMRYTEIRLA KIAHELMADL EKETVDFVDK CARYG
gyrA4-6              -CW-PIGKYH PHGDFAVYGT IVRMAQPFSL RYMLVDGQGN FGSIDGDSAA AMRYTEIRLA KIAHELMADL EKETVDFVDN SNGTD
gyrA4-7              -CW-PIGKYH PHGDFAVYGT IVRMAQPFSL RYMLVDGQGN FGSIDGDSAA AMRYTEIRLA KIAHELMADL EKETVDFVDN SNGTD
gyrA4-8              -CW-PIGKYH PHGDFAVYGT IVRMAQPFSL RYMLVDGQGN FGSIDGDSAA AMRYTEIRLA KIAHELMADL EKETVDFVDN SNGTD
gyrA4-9              -VGERFGKYH PHGDFAVYGT IVRMAQPFSL RYMLVDGQGN FGSIDGDSAA AMRYTEIRLA KIAHELMADL EKETVDFVDK LNGT-
gyrA4-10             SLVRGFGKYH PHGDFAVYGT IVRMAQPFSL RYMLVDGQGN FGSIDGDSAA AMRYTEIRLA KIAHELMADL EKETVDFVDK MNGT-
gyrA4-11             IVGDVIGKYH PHGDFAVYGT IVRMAQPFSL RYMLVDGQGN FGSIDGDSAA AMRYTEIRLA KIAHELMADL EKETVDFVDN YVRYG
gyrA4-12             -VGHGIGKYH PHGDFAVYGT IVRMAQPFSL RYMLVDGQGN FGSIDGDSAA AMRYTEIRLA KIAHELMADL EKETVDFVDN WNGT-
Clustal Consensus         :**** ********** ********** ********** ********** ********** ********** *********:
```

```
Alignment: gyrA5

                     ....|....| ....|....| ....|....| ....|....| ....|....| ....|....| ....|....| ....|....| ....|
                             10         20         30         40         50         60         70         80
gyrA5-1              -VGDPFGKYH PHGDFAVYAT IVRMAQPFSL RYMLVDGQGN FGSIDGDSAA AMRYTEIRLA KIAHELMADL EKETVDFVDN LGRYG
gyrA5-2              -LVHRIGKYH PHGDFAVYAT IVRMAQPFSL RYMLVDGQGN FGSIDGDSAA AMRYTEIRLA KIAHELMADL EKETVDFVDN WNGT-
gyrA5-3              SLVVGFGKYH PHGDFAVYAT IVRMAQPFSL RYMLVDGQGN FGSIDGDSAA AMRYTEIRLA KIAHELMADL EKETVDFVDN C-RY-
gyrA5-4              SLVNGFGKYH PHGDFAVYAT IVRMAQPFSL RYMLVDGQGN FGSIDGDSAA AMRYTEIRLA KIAHELMADL EKETVDFVDK LDGT-
gyrA5-5              -VGEGFGKYH PHGDFAVYAT IVRMAQPFSL RYMLVDGQGN FGSIDGDSAA AMRYTEIRLA KIAHELMADL EKETVDFVDN MSRYG
gyrA5-6              SLVNGFGKYH PHGDFAVYAT IVRMAQPFSL RYMLVDGQGN FGSIDGDSAA AMRYTEIRLA KIAHELMADL EKETVDFVDK LDGT-
gyrA5-7              -VGEGFGKYH PHGDFAVYAT IVRMAQPFSL RYMLVDGQGN FGSIDGDSAA AMRYTEIRLA KIAHELMADL EKETVDFVDN MSRYG
gyrA5-8              -LVHRIGKYH PHGDFAVYAT IVRMAQPFSL RYMLVDGQGN FGSIDGDSAA AMRYTEIRLA KIAHELMADL EKETVDFVDN WNGT-
gyrA5-9              -VGDRIGKYH PHGDFAVYAT IVRMAQPFSL RYMLVDGQGN FGSIDGDSAA AMRYTEIRLA KIAHELMADL EKETVDFVDN WPTVR
gyrA5-10             -VGDVIGKYH PHGDFAVYAT IVRMAQPFSL RYMLVDGQGN FGSIDGDSAA AMRYTEIRLA KIAHELMADL EKETVDFVDN K-RY-
gyrA5-11             CW-PVIGKYH PHGDFAVYAT IVRMAQPFSL RYMLVDGQGN FGSIDGDSAA AMRYTEIRLA KIAHELMADL EKETVDFVDK RNGT-
gyrA5-12             RW-AGIGKYH PHGDFAVYAT IVRMAQPFSL RYMLVDGQGN FGSIDGDSAA AMRYTEIRLA KIAHELMADL EKETVDFVDN LDGT-
Clustal Consensus         :**** ********** ********** ********** ********** ********** ********** *********:
```

Note: “*” means no difference in amino acid. Due to the instability of Sanger sequencing, it is common that there are differences in the first and last 10 amino acids.

Figure S16. PCR results for storage stability of plasmid DNA references materials stored at 37°C.

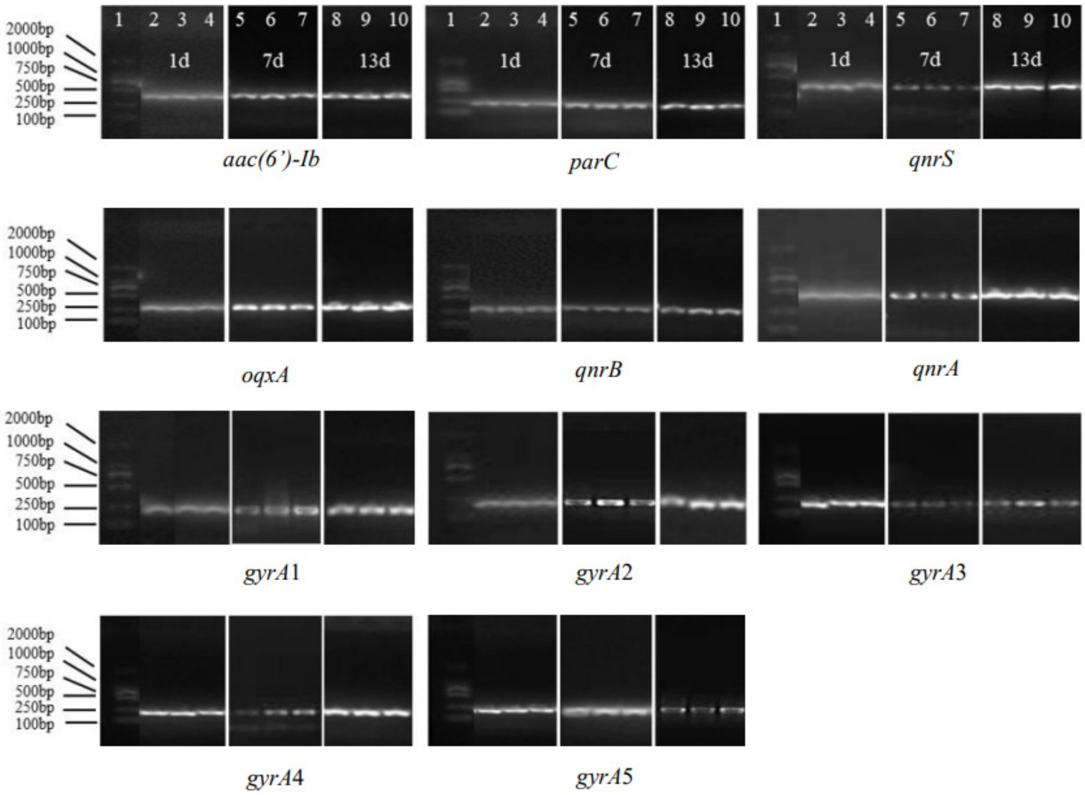

Note: Lane 1 is DL 2000 DNA marker; lane 2–4, 5–7, and 8–10 are PCR bands of the target gene sampled on the 1st, 7th, and 13th days of storage, respectively. Considering the different sampling timepoints, the gene images were intercepted and spliced using Microsoft Word, and the original images are available upon request.

Figure S17. Sequencing results of *aac(6')-Ib* for stability of plasmid DNA references materials stored at 37°C.

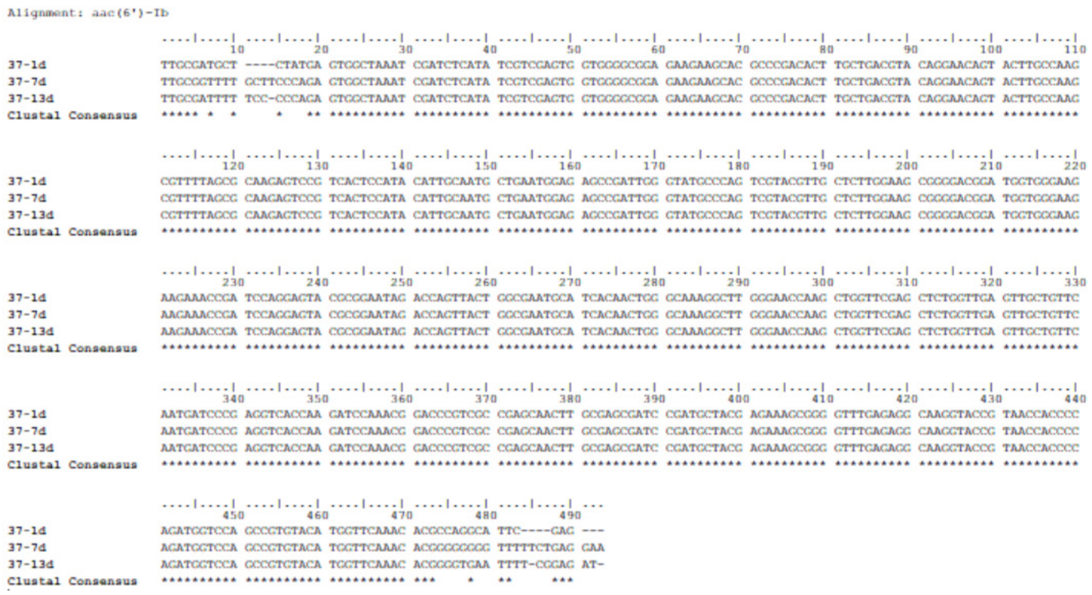

Note: "*" means no difference in nucleotide. Due to the instability of Sanger sequencing, it is common that there are differences in the first and last 20 nucleotides

Figure S18. Sequencing results of *parC* for stability of plasmid DNA references materials stored at 37°C.

```
Alignment: parC

                    ....|....| ....|....| ....|....| ....|....| ....|....| ....|....| ....|....| ....|....| ....|....
                            10         20         30         40         50         60         70         80
37-1d               -YAMSELGLN ATAKFKKSAR TVGDVLGKYH PHGDRACYEA MVLMAQPFSY RYPLVDGQGN WGAPDDPKSF AAMRYTESRL SKYAELLL-
37-7d               SMRFVKLGLN ATAKFKKSAR TVGDVLGKYH PHGDRACYEA MVLMAQPFSY RYPLVDGQGN WGAPDDPKSF AAMRYTESRL SKYAGLLL-
37-13d              SMRFVELGLN ATAKFKKSAR TVGDVLGKYH PHGDRACYEA MVLMAQPFSY RYPLVDGQGN WGAPDDPKSF AAMRYTESRL SKYAGPAV-
Clustal Consensus     : :**** ********** ********** ********** ********** ********** ********** ********** ****    :
```

Note: "*" means no difference in amino acid. Due to the instability of Sanger sequencing, it is common that there are differences in the first and last 10 amino acids.

Figure S19. Sequencing results of *qnrS* for stability of plasmid DNA references materials stored at 37°C.

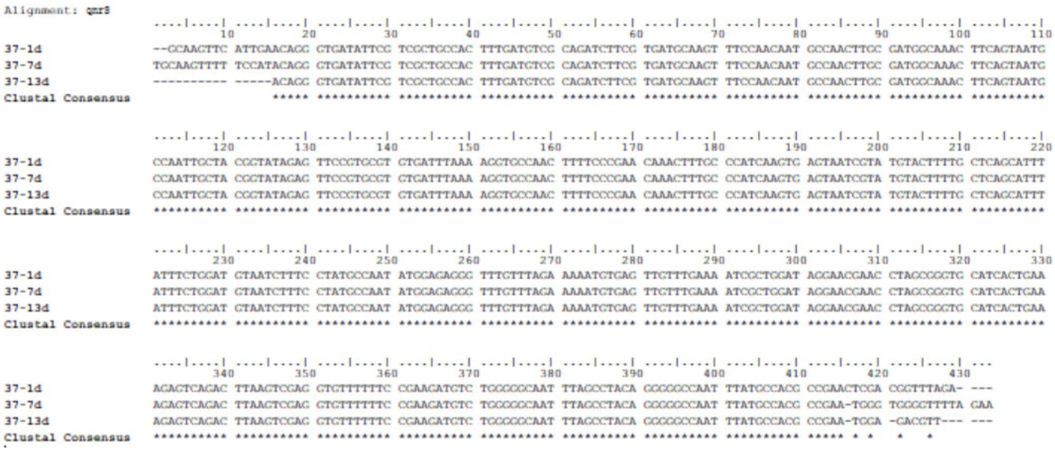

Note: "*" means no difference in nucleotide. Due to the instability of Sanger sequencing, it is common that there are differences in the first and last 20 nucleotides.

Figure S20. Sequencing results of *oqxA* for stability of plasmid DNA references materials stored at 37°C.

```
Alignment: OQXA

                    ....|....| ....|....| ....|....| ....|....| ....|....| ....|....| ....|....| ....|....| ....|....| ....|....| ....|....|
                           10         20         30         40         50         60         70         80         90        100        110
37-1d               GACAGCGTCG CACAGAATGC TGCGCCTCCC GCCCCGACGG TCAGCGCCGC TAAGGTGCTG GTGAAGTCGA TCAGTCAGTG GGATAGTTTT AACGGTCGCA TTGAAGCGGT
37-7d               ------GCGT CACAGAATGC TGCGCCTCCC GCCCCGACGG TCAGCGCCGC TAAGGTGCTG GTGAAGTCGA TCAGTCAGTG GGATAGTTTT AACGGTCGCA TTGAAGCGGT
37-13d              -----TCGCA CCCAGAATGC TGCGCCTCCC GCCCCGACGG TCAGCGCCGC TAAGGTGCTG GTGAAGTCGA TCAGTCAGTG GGATAGTTTT AACGGTCGCA TTGAAGCGGT
Clustal Consensus              * ******** ********** ********** ********** ********** ********** ********** ********** ********** **********

                    ....|....| ....|....| ....|....| ....|....| ....|....| ....|....| ....|....| ....|....| ....|....| ....|....| ....|....|
                          120        130        140        150        160        170        180        190        200        210        220
37-1d               GGAGAGCGTT CAGCTTCGCC CTCGCGTCTC GGGATACATT GATAAAGTGA ATTACACTGA CGGCCAGGAG GTGAAAAAGG GCCAGGTGCT GTTCACGATA GATGACAGAA
37-7d               GGAGAGCGTT CAGCTTCGCC CTCGCGTCTC GGGATACATT GATAAAGTGA ATTACACTGA CGGCCAGGAG GTGAAAAAGG GCCAGGTGCT GTTCACGATA GATGACAGAA
37-13d              GGAGAGCGTT CAGCTTCGCC CTCGCGTCTC GGGATACATT GATAAAGTGA ATTACACTGA CGGCCAGGAG GTGAAAAAGG GCCAGGTGCT GTTCACGATA GATGACAGAA
Clustal Consensus   ********** ********** ********** ********** ********** ********** ********** ********** ********** ********** **********

                    ....|....| ....|....| ....|....| ....|....| ....|....| ....|....| ....|....| ....|....| ....|....| ....|....| ....|....|
                          230        240        250        260        270        280        290        300        310        320        330
37-1d               CCTATCGCGC CGCGCTGGAG CAGGCGCAGG CGGCGTTGGC AAGAGCCAAA ACGCAGGCCA GCCTCGCGCA AAGCGAGGCG AACCGCACCG ATAAATTAGT CCATACCAAC
37-7d               CCTATCGCGC CGCGCTGGAG CAGGCGCAGG CGGCGTTGGC AAGAGCCAAA ACGCAGGCCA GCCTCGCGCA AAGCGAGGCG AACCGCACCG ATAAATTAGT CCATACCCCC
37-13d              CCTATCGCGC CGCGCTGGAG CAGGCGCAGG CGGCGTTGGC AAGAGCCAAA ACGCAGGCCA GCCTCGCGCA AAGCGAGGCG AACCGCACCG ATAAATTAGT CCATACCGCT
Clustal Consensus   ********** ********** ********** ********** ********** ********** ********** ********** ********** ********** *******

                    ....|....| ..
                          340
37-1d               CTCGTCTCC- --
37-7d               CCTCTCGTCT CC
37-13d              TTC------- --
Clustal Consensus
```

Note: "*" means no difference in nucleotide. Due to the instability of Sanger sequencing, it is common that there are differences in the first and last 20 nucleotides.

Figure S21. Sequencing results of *qnrB* for stability of plasmid DNA references materials stored at 37°C.

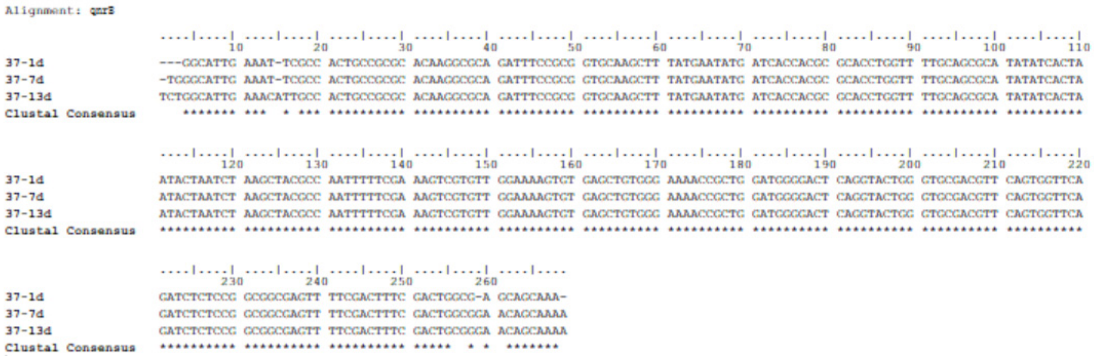

Note: “*” means no difference in nucleotide. Due to the instability of Sanger sequencing, it is common that there are differences in the first and last 20 nucleotides.

Figure S22. Sequencing results of *qnrA* for stability of plasmid DNA references materials stored at 37°C.

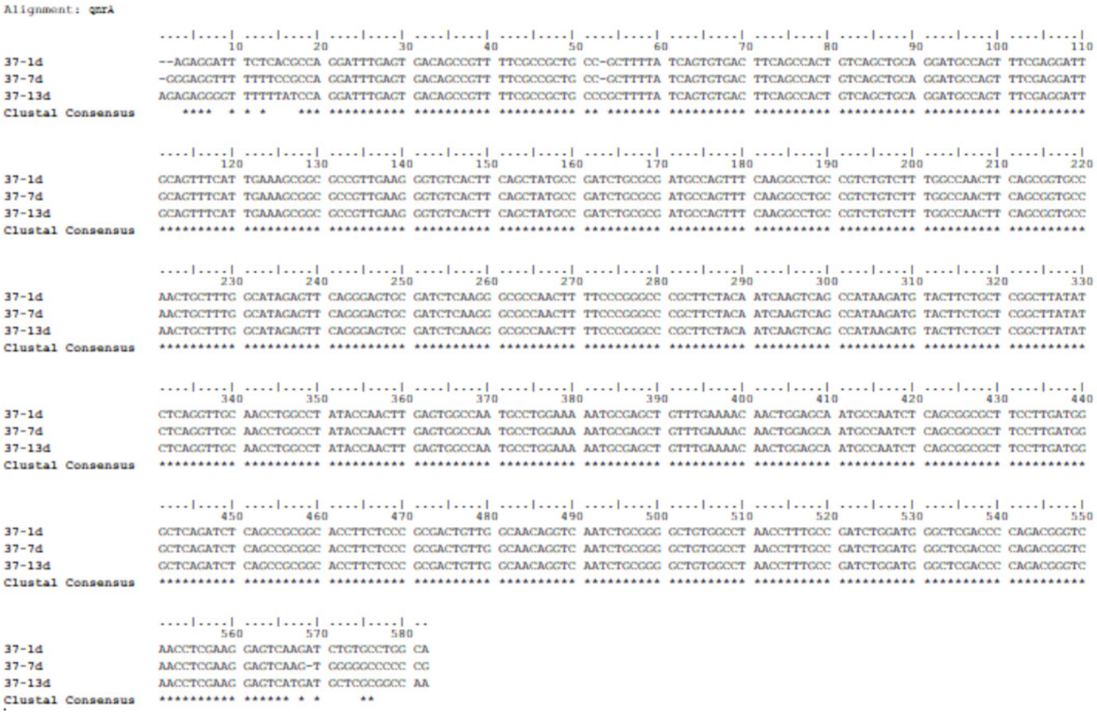

Note: “*” means no difference in nucleotide. Due to the instability of Sanger sequencing, it is common that there are differences in the first and last 20 nucleotides.

Figure S23. Sequencing results of *gyrA* for stability of plasmid DNA references materials stored at 37°C.

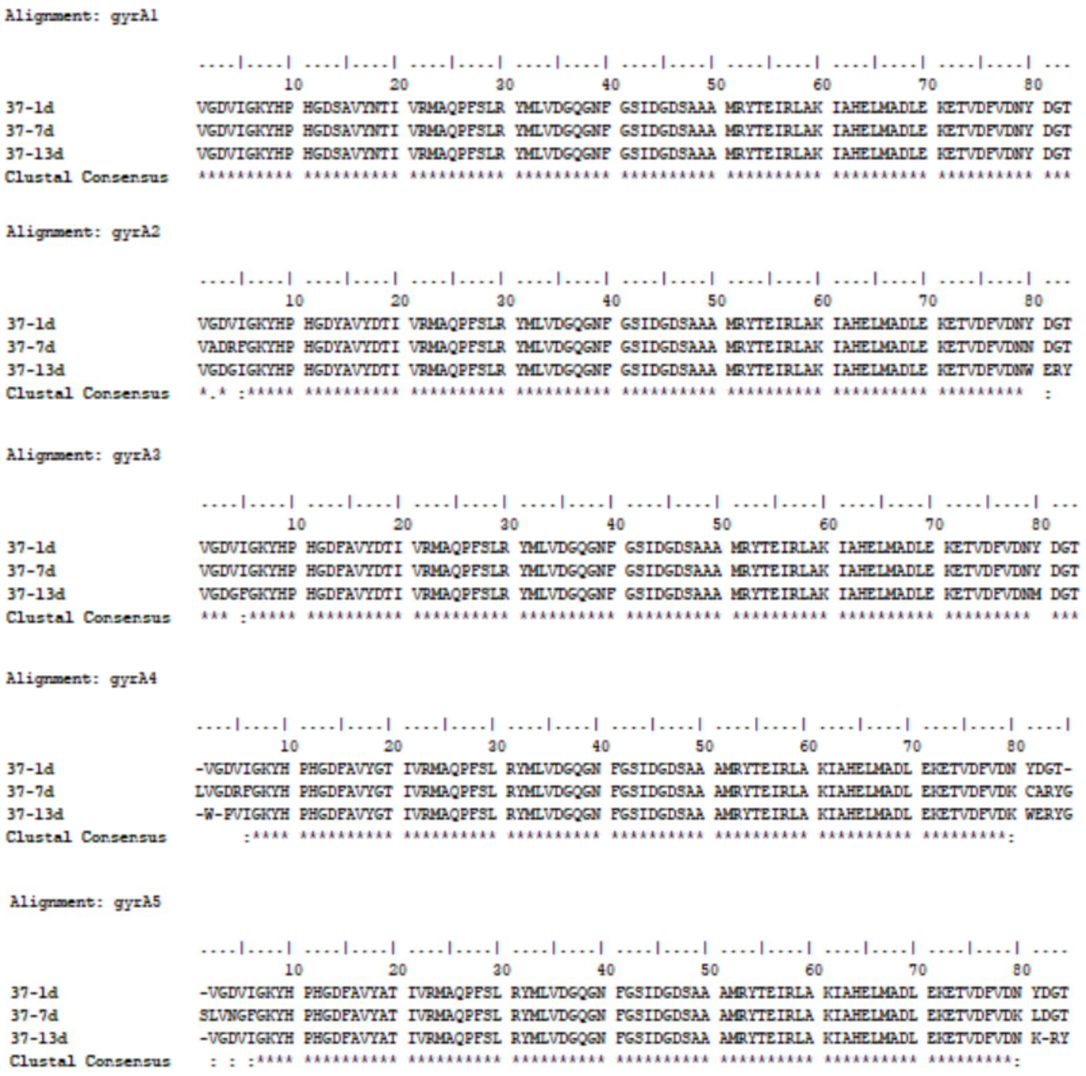

Note: "*" means no difference in amino acid. Due to the instability of Sanger sequencing, it is common that there are differences in the first and last 10 amino acids.

Figure S24. PCR results for storage stability of plasmid DNA references materials stored at 4°C.

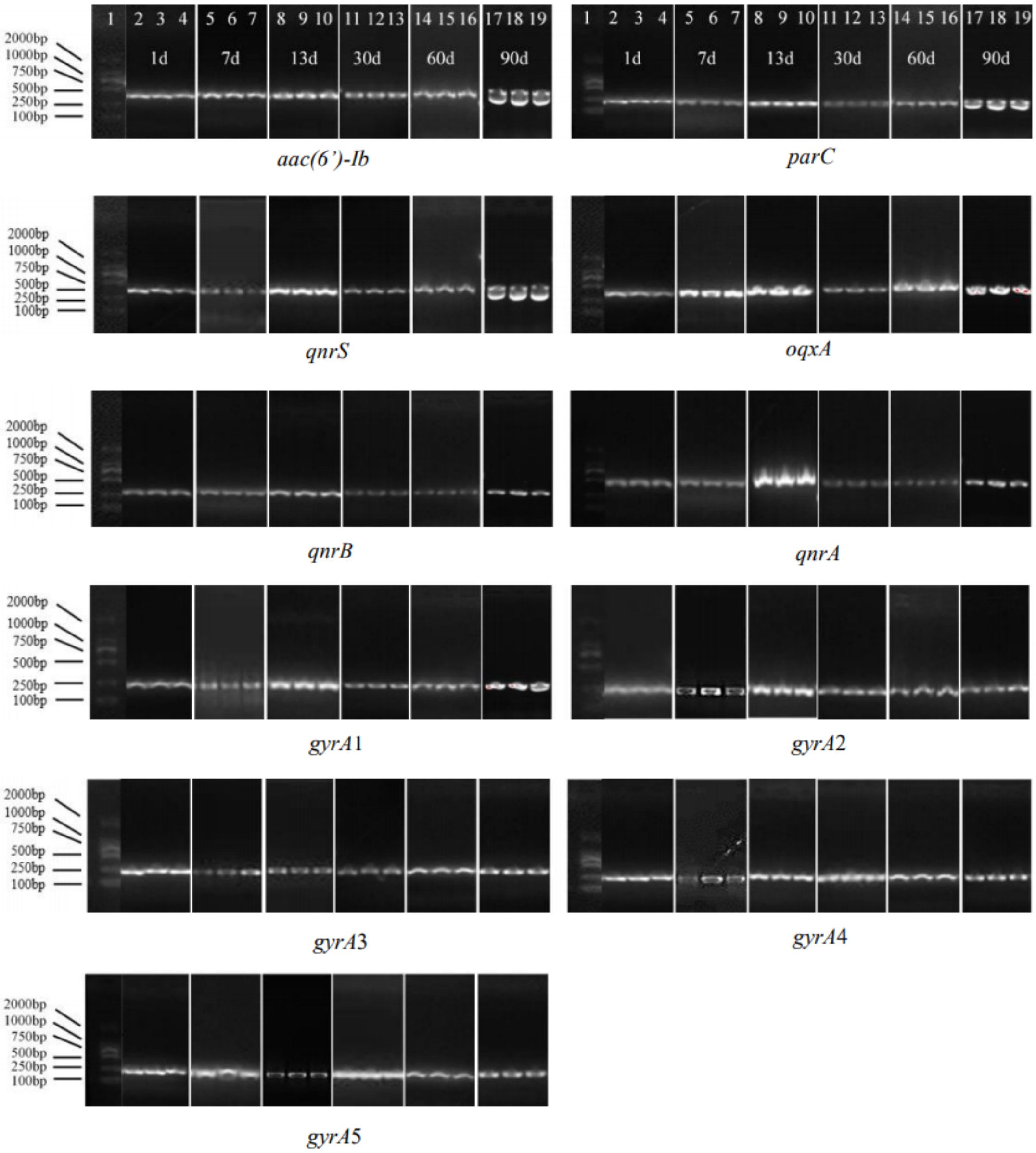

Note: Lane 1 is DL 2000 DNA marker; lane 2–4, 5–7, 8–10, 11–13, 14–16, and 17–19 are PCR bands of the target gene sampled on the 1st, 7th, 13th, 30th, 60th, and 90th days of storage, respectively. Considering the different sampling timepoints, the gene images were intercepted and spliced using Microsoft Word, and the original images are available upon request.

Figure S25. Sequencing results of *aac(6')-Ib* for stability of plasmid DNA references materials stored at 4°C.

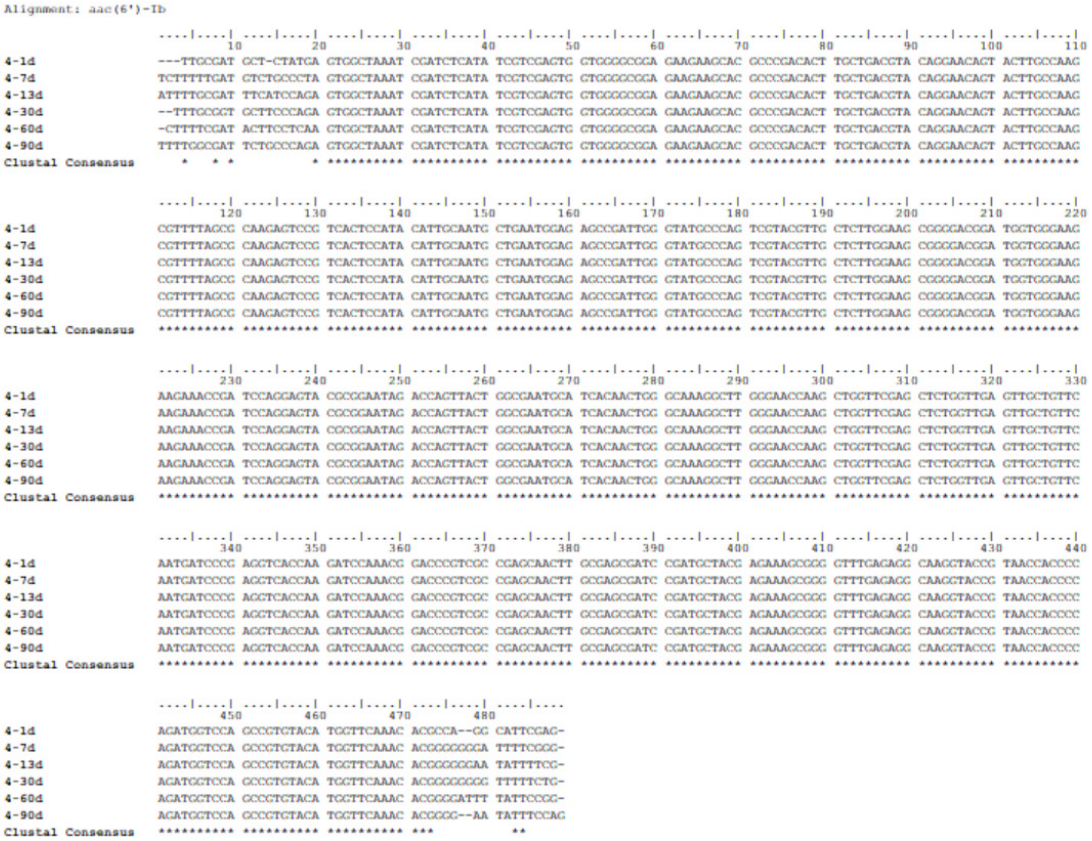

Note: "*" means no difference in nucleotide. Due to the instability of Sanger sequencing, it is common that there are differences in the first and last 20 nucleotides.

Figure S26. Sequencing results of *parC* for stability of plasmid DNA references materials stored at 4°C.

```
Alignment: parC

                   ....|....| ....|....| ....|....| ....|....| ....|....| ....|....| ....|....| ....|....| ....|....
                           10         20         30         40         50         60         70         80
4-1d               -YAMSELGLN ATAKFKKSAR TVGDVLGKYH PHGDRACYEA MVLMAQPFSY RYPLVDGQGN WGAPDDPKSF AAMRYTESRL SKYAELLL-
4-7d               -YAICELGLN ATAKFKKSAR TVGDVLGKYH PHGDRACYEA MVLMAQPFSY RYPLVDGQGN WGAPDDPKSF AAMRYTESRL SKYAGCCS-
4-13d              SMRFVELGLN ATAKFKKSAR TVGDVLGKYH PHGDRACYEA MVLMAQPFSY RYPLVDGQGN WGAPDDPKSF AAMRYTESRL SKYAVICCL
4-30d              SMRFVKLGLN ATAKFKKSAR TVGDVLGKYH PHGDRACYEA MVLMAQPFSY RYPLVDGQGN WGAPDDPKSF AAMRYTESRL SKYAGLLL-
4-60d              FYAMVELGLN ATAKFKKSAR TVGDVLGKYH PHGDRACYEA MVLMAQPFSY RYPLVDGQGN WGAPDDPKSF AAMRYTESRL SKYAGVCCL
4-90d              SMRFVKLGLN ATAKFKKSAR TVGDVLGKYH PHGDRACYEA MVLMAQPFSY RYPLVDGQGN WGAPDDPKSF AAMRYTESRL SKYAGLLL-
Clustal Consensus     : :**** ********** ********** ********** ********** ********** ********** ********** ****
```

Note: "*" means no difference in amino acid. Due to the instability of Sanger sequencing, it is common that there are differences in the first and last 10 amino acids.

Figure S27. Sequencing results of *qnrS* for stability of plasmid DNA references materials stored at 4°C.

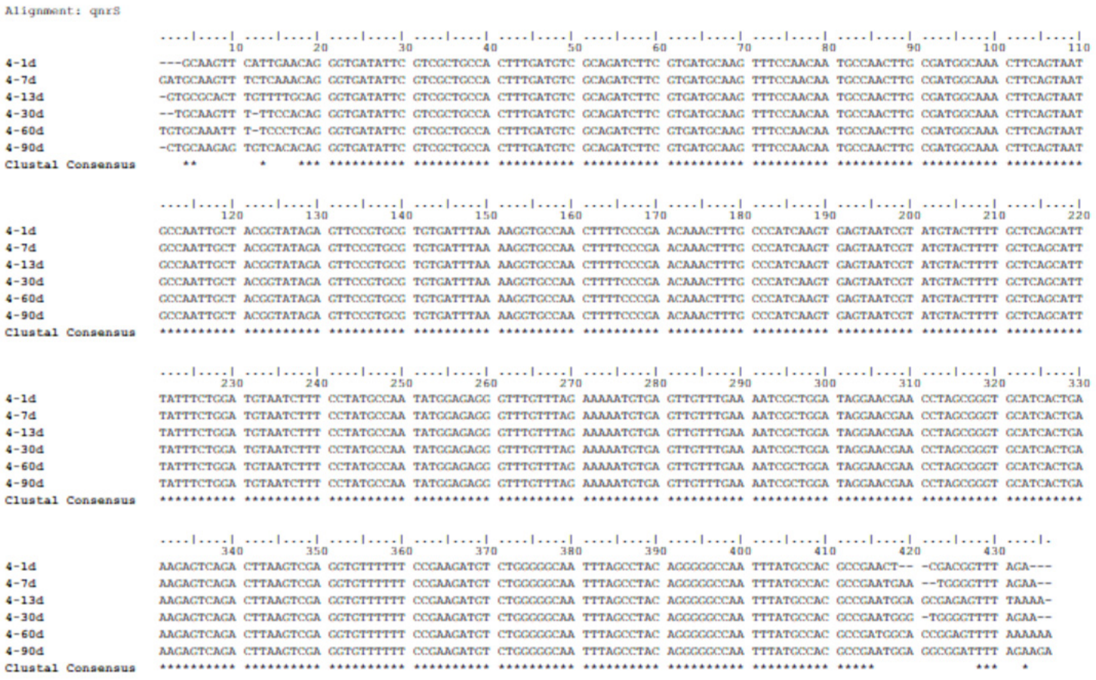

Note: "*" means no difference in nucleotide. Due to the instability of Sanger sequencing, it is common that there are differences in the first and last 20 nucleotides.

Figure S28. Sequencing results of *oqxA* for stability of plasmid DNA references materials stored at 4°C.

```
Alignment: oqxA

                   ....|....| ....|....| ....|....| ....|....| ....|....| ....|....| ....|....| ....|....| ....|....| ....|....| ....|....|
                          10         20         30         40         50         60         70         80         90        100        110
4-1d               ---GACAGCG -TCGCACAGA ATGCTGCGCC TCCCGCCCCG ACGGTCAGCG CCGCTAAGGT GCTGGTGAAG TCGATCAGTC AGTGGGATAG TTTTAACGGT CGCATTGAAG
4-7d               CCAGACAGCT GTCCCCAAGA ATGCTGCGCC TCCCGCCCCG ACGGTCAGCG CCGCTAAGGT GCTGGTGAAG TCGATCAGTC AGTGGGATAG TTTTAACGGT CGCATTGAAG
4-13d              -CAGACAGCG -TTGTCCAGA ATGCTGCGCC TCCCGCCCCG ACGGTCAGCG CCGCTAAGGT GCTGGTGAAG TCGATCAGTC AGTGGGATAG TTTTAACGGT CGCATTGAAG
4-30d              ---------T CGCACCCAGA ATGCTGCGCC TCCCGCCCCG ACGGTCAGCG CCGCTAAGGT GCTGGTGAAG TCGATCAGTC AGTGGGATAG TTTTAACGGT CGCATTGAAG
4-60d              -AGAACAGCG CTTGCCCCAA ATGCTGCGCC TCCCGCCCCG ACGGTCAGCG CCGCTAAGGT GCTGGTGAAG TCGATCAGTC AGTGGGATAG TTTTAACGGT CGCATTGAAG
4-90d              -TGGACAGCG TTTGCGCAGA ATGCTGCGCC TCCCGCCCCG ACGGTCAGCG CCGCTAAGGT GCTGGTGAAG TCGATCAGTC AGTGGGATAG TTTTAACGGT CGCATTGAAG
Clustal Consensus           * ********** ********** ********** ********** ********** ********** ********** ********** ********** **********

                   ....|....| ....|....| ....|....| ....|....| ....|....| ....|....| ....|....| ....|....| ....|....| ....|....| ....|....|
                          120        130        140        150        160        170        180        190        200        210        220
4-1d               CGGTGGAGAG CGTTCAGCTT CGCCCTCGCG TCTCGGGATA CATTGATAAA GTGAATTACA CTGACGGCCA GGAGGTGAAA AAGGGCCAGG TGCTGTTCAC GATAGATGAC
4-7d               CGGTGGAGAG CGTTCAGCTT CGCCCTCGCG TCTCGGGATA CATTGATAAA GTGAATTACA CTGACGGCCA GGAGGTGAAA AAGGGCCAGG TGCTGTTCAC GATAGATGAC
4-13d              CGGTGGAGAG CGTTCAGCTT CGCCCTCGCG TCTCGGGATA CATTGATAAA GTGAATTACA CTGACGGCCA GGAGGTGAAA AAGGGCCAGG TGCTGTTCAC GATAGATGAC
4-30d              CGGTGGAGAG CGTTCAGCTT CGCCCTCGCG TCTCGGGATA CATTGATAAA GTGAATTACA CTGACGGCCA GGAGGTGAAA AAGGGCCAGG TGCTGTTCAC GATAGATGAC
4-60d              CGGTGGAGAG CGTTCAGCTT CGCCCTCGCG TCTCGGGATA CATTGATAAA GTGAATTACA CTGACGGCCA GGAGGTGAAA AAGGGCCAGG TGCTGTTCAC GATAGATGAC
4-90d              CGGTGGAGAG CGTTCAGCTT CGCCCTCGCG TCTCGGGATA CATTGATAAA GTGAATTACA CTGACGGCCA GGAGGTGAAA AAGGGCCAGG TGCTGTTCAC GATAGATGAC
Clustal Consensus  ********** ********** ********** ********** ********** ********** ********** ********** ********** ********** **********

                   ....|....| ....|....| ....|....| ....|....| ....|....| ....|....| ....|....| ....|....| ....|....| ....|....| ....|....|
                          230        240        250        260        270        280        290        300        310        320        330
4-1d               AGAACCTATC GCGCCGCGCT GGAGCAGGCG CAGGCGGCGT TGGCAAGAGC CAAAACGCAG GCCAGCCTCG CGCAAAGCGA GGCGAACCGC ACCGATAAAT TAGTCCATAC
4-7d               AGAACCTATC GCGCCGCGCT GGAGCAGGCG CAGGCGGCGT TGGCAAGAGC CAAAACGCAG GCCAGCCTCG CGCAAAGCGA GGCGAACCGC ACCGATAAAT TAGTCCATAC
4-13d              AGAACCTATC GCGCCGCGCT GGAGCAGGCG CAGGCGGCGT TGGCAAGAGC CAAAACGCAG GCCAGCCTCG CGCAAAGCGA GGCGAACCGC ACCGATAAAT TAGTCCATAC
4-30d              AGAACCTATC GCGCCGCGCT GGAGCAGGCG CAGGCGGCGT TGGCAAGAGC CAAAACGCAG GCCAGCCTCG CGCAAAGCGA GGCGAACCGC ACCGATAAAT TAGTCCATAC
4-60d              AGAACCTATC GCGCCGCGCT GGAGCAGGCG CAGGCGGCGT TGGCAAGAGC CAAAACGCAG GCCAGCCTCG CGCAAAGCGA GGCGAACCGC ACCGATAAAT TAGTCCATAC
4-90d              AGAACCTATC GCGCCGCGCT GGAGCAGGCG CAGGCGGCGT TGGCAAGAGC CAAAACGCAG GCCAGCCTCG CGCAAAGCGA GGCGAACCGC ACCGATAAAT TAGTCCATAC
Clustal Consensus  ********** ********** ********** ********** ********** ********** ********** ********** ********** ********** **********

                   ....|....| ....|....
                          340
4-1d               CAACCTC--- GTCTCC---
4-7d               CCCCC-TCTC GTCTCCTGA
4-13d              C--------- ---------
4-30d              CGCTT-TC-- ---------
4-60d              CCCCCGTCCC GTCTCAAAA
4-90d              CCCCCCTCC- GTCTCAC--
Clustal Consensus  *
```

Note: "*" means no difference in nucleotide. Due to the instability of Sanger sequencing, it is common that there are differences in the first and last 20 nucleotides.

Figure S29. Sequencing results of *qnrB* for stability of plasmid DNA references materials stored at 4°C.

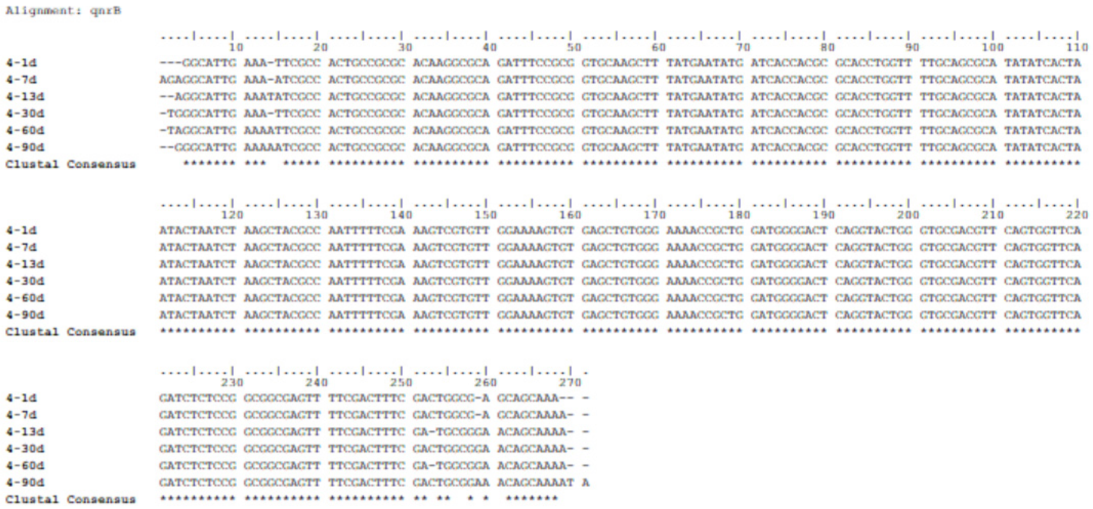

Note: “*” means no difference in nucleotide. Due to the instability of Sanger sequencing, it is common that there are differences in the first and last 20 nucleotides.

Figure S30. Sequencing results of *qnrA* for stability of plasmid DNA references materials stored at 4°C.

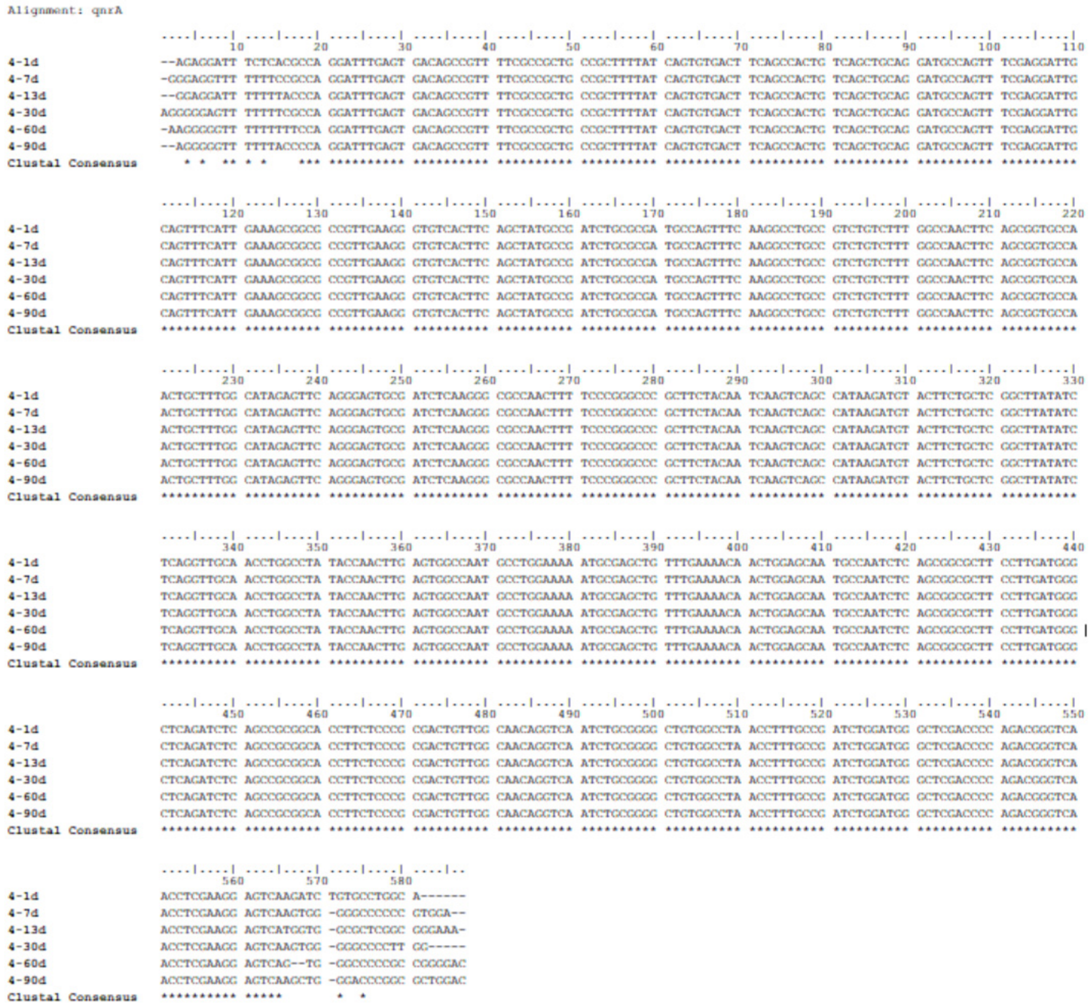

Note: "*" means no difference in nucleotide. Due to the instability of Sanger sequencing, it is common that there are differences in the first and last 20 nucleotides.

Figure S31. Sequencing results of *gyrA* for stability of plasmid DNA references materials stored at 4°C.

```
Alignment: gyrA1

                   ....|....| ....|....| ....|....| ....|....| ....|....| ....|....| ....|....| ....|....| ....|.
                           10         20         30         40         50         60         70         80
4-1d               -VG-DVIGKY HPHGDSAVYN TIVRMAQPFS LRYMLVDGQG NFGSIDGDSA AAMRYTEIRL AKIAHELMAD LEKETVDFVD NYDGT-
4-7d               LLV-NLFGKY HPHGDSAVYN TIVRMAQPFS LRYMLVDGQG NFGSIDGDSA AAMRYTEIRL AKIAHELMAD LEKETVDFVD KRERYG
4-13d              -VG-AGFGKY HPHGDSAVYN TIVRMAQPFS LRYMLVDGQG NFGSIDGDSA AAMRYTEIRL AKIAHELMAD LEKETVDFVD KSTVR-
4-30d              -VG-ERFGKY HPHGDSAVYN TIVRMAQPFS LRYMLVDGQG NFGSIDGDSA AAMRYTEIRL AKIAHELMAD LEKETVDFVD KCERYG
4-60d              -RW-PRFGKY HPHGDSAVYN TIVRMAQPFS LRYMLVDGQG NFGSIDGDSA AAMRYTEIRL AKIAHELMAD LEKETVDFVD TLTVR-
4-90d              -VG-DPIGKY HPHGDSAVYN TIVRMAQPFS LRYMLVDGQG NFGSIDGDSA AAMRYTEIRL AKIAHELMAD LEKETVDFVD NWQRY-
Clustal Consensus        :*** ********** ********** ********** ********** ********** ********** ********** .

Alignment: gyrA2

                   ....|....| ....|....| ....|....| ....|....| ....|....| ....|....| ....|....| ....|....| ....
                           10         20         30         40         50         60         70         80
4-1d               VGDVIGKYHP HGDYAVYDTI VRMAQPFSLR YMLVDGQGNF GSIDGDSAAA MRYTEIRLAK IAHELMADLE KETVDFVDNY DGT-
4-7d               VADRFGKYHP HGDYAVYDTI VRMAQPFSLR YMLVDGQGNF GSIDGDSAAA MRYTEIRLAK IAHELMADLE KETVDFVDNN DGT-
4-13d              VGDGFGKYHP HGDYAVYDTI VRMAQPFSLR YMLVDGQGNF GSIDGDSAAA MRYTEIRLAK IAHELMADLE KETVDFVDKW ERYG
4-30d              VADRFGKYHP HGDYAVYDTI VRMAQPFSLR YMLVDGQGNF GSIDGDSAAA MRYTEIRLAK IAHELMADLE KETVDFVDNN DGT-
4-60d              RW-SVGKYHP HGDYAVYDTI VRMAQPFSLR YMLVDGQGNF GSIDGDSAAA MRYTEIRLAK IAHELMADLE KETVDFVDKL QTVR
4-90d              VGHGIGKYHP HGDYAVYDTI VRMAQPFSLR YMLVDGQGNF GSIDGDSAAA MRYTEIRLAK IAHELMADLE KETVDFVDNL ARY-
Clustal Consensus      .***** ********** ********** ********** ********** ********** ********** ********:

Alignment: gyrA3

                   ....|....| ....|....| ....|....| ....|....| ....|....| ....|....| ....|....| ....|....| ....|
                           10         20         30         40         50         60         70         80
4-1d               -VGDVIGKYH PHGDFAVYDT IVRMAQPFSL RYMLVDGQGN FGSIDGDSAA AMRYTEIRLA KIAHELMADL EKETVDFVDN YDGT-
4-7d               -LVTRFGKYH PHGDFAVYDT IVRMAQPFSL RYMLVDGQGN FGSIDGDSAA AMRYTEIRLA KIAHELMADL EKETVDFVDK MGRYG
4-13d              -VGHGFGKYH PHGDFAVYDT IVRMAQPFSL RYMLVDGQGN FGSIDGDSAA AMRYTEIRLA KIAHELMADL EKETVDFVDK SETVR
4-30d              -VGDVIGKYH PHGDFAVYDT IVRMAQPFSL RYMLVDGQGN FGSIDGDSAA AMRYTEIRLA KIAHELMADL EKETVDFVDN YDGT-
4-60d              SVGARVGKYH PHGDFAVYDT IVRMAQPFSL RYMLVDGQGN FGSIDGDSAA AMRYTEIRLA KIAHELMADL EKETVDFVDK M-TVR
4-90d              -VGEAFGKYH PHGDFAVYDT IVRMAQPFSL RYMLVDGQGN FGSIDGDSAA AMRYTEIRLA KIAHELMADL EKETVDFVDN CASVR
Clustal Consensus   :   .**** ********** ********** ********** ********** ********** ********** *********:

Alignment: gyrA4

                   ....|....| ....|....| ....|....| ....|....| ....|....| ....|....| ....|....| ....|....| ....|..
                           10         20         30         40         50         60         70         80
4-1d               -VGDVIGKYH PHGDFAVYGT IVRMAQPFSL RYMLVDGQGN FGSIDGDSAA AMRYTEIRLA KIAHELMADL EKETVDFVDN --YDGT-
4-7d               -VGERFGKYH PHGDFAVYGT IVRMAQPFSL RYMLVDGQGN FGSIDGDSAA AMRYTEIRLA KIAHELMADL EKETVDFVDK --LNGT-
4-13d              SLVTRIGKYH PHGDFAVYGT IVRMAQPFSL RYMLVDGQGN FGSIDGDSAA AMRYTEIRLA KIAHELMADL EKETVDFVDN --FKRY-
4-30d              LVGDRFGKYH PHGDFAVYGT IVRMAQPFSL RYMLVDGQGN FGSIDGDSAA AMRYTEIRLA KIAHELMADL EKETVDFVDK --CARYG
4-60d              LLVIGIGKYH PHGDFAVYGT IVRMAQPFSL RYMLVDGQGN FGSIDGDSAA AMRYTEIRLA KIAHELMADL EKETVDFVDK -VTVR--
4-90d              -VGHGIGKYH PHGDFAVYGT IVRMAQPFSL RYMLVDGQGN FGSIDGDSAA AMRYTEIRLA KIAHELMADL EKETVDFVDN --WNGT-
Clustal Consensus   :   :**** ********** ********** ********** ********** ********** ********** *********:

Alignment: gyrA5

                   ....|....| ....|....| ....|....| ....|....| ....|....| ....|....| ....|....| ....|....| ....|
                           10         20         30         40         50         60         70         80
4-1d               -VGDVIGKYH PHGDFAVYAT IVRMAQPFSL RYMLVDGQGN FGSIDGDSAA AMRYTEIRLA KIAHELMADL EKETVDFVDN YDGT-
4-7d               -VGEGFGKYH PHGDFAVYAT IVRMAQPFSL RYMLVDGQGN FGSIDGDSAA AMRYTEIRLA KIAHELMADL EKETVDFVDN MSRYG
4-13d              CW-PVIGKYH PHGDFAVYAT IVRMAQPFSL RYMLVDGQGN FGSIDGDSAA AMRYTEIRLA KIAHELMADL EKETVDFVDK RNGT-
4-30d              SLVNGFGKYH PHGDFAVYAT IVRMAQPFSL RYMLVDGQGN FGSIDGDSAA AMRYTEIRLA KIAHELMADL EKETVDFVDK LDGT-
4-60d              -VGDPFGKYH PHGDFAVYAT IVRMAQPFSL RYMLVDGQGN FGSIDGDSAA AMRYTEIRLA KIAHELMADL EKETVDFVDN LGRYG
4-90d              -VGDRIGKYH PHGDFAVYAT IVRMAQPFSL RYMLVDGQGN FGSIDGDSAA AMRYTEIRLA KIAHELMADL EKETVDFVDN WPTVR
Clustal Consensus       :**** ********** ********** ********** ********** ********** ********** *********:
```

Note: “*” means no difference in amino acid. Due to the instability of Sanger sequencing, it is common that there are differences in the first and last 10 amino acids.

Figure S32. PCR results for storage stability of plasmid DNA references materials stored at –20°C.

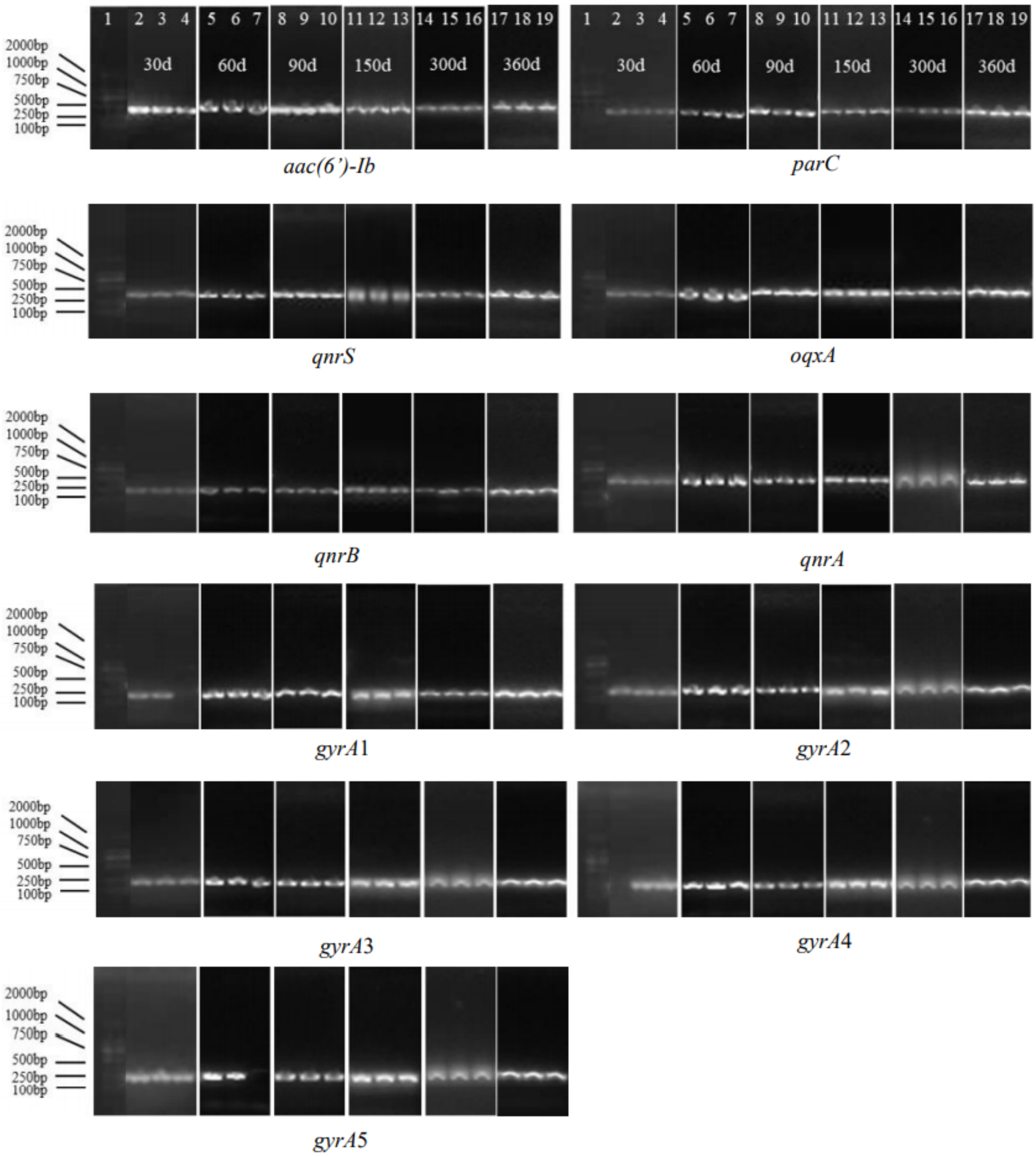

Note: Lane 1 is DL 2000 DNA marker; lane 2–4, 5–7, 8–10, 11–13, 14–16, and 17–19 are PCR bands of the target gene sampled on the 30th, 60th, 90th, 150th, 300th, and 360th days of storage, respectively. Considering the different sampling timepoints, the gene images were intercepted and spliced using Microsoft Word, and the original images are available upon request.

Figure S33. Sequencing results of *aac(6')-Ib* for stability of plasmid DNA references materials stored at –20°C.

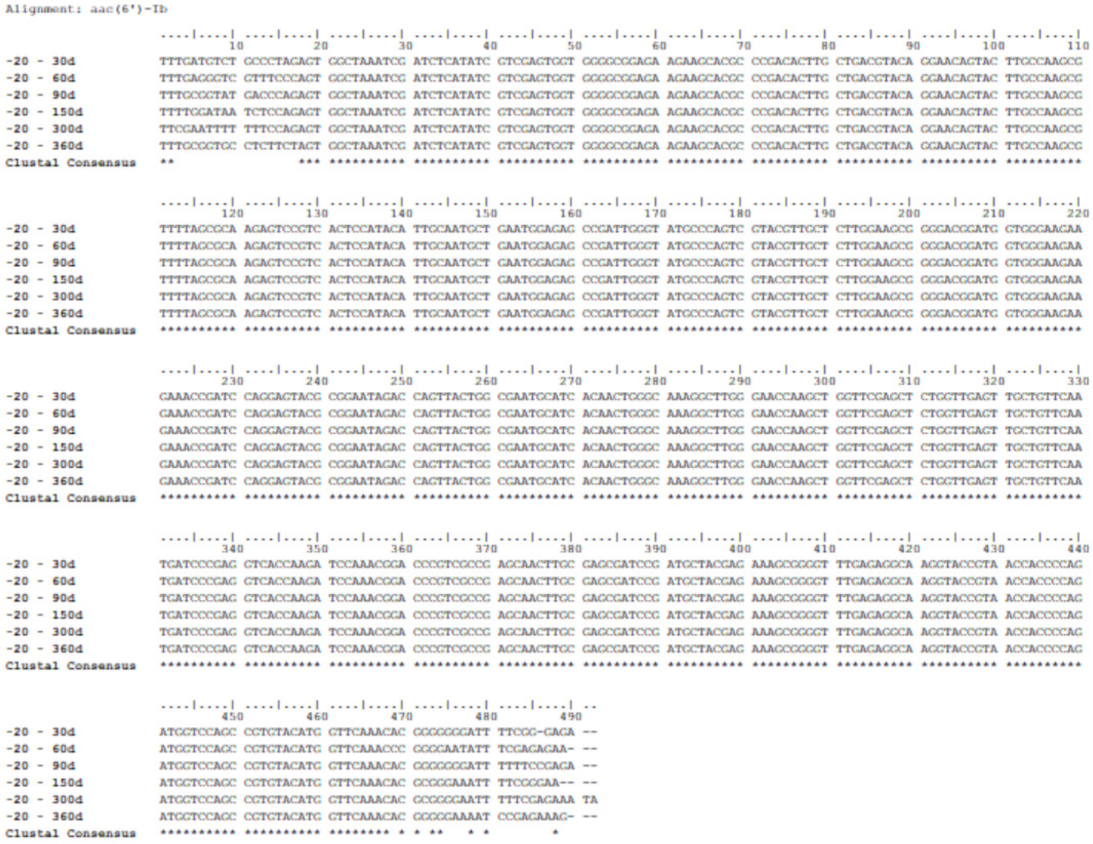

Note: "*" means no difference in nucleotide. Due to the instability of Sanger sequencing, it is common that there are differences in the first and last 20 nucleotides.

Figure S34. Sequencing results of *parC* for stability of plasmid DNA references materials stored at –20°C.

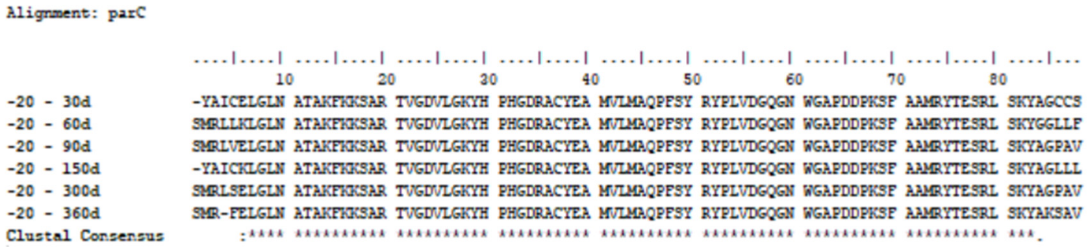

Note: “*” means no difference in amino acid. Due to the instability of Sanger sequencing, it is common that there are differences in the first and last 10 amino acids.

Figure S35. Sequencing results of *qnrS* for stability of plasmid DNA references materials stored at –20°C.

```
Alignment: qnrS

                   ....|....| ....|....| ....|....| ....|....| ....|....| ....|....| ....|....| ....|....| ....|....| ....|....| ....|....|
                           10         20         30         40         50         60         70         80         90        100        110
-20 - 30d          AAGTT--TCT CAATTA-CAG GGTGATATTC GTCGCTGCCA CTTTGATGTC GCAGATCTTC GTGATGCAAG TTTCCAACAA TGCCAACTTG CGATGGCAAA CTTCAGTAAT
-20 - 60d          -AAAT--TTT ACCCTG-CAG GGTGATATTC GTCGCTGCCA CTTTGATGTC GCAGATCTTC GTGATGCAAG TTTCCAACAA TGCCAACTTG CGATGGCAAA CTTCAGTAAT
-20 - 90d          -GTGT--TCT CACATAACAG GGTGATATTC GTCGCTGCCA CTTTGATGTC GCAGATCTTC GTGATGCAAG TTTCCAACAA TGCCAACTTG CGATGGCAAA CTTCAGTAAT
-20 - 150d         -AATTGGTCT CACTTA-CAG GGTGATATTC GTCGCTGCCA CTTTGATGTC GCAGATCTTC GTGATGCAAG TTTCCAACAA TGCCAACTTG CGATGGCAAA CTTCAGTAAT
-20 - 300d         AAGTT--CTT CACTTAACAG GGTGATATTC GTCGCTGCCA CTTTGATGTC GCAGATCTTC GTGATGCAAG TTTCCAACAA TGCCAACTTG CGATGGCAAA CTTCAGTAAT
-20 - 360d         CAATT--TTC CTCCTA-CAG GGTGATATTC GTCGCTGCCA CTTTGATGTC GCAGATCTTC GTGATGCAAG TTTCCAACAA TGCCAACTTG CGATGGCAAA CTTCAGTAAT
Clustal Consensus      *         *  *** ********** ********** ********** ********** ********** ********** ********** ********** **********

                   ....|....| ....|....| ....|....| ....|....| ....|....| ....|....| ....|....| ....|....| ....|....| ....|....| ....|....|
                          120        130        140        150        160        170        180        190        200        210        220
-20 - 30d          GCCAATTGCT ACGGTATAGA GTTCCGTGCG TGTGATTTAA AAGGTGCCAA CTTTTCCCGA ACAAACTTTG CCCATCAAGT GAGTAATCGT ATGTACTTTT GCTCAGCATT
-20 - 60d          GCCAATTGCT ACGGTATAGA GTTCCGTGCG TGTGATTTAA AAGGTGCCAA CTTTTCCCGA ACAAACTTTG CCCATCAAGT GAGTAATCGT ATGTACTTTT GCTCAGCATT
-20 - 90d          GCCAATTGCT ACGGTATAGA GTTCCGTGCG TGTGATTTAA AAGGTGCCAA CTTTTCCCGA ACAAACTTTG CCCATCAAGT GAGTAATCGT ATGTACTTTT GCTCAGCATT
-20 - 150d         GCCAATTGCT ACGGTATAGA GTTCCGTGCG TGTGATTTAA AAGGTGCCAA CTTTTCCCGA ACAAACTTTG CCCATCAAGT GAGTAATCGT ATGTACTTTT GCTCAGCATT
-20 - 300d         GCCAATTGCT ACGGTATAGA GTTCCGTGCG TGTGATTTAA AAGGTGCCAA CTTTTCCCGA ACAAACTTTG CCCATCAAGT GAGTAATCGT ATGTACTTTT GCTCAGCATT
-20 - 360d         GCCAATTGCT ACGGTATAGA GTTCCGTGCG TGTGATTTAA AAGGTGCCAA CTTTTCCCGA ACAAACTTTG CCCATCAAGT GAGTAATCGT ATGTACTTTT GCTCAGCATT
Clustal Consensus  ********** ********** ********** ********** ********** ********** ********** ********** ********** ********** **********

                   ....|....| ....|....| ....|....| ....|....| ....|....| ....|....| ....|....| ....|....| ....|....| ....|....| ....|....|
                          230        240        250        260        270        280        290        300        310        320        330
-20 - 30d          TATTTCTGGA TGTAATCTTT CCTATGCCAA TATGGAGAGG GTTTGTTTAG AAAAATGTGA GTTGTTTGAA AATCGCTGGA TAGGAACGAA CCTAGCGGGT GCATCACTGA
-20 - 60d          TATTTCTGGA TGTAATCTTT CCTATGCCAA TATGGAGAGG GTTTGTTTAG AAAAATGTGA GTTGTTTGAA AATCGCTGGA TAGGAACGAA CCTAGCGGGT GCATCACTGA
-20 - 90d          TATTTCTGGA TGTAATCTTT CCTATGCCAA TATGGAGAGG GTTTGTTTAG AAAAATGTGA GTTGTTTGAA AATCGCTGGA TAGGAACGAA CCTAGCGGGT GCATCACTGA
-20 - 150d         TATTTCTGGA TGTAATCTTT CCTATGCCAA TATGGAGAGG GTTTGTTTAG AAAAATGTGA GTTGTTTGAA AATCGCTGGA TAGGAACGAA CCTAGCGGGT GCATCACTGA
-20 - 300d         TATTTCTGGA TGTAATCTTT CCTATGCCAA TATGGAGAGG GTTTGTTTAG AAAAATGTGA GTTGTTTGAA AATCGCTGGA TAGGAACGAA CCTAGCGGGT GCATCACTGA
-20 - 360d         TATTTCTGGA TGTAATCTTT CCTATGCCAA TATGGAGAGG GTTTGTTTAG AAAAATGTGA GTTGTTTGAA AATCGCTGGA TAGGAACGAA CCTAGCGGGT GCATCACTGA
Clustal Consensus  ********** ********** ********** ********** ********** ********** ********** ********** ********** ********** **********

                   ....|....| ....|....| ....|....| ....|....| ....|....| ....|....| ....|....| ....|....| ....|....| ....|....| ....|...
                          340        350        360        370        380        390        400        410        420        430
-20 - 30d          AAGAGTCAGA CTTAAGTCGA GGTGTTTTTT CCGAAGATGT CTGGGGGCAA TTTAGCCTAC AGGGGGCCAA TTTATGCCAC GCCGAATGA- --ATGGGGTT T-AGAA--
-20 - 60d          AAGAGTCAGA CTTAAGTCGA GGTGTTTTTT CCGAAGATGT CTGGGGGCAA TTTAGCCTAC AGGGGGCCAA TTTATGCCAC GCCGATGGAC --GGGGAGTT TTAAAAAA
-20 - 90d          AAGAGTCAGA CTTAAGTCGA GGTGTTTTTT CCGAAGATGT CTGGGGGCAA TTTAGCCTAC AGGGGGCCAA TTTATGCCAC GCCGAATGA- --CGGGGGTT TTAGAA--
-20 - 150d         AAGAGTCAGA CTTAAGTCGA GGTGTTTTTT CCGAAGATGT CTGGGGGCAA TTTAGCCTAC AGGGGGCCAA TTTATGCCAC GCCGAATGGC GCGAGAAGGT TTAGAAGA
-20 - 300d         AAGAGTCAGA CTTAAGTCGA GGTGTTTTTT CCGAAGATGT CTGGGGGCAA TTTAGCCTAC AGGGGGCCAA TTTATGCCAC GCCGAATGG- ---CGGGGTT T-AGAA--
-20 - 360d         AAGAGTCAGA CTTAAGTCGA GGTGTTTTTT CCGAAGATGT CTGGGGGCAA TTTAGCCTAC AGGGGGCCAA TTTATGCCAC GCCGAATGGC --AGGGGGTT TAAAAA--
Clustal Consensus  ********** ********** ********** ********** ********** ********** ********** ********** *****  *        *  * * * * **
```

Note: “*” means no difference in nucleotide. Due to the instability of Sanger sequencing, it is common that there are differences in the first and last 20 nucleotides.

Figure S36. Sequencing results of *oqxA* for stability of plasmid DNA references materials stored at –20°C.

```
Alignment: oqxA

                   ....|....| ....|....| ....|....| ....|....| ....|....| ....|....| ....|....| ....|....| ....|....| ....|....| ....|....|
                          10         20         30         40         50         60         70         80         90        100        110
-20 - 30d          --CAGC-TGT CCCCAAGAAT GCTGCGCCTC CCGCCCCGAC GGTCAGCGCC GCTAAGGTGC TGGTGAAGTC GATCAGTCAG TGGGATAGTT TTAACGGTCG CATTGAAGCG
-20 - 60d          ACCAGGGTAT GATC-CGAAT GCTGCGCCTC CCGCCCCGAC GGTCAGCGCC GCTAAGGTGC TGGTGAAGTC GATCAGTCAG TGGGATAGTT TTAACGGTCG CATTGAAGCG
-20 - 90d          -ACAGGCGTG TTCGCAGAAT GCTGCGCCTC CCGCCCCGAC GGTCAGCGCC GCTAAGGTGC TGGTGAAGTC GATCAGTCAG TGGGATAGTT TTAACGGTCG CATTGAAGCG
-20 - 150d         GACAGCGTTT GTCC-AGAAT GCTGCGCCTC CCGCCCCGAC GGTCAGCGCC GCTAAGGTGC TGGTGAAGTC GATCAGTCAG TGGGATAGTT TTAACGGTCG CATTGAAGCG
-20 - 300d         GACAGCGTTG TCCC-AGAAT GCTGCGCCTC CCGCCCCGAC GGTCAGCGCC GCTAAGGTGC TGGTGAAGTC GATCAGTCAG TGGGATAGTT TTAACGGTCG CATTGAAGCG
-20 - 360d         GACAGCGTTT GCCCCAGAAT GCTGCGCCTC CCGCCCCGAC GGTCAGCGCC GCTAAGGTGC TGGTGAAGTC GATCAGTCAG TGGGATAGTT TTAACGGTCG CATTGAAGCG
Clustal Consensus     ***           **** ********** ********** ********** ********** ********** ********** ********** ********** **********

                   ....|....| ....|....| ....|....| ....|....| ....|....| ....|....| ....|....| ....|....| ....|....| ....|....| ....|....|
                         120        130        140        150        160        170        180        190        200        210        220
-20 - 30d          GTGGAGAGCG TTCAGCTTCG CCCTCGCGTC TCGGGATACA TTGATAAAGT GAATTACACT GACGGCCAGG AGGTGAAAAA GGGCCAGGTG CTGTTCACGA TAGATGACAG
-20 - 60d          GTGGAGAGCG TTCAGCTTCG CCCTCGCGTC TCGGGATACA TTGATAAAGT GAATTACACT GACGGCCAGG AGGTGAAAAA GGGCCAGGTG CTGTTCACGA TAGATGACAG
-20 - 90d          GTGGAGAGCG TTCAGCTTCG CCCTCGCGTC TCGGGATACA TTGATAAAGT GAATTACACT GACGGCCAGG AGGTGAAAAA GGGCCAGGTG CTGTTCACGA TAGATGACAG
-20 - 150d         GTGGAGAGCG TTCAGCTTCG CCCTCGCGTC TCGGGATACA TTGATAAAGT GAATTACACT GACGGCCAGG AGGTGAAAAA GGGCCAGGTG CTGTTCACGA TAGATGACAG
-20 - 300d         GTGGAGAGCG TTCAGCTTCG CCCTCGCGTC TCGGGATACA TTGATAAAGT GAATTACACT GACGGCCAGG AGGTGAAAAA GGGCCAGGTG CTGTTCACGA TAGATGACAG
-20 - 360d         GTGGAGAGCG TTCAGCTTCG CCCTCGCGTC TCGGGATACA TTGATAAAGT GAATTACACT GACGGCCAGG AGGTGAAAAA GGGCCAGGTG CTGTTCACGA TAGATGACAG
Clustal Consensus  ********** ********** ********** ********** ********** ********** ********** ********** ********** ********** **********

                   ....|....| ....|....| ....|....| ....|....| ....|....| ....|....| ....|....| ....|....| ....|....| ....|....| ....|....|
                         230        240        250        260        270        280        290        300        310        320        330
-20 - 30d          AACCTATCGC GCCGCGCTGG AGCAGGCGCA GGCGGCGTTG GCAAGAGCCA AAACGCAGGC CAGCCTCGCG CAAAGCGAGG CGAACCGCAC CGATAAATTA GTCCATACC-
-20 - 60d          AACCTATCGC GCCGCGCTGG AGCAGGCGCA GGCGGCGTTG GCAAGAGCCA AAACGCAGGC CAGCCTCGCG CAAAGCGAGG CGAACCGCAC CGATAAATTA GTCCATATCC
-20 - 90d          AACCTATCGC GCCGCGCTGG AGCAGGCGCA GGCGGCGTTG GCAAGAGCCA AAACGCAGGC CAGCCTCGCG CAAAGCGAGG CGAACCGCAC CGATAAATTA GTCCATACC-
-20 - 150d         AACCTATCGC GCCGCGCTGG AGCAGGCGCA GGCGGCGTTG GCAAGAGCCA AAACGCAGGC CAGCCTCGCG CAAAGCGAGG CGAACCGCAC CGATAAATTA GTCCATACAA
-20 - 300d         AACCTATCGC GCCGCGCTGG AGCAGGCGCA GGCGGCGTTG GCAAGAGCCA AAACGCAGGC CAGCCTCGCG CAAAGCGAGG CGAACCGCAC CGATAAATTA GTCCATAC--
-20 - 360d         AACCTATCGC GCCGCGCTGG AGCAGGCGCA GGCGGCGTTG GCAAGAGCCA AAACGCAGGC CAGCCTCGCG CAAAGCGAGG CGAACCGCAC CGATAAATTA GTCCATACC-
Clustal Consensus  ********** ********** ********** ********** ********** ********** ********** ********** ********** ********** *******

                   ....|....| ....|....
                         340
-20 - 30d          CCCCTCTCGT CTCCTGA--
-20 - 60d          CCCCCCCGTC CTCATAAA-
-20 - 90d          CCCCCTCCGT CTCCC----
-20 - 150d         CCATTTTGGT CTCCCATGA
-20 - 300d         CCCGTCTCGT CTCCCAGC-
-20 - 360d         CCCCTC--GT CTCCT----
Clustal Consensus  **         ***
```

Note: “*” means no difference in nucleotide. Due to the instability of Sanger sequencing, it is common that there are differences in the first and last 20 nucleotides.

Figure S37. Sequencing results of *qnrB* for stability of plasmid DNA references materials stored at –20°C.

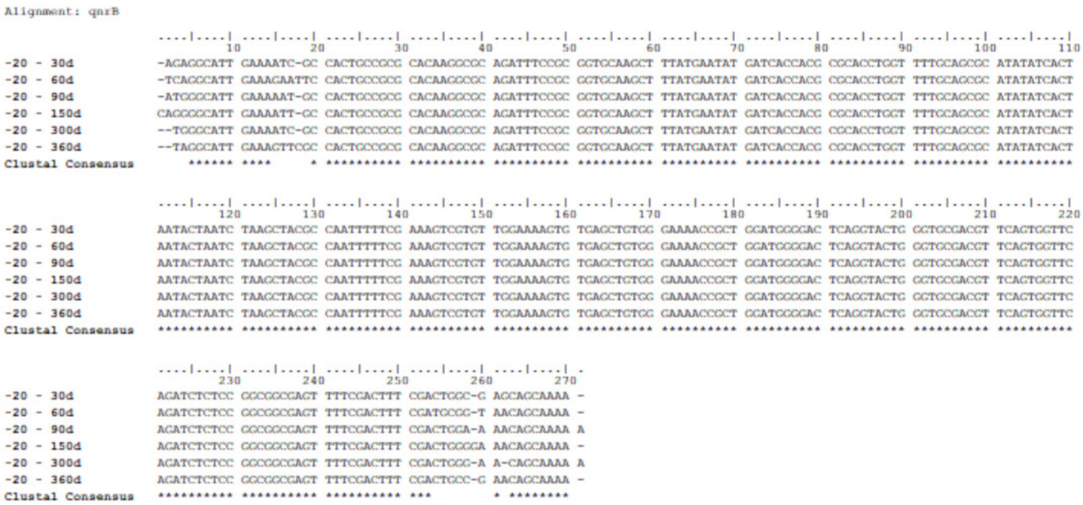

Note: “*” means no difference in nucleotide. Due to the instability of Sanger sequencing, it is common that there are differences in the first and last 20 nucleotides.

Figure S38. Sequencing results of *qnrA* for stability of plasmid DNA references materials stored at –20°C.

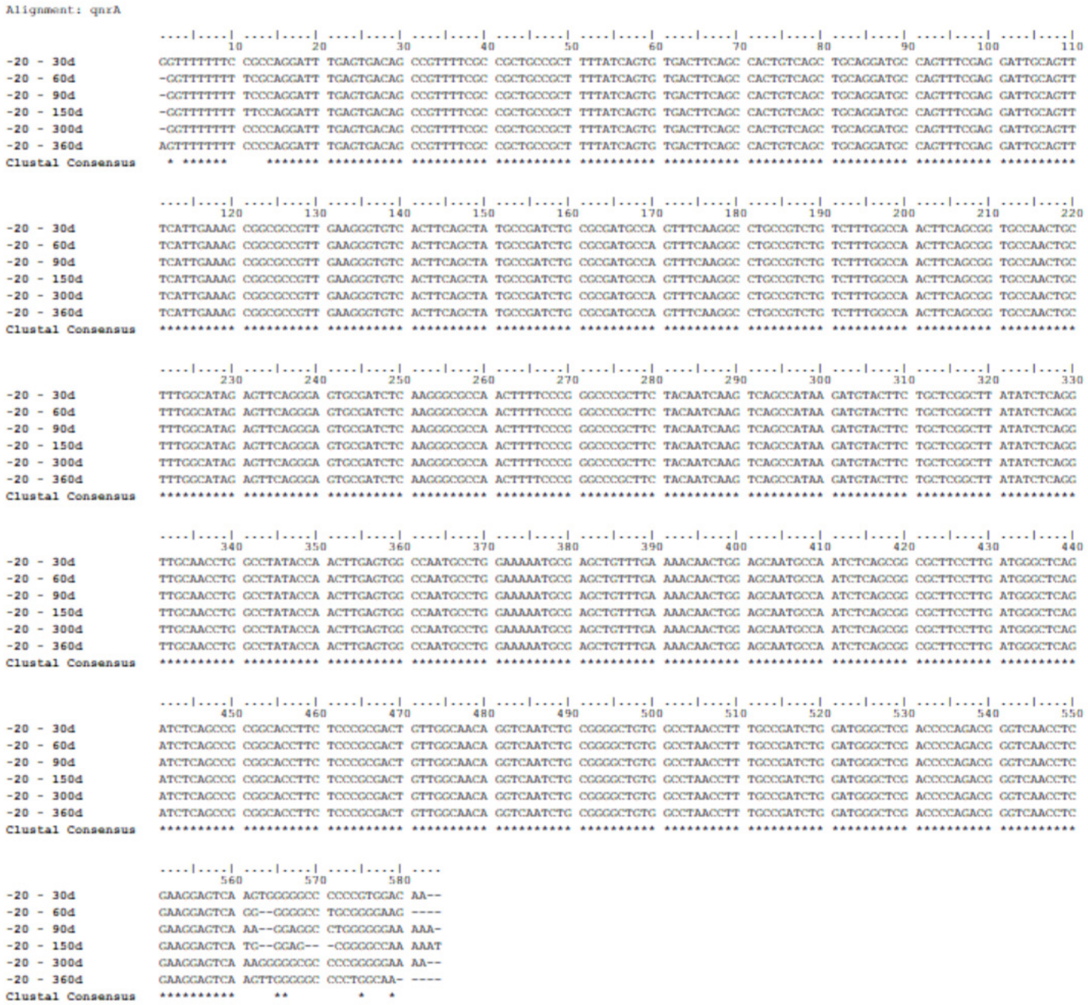

Note: "*" means no difference in nucleotide. Due to the instability of Sanger sequencing, it is common that there are differences in the first and last 20 nucleotides.

Figure S39. Sequencing results of *gyrA* for stability of plasmid DNA references materials stored at –20°C.

```
Alignment: gyrA1

                         10         20         30         40         50         60         70         80
-20 - 30d        -LLVNLFGKY HPHGDSAVYN TIVRMAQPFS LRYMLVDGQG NFGSIDGDSA AAMRYTEIRL AKIAHELMAD LEKETVDFVD KRERYG
-20 - 60d        --RW-GFGKY HPHGDSAVYN TIVRMAQPFS LRYMLVDGQG NFGSIDGDSA AAMRYTEIRL AKIAHELMAD LEKETVDFVD KMGTVR
-20 - 90d        -RW-SRIGKY HPHGDSAVYN TIVRMAQPFS LRYMLVDGQG NFGSIDGDSA AAMRYTEIRL AKIAHELMAD LEKETVDFVD NWNGT-
-20 - 150d       -SLATRIGKY HPHGDSAVYN TIVRMAQPFS LRYMLVDGQG NFGSIDGDSA AAMRYTEIRL AKIAHELMAD LEKETVDFVD KTERY-
-20 - 300d       -RW-PRFGKY HPHGDSAVYN TIVRMAQPFS LRYMLVDGQG NFGSIDGDSA AAMRYTEIRL AKIAHELMAD LEKETVDFVD TLTVR-
-20 - 360d       LVG-DRFGKY HPHGDSAVYN TIVRMAQPFS LRYMLVDGQG NFGSIDGDSA AAMRYTEIRL AKIAHELMAD LEKETVDFVD KIDGT-
Clustal Consensus       :*** ********** ********** ********** ********** ********** ********** ********** .

Alignment: gyrA2

                         10         20         30         40         50         60         70         80
-20 - 30d        -VADRFGKYH PHGDYAVYDT IVRMAQPFSL RYMLVDGQGN FGSIDGDSAA AMRYTEIRLA KIAHELMADL EKETVDFVDN NDGT-
-20 - 60d        RW--SVGKYH PHGDYAVYDT IVRMAQPFSL RYMLVDGQGN FGSIDGDSAA AMRYTEIRLA KIAHELMADL EKETVDFVDK LQTVR
-20 - 90d        SLVNGIGKYH PHGDYAVYDT IVRMAQPFSL RYMLVDGQGN FGSIDGDSAA AMRYTEIRLA KIAHELMADL EKETVDFVDN WDGT-
-20 - 150d       RWSSAFGKYH PHGDYAVYDT IVRMAQPFSL RYMLVDGQGN FGSIDGDSAA AMRYTEIRLA KIAHELMADL EKETVDFVDK SNGT-
-20 - 300d       -VADRFGKYH PHGDYAVYDT IVRMAQPFSL RYMLVDGQGN FGSIDGDSAA AMRYTEIRLA KIAHELMADL EKETVDFVDN NDGT-
-20 - 360d       -VGDGFGKYH PHGDYAVYDT IVRMAQPFSL RYMLVDGQGN FGSIDGDSAA AMRYTEIRLA KIAHELMADL EKETVDFVDK SDGT-
Clustal Consensus      .**** ********** ********** ********** ********** ********** ********** *********:  : .

Alignment: gyrA3

                         10         20         30         40         50         60         70         80
-20 - 30d        -LVTRFGKYH PHGDFAVYDT IVRMAQPFSL RYMLVDGQGN FGSIDGDSAA AMRYTEIRLA KIAHELMADL EKETVDFVDK MGRYG
-20 - 60d        HW-PRFGKYH PHGDFAVYDT IVRMAQPFSL RYMLVDGQGN FGSIDGDSAA AMRYTEIRLA KIAHELMADL EKETVDFVDT WERY-
-20 - 90d        -VGEPIGKYH PHGDFAVYDT IVRMAQPFSL RYMLVDGQGN FGSIDGDSAA AMRYTEIRLA KIAHELMADL EKETVDFVDN -QTVR
-20 - 150d       SLVVGFGKYH PHGDFAVYDT IVRMAQPFSL RYMLVDGQGN FGSIDGDSAA AMRYTEIRLA KIAHELMADL EKETVDFVDK SNGT-
-20 - 300d       SVGARVGKYH PHGDFAVYDT IVRMAQPFSL RYMLVDGQGN FGSIDGDSAA AMRYTEIRLA KIAHELMADL EKETVDFVDK M-TVR
-20 - 360d       -VGDRFGKYH PHGDFAVYDT IVRMAQPFSL RYMLVDGQGN FGSIDGDSAA AMRYTEIRLA KIAHELMADL EKETVDFVDK IDGT-
Clustal Consensus      .**** ********** ********** ********** ********** ********** ********** *********.

Alignment: gyrA4

                         10         20         30         40         50         60         70         80
-20 - 30d        -VGERFGKYH PHGDFAVYGT IVRMAQPFSL RYMLVDGQGN FGSIDGDSAA AMRYTEIRLA KIAHELMADL EKETVDFVDK LNGT--
-20 - 60d        -VGERFGKYH PHGDFAVYGT IVRMAQPFSL RYMLVDGQGN FGSIDGDSAA AMRYTEIRLA KIAHELMADL EKETVDFVDK METVR-
-20 - 90d        -CW-PIGKYH PHGDFAVYGT IVRMAQPFSL RYMLVDGQGN FGSIDGDSAA AMRYTEIRLA KIAHELMADL EKETVDFVDN -SNGTD
-20 - 150d       SLVRGFGKYH PHGDFAVYGT IVRMAQPFSL RYMLVDGQGN FGSIDGDSAA AMRYTEIRLA KIAHELMADL EKETVDFVDK -MNGT-
-20 - 300d       -VGERIGKYH PHGDFAVYGT IVRMAQPFSL RYMLVDGQGN FGSIDGDSAA AMRYTEIRLA KIAHELMADL EKETVDFVDN LARYG-
-20 - 360d       LLVIGIGKYH PHGDFAVYGT IVRMAQPFSL RYMLVDGQGN FGSIDGDSAA AMRYTEIRLA KIAHELMADL EKETVDFVDK -VTVR-
Clustal Consensus      :**** ********** ********** ********** ********** ********** ********** *********:

Alignment: gyrA5

                         10         20         30         40         50         60         70         80
-20 - 30d        -VGEGFGKYH PHGDFAVYAT IVRMAQPFSL RYMLVDGQGN FGSIDGDSAA AMRYTEIRLA KIAHELMADL EKETVDFVDN MSRYG
-20 - 60d        SLVIGYGKYH PHGDFAVYAT IVRMAQPFSL RYMLVDGQGN FGSIDGDSAA AMRYTEIRLA KIAHELMADL EKETVDFVDT ANGT-
-20 - 90d        -LVHRIGKYH PHGDFAVYAT IVRMAQPFSL RYMLVDGQGN FGSIDGDSAA AMRYTEIRLA KIAHELMADL EKETVDFVDN WNGT-
-20 - 150d       SLVVGFGKYH PHGDFAVYAT IVRMAQPFSL RYMLVDGQGN FGSIDGDSAA AMRYTEIRLA KIAHELMADL EKETVDFVDN C-RY-
-20 - 300d       RW-AGIGKYH PHGDFAVYAT IVRMAQPFSL RYMLVDGQGN FGSIDGDSAA AMRYTEIRLA KIAHELMADL EKETVDFVDN LDGT-
-20 - 360d       -VGDRIGKYH PHGDFAVYAT IVRMAQPFSL RYMLVDGQGN FGSIDGDSAA AMRYTEIRLA KIAHELMADL EKETVDFVDN WPTVR
Clustal Consensus       **** ********** ********** ********** ********** ********** ********** *********.
```

Note: “*” means no difference in amino acid. Due to the instability of Sanger sequencing, it is common that there are differences in the first and last 10 amino acids.
